# Benchmark indicators for evaluating sea turtle rehabilitation outcomes at a wildlife rehabilitation center on Tenerife island, Spain (1982–2025)

**DOI:** 10.3389/fvets.2026.1948954

**Published:** 2026-10-19

**Authors:** Felipe Trujillo-Camejo, Natalia Montesdeoca, Sara Capelli, Carmen D. Méndez-Corona, Jorge Orós

**Affiliations:** 1 La Tahonilla Wildlife Rehabilitation Center (Cabildo de Tenerife), San Cristóbal de La Laguna, Santa Cruz de Tenerife, Spain; 2 Department of Morphology, Veterinary Faculty, University of Las Palmas de Gran Canaria (ULPGC), Arucas, Las Palmas, Spain

**Keywords:** benchmark indicators, conservation medicine, cost analysis, evidence-based conservation, rehabilitation outcomes, sea turtles, wildlife rehabilitation

## Abstract

**Background:**

Sea turtle rehabilitation is widely practiced worldwide, yet standardized benchmark indicators for objectively evaluating rehabilitation quality are currently lacking. This study aimed to establish and evaluate a set of benchmark indicators for assessing rehabilitation outcomes in sea turtles while providing long-term epidemiological information on the principal causes of admission to a wildlife rehabilitation center.

**Methods:**

A retrospective study included 2,278 sea turtle admission records from the La Tahonilla Wildlife Rehabilitation Center (LTWRC), Tenerife, Spain, between 1982 and 2025. After excluding eight readmission records, the first-admission dataset comprised 2,270 individual turtles: 2,216 loggerhead turtles (*Caretta caretta*) and 54 green turtles (*Chelonia mydas*). Rehabilitation performance was evaluated using cause-specific benchmark indicators, including euthanasia (E_r_), unassisted mortality during hospitalization (M_r_), and release (R_r_) rates, time to final disposition, post-release readmissions, and standardized rehabilitation costs.

**Results:**

Entanglement in fishing gear and/or plastic materials was the most common cause at first admission (42.8%), whereas unknown/undetermined causes predominated among turtles dead on first admission (63.9%). Outcomes for turtles alive at first admission were E_r_ = 1.4%, M_r_ = 12.5%, and R_r_ = 86%. Rehabilitation outcome was strongly influenced by the primary cause of admission, with entanglement associated with significantly higher odds of release, whereas trauma, hook/monofilament ingestion, and infectious disease were associated with significantly higher unassisted mortality. Among hospitalization episodes resulting in release, median rehabilitation time ranged from 10 days for crude oil exposure to 37 days for trauma. Post-release readmissions were uncommon (16/1,560 turtles; 1.0%), accounting for 22 readmission events; all 20 events involving turtles admitted alive resulted in release. Rehabilitation costs differed mainly according to the diagnostic and therapeutic procedures required, whereas fixed costs represented the largest cost component in most admission categories.

**Conclusion:**

This study provides one of the largest and longest-term evaluations of sea turtle rehabilitation to date and establishes practical benchmark indicators for objectively assessing rehabilitation quality. Beyond their clinical relevance, these indicators provide an evidence-based framework to improve rehabilitation management, optimize resource allocation, and strengthen the contribution of veterinary medicine to wildlife conservation, conservation management, and evidence-based policy development.

## 1 Introduction

Currently, seven extant species of sea turtles, representing two taxonomic families, are recognized (1), and all are listed on the International Union for Conservation of Nature’s (IUCN) Red List of Threatened Species (2). The loggerhead turtle (*Caretta caretta*) is the sea turtle species most frequently recorded in the Canary Islands, where most individuals belong to the oceanic juvenile developmental stage, which is widely distributed throughout the northeastern Atlantic Ocean (3). Additionally, a limited number of juvenile green turtles (*Chelonia mydas*) occur along the Canary coasts, where they are recognized as temporary residents of the archipelago (3). Sea turtle strandings occur frequently in the Canary Islands, and several studies have documented the main diseases and causes of mortality or stranding affecting individuals found along the archipelago’s coastline, with anthropogenic factors, particularly entanglement in fishing gear and plastic debris, ingestion of fishing hooks and monofilament lines, and vessel-related trauma, identified as major causes (4–7).

Wildlife rehabilitation may support species conservation through the reintroduction of clinically recovered individuals into their natural habitats and by increasing public awareness of the threats affecting wildlife populations (8). Some researchers have cautioned that wildlife rehabilitation programs require substantial financial investment and that, under certain circumstances, these resources could potentially yield greater conservation benefits if allocated to preventive strategies designed to reduce wildlife injury (9). Available data on sea turtle rehabilitation success remain scarce and generally indicate that only a relatively small proportion of admitted individuals are ultimately returned to the wild (8, 10, 11). Furthermore, limited information is available regarding the successful post-release readaptation of rehabilitated sea turtles, particularly in individuals subjected to prolonged and complex veterinary treatment (6, 12–15) (Supplementary Table S1).

The primary aim of this study was to evaluate rehabilitation outcomes of sea turtles admitted to the La Tahonilla Wildlife Rehabilitation Center (LTWRC), Tenerife, Spain, between 1982 and 2025 using a set of specific indicators, including cause-specific stratified analyses of the final disposition rates (euthanasia, unassisted mortality during hospitalization, and release), time to death for euthanized and deceased turtles, length of stay for released individuals, post-release readmissions and outcomes, and financial cost per released turtle. In addition, causes of admission were analyzed using standardized epidemiological data to facilitate comparisons with other geographic regions.

## 2 Materials and methods

### 2.1 Ethics approval statement

The sea turtle rehabilitation program at the LTWRC was conducted with authorization from the Wildlife Department of the Canary Islands Government, and the Environment Department of the Cabildo de Tenerife. All procedures involving animals and sample collection were approved by the Cabildo de Tenerife and performed in compliance with recognized vertebrate research protocols and veterinary standards. The anesthetic protocol most frequently used consisted of propofol (5–7 mg/kg IV) for induction, followed by maintenance with isoflurane (2%) administered through an endotracheal tube. Alternatively, anesthesia was induced using a combination of ketamine (1-2 mg/kg IV) and dexmedetomidine (0.05 mg/kg IV), with butorphanol (0.4 mg/kg IM) administered to provide additional analgesia. Euthanasia was considered when the attending veterinarian determined that the animal had a poor welfare prognosis or that the severity or irreversible nature of its condition made successful rehabilitation and post-release survival unlikely. When euthanasia was deemed necessary, sea turtles were euthanized by intravenous administration of an overdose of sodium pentobarbital (250 mg/kg IV), in accordance with accepted veterinary euthanasia principles (16). The present study involved exclusively the retrospective analysis of existing clinical records and did not involve any additional procedures or interventions on animals for research purposes. Therefore, specific ethical review and approval for the retrospective study were not required.

### 2.2 Animals and study area

A retrospective study was conducted using 2,285 loggerhead turtle and 59 green turtle admission records from the LTWRC on Tenerife Island, Spain, between April 1982 and December 2025. Due to incomplete medical records, 65 loggerheads and 1 green turtle were excluded from the study. Among the remaining 2,278 admission records, eight records (four loggerhead turtles and four green turtles) corresponded to readmissions of individuals already represented by a previous admission. These records were excluded from the first-admission dataset and retained for the analysis of readmission events. Consequently, 2,270 unique individual turtles were included in the final first-admission dataset: 2,216 loggerheads (97.62%) and 54 green turtles (2.38%). The case-selection process and the numbers of turtles included at each stage of the study are summarized in Figure 1. The LTWRC receives turtles stranded in Tenerife and, occasionally, from other smaller islands of the Canary archipelago. Tenerife (28°00′–28°35′N and 16°08′–16°46′W) is the largest island (2,034 km^2^) in the Canary archipelago and has a coastline of 342 km.

**Figure 1:**
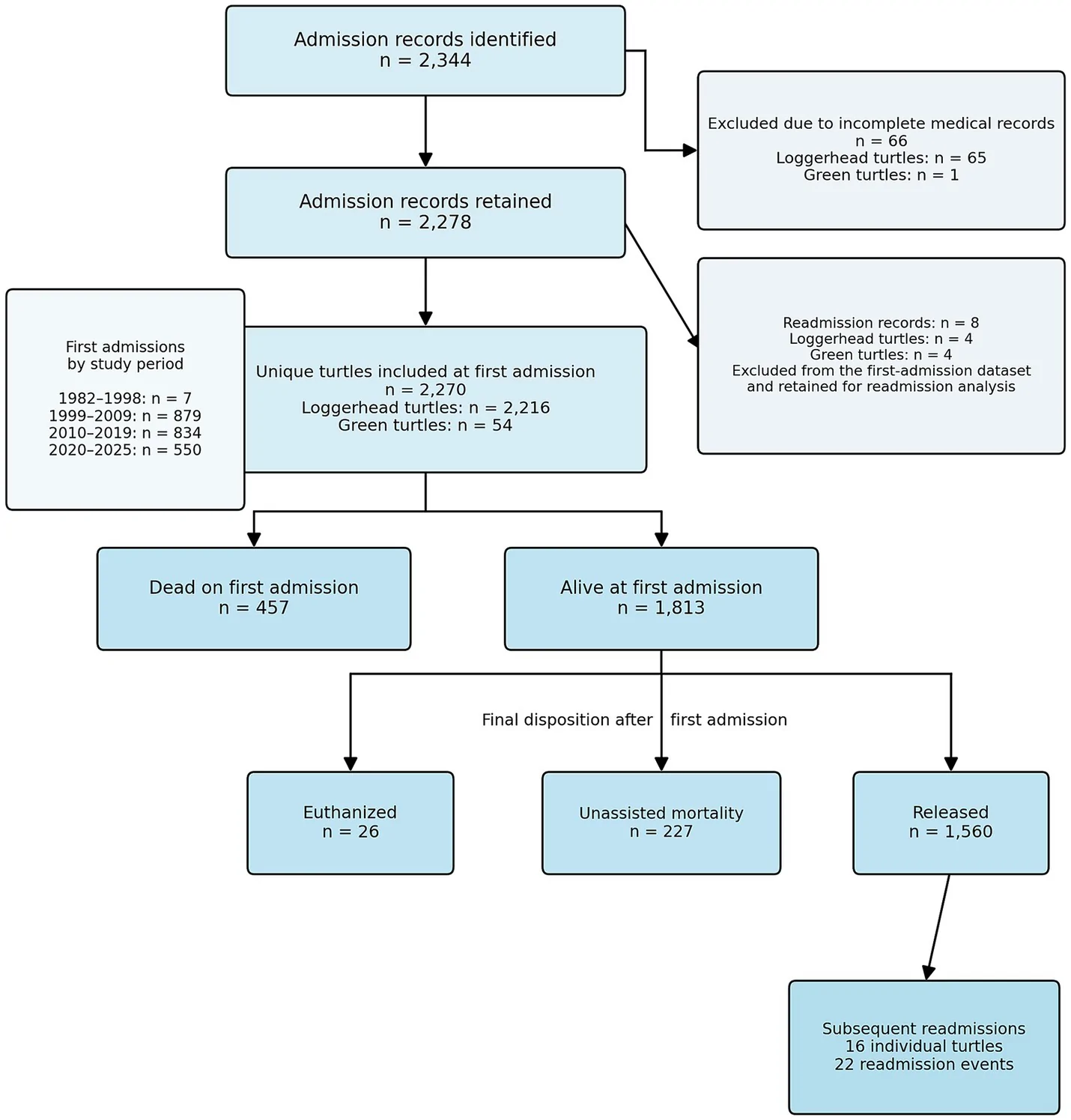
Flow diagram of case selection, first-admission outcomes, and subsequent readmissions of loggerhead and green turtles admitted to the La Tahonilla Wildlife Rehabilitation Center from 1982 to 2025. Of 2,344 admission records identified, 66 were excluded because of incomplete medical records. Eight of the remaining 2,278 records represented readmissions and were excluded from the first-admission dataset but retained for the analysis of readmission events, resulting in 2,270 individual turtles included at first admission. The distribution of first admissions across study periods is also shown. Subsequent readmissions are reported both as the number of individual turtles and the number of readmission events.

### 2.3 Variables analyzed

For each admission event, species, straight carapace length (SCL), body weight at admission, stranding location, date of admission, primary cause of admission, and outcome including the corresponding date of release or death, were recorded. Stranding location was recorded as part of the routine case documentation but was not included in the analyses performed in the present study. Sex was not included in the analyses because it had been recorded for only a small subset of necropsied turtles. SCL measurements were available for 1,099 turtles, whereas both SCL and body weight measurements required for body condition index (BCI) calculation were available for 1,000 turtles. When both SCL and body weight at admission were available, BCI was calculated as BCI = [body weight (kg)/SCL^3^ (cm^3^)] × 10,000 (17, 18). Based on SCL measurements and following previously published criteria (19, 20), individuals were classified into three life-stage categories: pelagic juveniles (SCL < 42 cm for loggerheads and SCL < 65 cm for green turtles), juveniles/subadults (42 cm ≤ SCL < 70 cm for loggerheads and 65 cm ≤ SCL < 90 cm for green turtles), and adults (SCL ≥ 70 cm for loggerheads and SCL ≥ 90 cm for green turtles). The de-identified case-level dataset underlying the analyses is provided in Supplementary Data Sheet S1.

The primary cause of admission was initially established by the veterinarian responsible for each case. For the purposes of the present study, the authors retrospectively reviewed the clinical records and available ancillary diagnostic findings and assigned each case to one of the predefined admission categories. In cases where multiple conditions were present simultaneously, the condition considered primarily responsible for requiring clinical intervention was selected as the predominant morbidity factor and included in the analysis (6). Admission causes were grouped into seven categories: entanglement in fishing gear and/or plastic materials (including synthetic raffia), ingestion of fishing hooks and monofilament line, traumatic injury associated with vessel collisions, infectious disease, crude-oil exposure, other causes, and unknown/undetermined causes. Entanglement was defined as external entrapment or constriction caused by fishing gear or plastic materials, including synthetic raffia, ropes, nets, monofilament lines, and other plastic debris. This category included turtles retrieved while entangled in fishing gear as well as turtles admitted with clinical lesions attributable to previous entanglement, even when the constricting material had already been removed before admission. Because the historical medical records did not consistently distinguish active fishing gear from lost or discarded (derelict) fishing gear, these situations were analyzed within the same entanglement category. Cases were assigned to the infectious disease category only when the involvement of a pathogenic microorganism was demonstrated through microbiological testing, PCR, or histopathological examination; cases based solely on clinical or presumptive diagnoses were not included in this category. The “other causes” category encompassed plastic ingestion, buoyancy abnormalities, shark-inflicted injuries, malnutrition, and other infrequent conditions or “miscellany”. Malnutrition was defined as the presence of cachexia in turtles for which no additional lesions or pathological findings could account for the poor body condition.

The fate of turtles admitted alive was assessed by assigning each case to one of three outcome groups: turtles that were euthanized, died unassisted during hospitalization, or were released back into the wild. Euthanasia was performed when the expected quality of life and/or likelihood of post-release survival were considered poor. Outcome proportions were then calculated for the entire cohort of turtles alive at first admission, including the euthanasia rate (E_r_), unassisted mortality rate during hospitalization (M_r_), and release rate (R_r_). The same outcome measures were also determined for each admission category.

The time elapsed from admission to death was calculated for turtles that were euthanized or died unassisted during hospitalization (T_d_), whereas the length of rehabilitation was determined for released individuals (T_r_), using the interval between admission and release dates. For turtles with subsequent readmissions, each hospitalization was considered a separate rehabilitation episode for the calculation of T_d_ or T_r_. These variables were analyzed according to the cause of admission. Furthermore, the 10th, 25th, 50th, 75th, and 90th percentiles (P_10_, P_25_, P_50_, P_75_, and P_90_) were estimated for both T_d_ and T_r_ distributions. These time-to-disposition variables were analyzed descriptively within each final outcome group and were intended to provide benchmark indicators of rehabilitation duration rather than covariate-adjusted estimates of time to disposition.

Post-release readmissions were evaluated whenever they occurred during the study period. A readmission was defined as any subsequent admission of a turtle previously released from the LTWRC, as confirmed by flipper tag or passive integrated transponder (PIT) tag identification. For each readmission event, species, cause of admission, outcome, and the interval since the preceding release were recorded. Individual turtles could contribute more than one readmission event. The cause of each readmission was also compared with that of the immediately preceding admission or readmission. Readmission events resulting in hospitalization were additionally considered as separate rehabilitation episodes in the descriptive analyses of T_d_ and T_r_ described above.

To estimate the economic cost of sea turtle rehabilitation, costs were categorized into three components: fixed costs, admission-related costs, and variable costs. For resources shared among the different wildlife species treated at the center, including veterinary personnel, the proportion of costs attributable to sea turtle rehabilitation was estimated according to the proportion of sea turtles within the total annual wildlife caseload (approximately 5.3%). Fixed costs included annual expenses associated with the operation of the rehabilitation facilities, such as pool maintenance, electricity, and personnel costs related to sea turtle care. Admission-related costs included expenses incurred for the retrieval and transportation of stranded turtles and the routine veterinary assessment performed at admission, including physical examination, basic laboratory testing, routine radiography, and standard stabilization procedures. Variable costs included feeding expenses and all diagnostic and therapeutic procedures required during hospitalization, such as advanced diagnostic imaging, microbiological culture, PCR testing, endoscopy, anesthesia, surgery, medications, and other specialized treatments.

Rehabilitation costs were estimated using current values provided by the rehabilitation center and should therefore be considered standardized cost estimates. Fixed costs were allocated on a per-admission basis by dividing the annual fixed costs by the mean annual number of admission events generating hospitalization during the most recent five-year period, thereby providing a cost estimate that more closely reflects current admission patterns at the rehabilitation center. Admission-related costs were assigned per admission event. Variable costs were calculated from the estimated frequency of diagnostic and therapeutic procedures typically required for each major cause of admission. For each procedure, the current unit cost was multiplied by its estimated frequency of use, and the resulting values were summed to obtain the estimated variable cost associated with each admission category. Variable feeding costs were estimated using the current feeding cost per kilogram of body weight and day provided by the rehabilitation center. Cost calculations were based on the mean body mass of admitted turtles and the median length of stay (P_50_) for hospitalization episodes within each cause of admission. The total estimated rehabilitation cost per released turtle was calculated as the sum of the allocated fixed cost, admission-related cost, and cause-specific variable cost. This approach provided a standardized estimate of current rehabilitation costs rather than a reconstruction of historical expenditures.

### 2.4 Statistical analysis

All statistical analyses were performed using IBM SPSS Statistics for Windows, v.29.0 (IBM Corp., Armonk, NY, United States). Descriptive statistics were calculated for all study variables. Continuous variables were summarized as mean ± standard deviation (SD), median, range, or selected percentiles (P_10_, P_25_, P_50_, P_75_, and P_90_), as appropriate. Categorical variables were summarized as absolute frequencies and percentages. Unless otherwise specified, statistical analyses were based on the first admission of each unique individual turtle.

Comparisons of the distribution of causes of admission and causes of mortality among turtles dead on first admission between loggerhead and green turtles were performed using the Fisher–Freeman–Halton exact test because of the small number of observations in several categories. When significant overall associations were detected, *post hoc* 2 × 2 comparisons were performed for each major category by comparing that category with all remaining categories combined. Odds ratios (ORs) with 95% confidence intervals (95% CIs) were calculated to quantify the direction and magnitude of the associations. Fisher’s exact test was used for individual *post hoc* comparisons when sparse or zero cell counts were present. To account for multiple *post hoc* comparisons within each family of analyses, *p*-values were adjusted using the Holm method. The strength of the overall association was assessed using Cramér’s *V*.

Associations between cause of admission and rehabilitation outcome were evaluated only for unassisted mortality during hospitalization and release, because euthanasia was an infrequent outcome and several cause-specific categories contained very small numbers or zero euthanized turtles. For the analysis of unassisted mortality during hospitalization, turtles that died unassisted during hospitalization were coded as the event, whereas euthanized and released turtles were coded as not experiencing this outcome. For the analysis of release, released turtles were coded as the event, whereas euthanized turtles and those that died unassisted during hospitalization were coded as not experiencing this outcome. Thus, euthanasia remained a distinct final disposition and was not considered equivalent to either unassisted mortality during hospitalization or successful release.

Pearson’s Chi-square (*χ*^2^) test was used to assess the overall association between cause of admission and outcome. Effect size was quantified using Cramér’s *V*. For significant overall associations, *post hoc* 2 × 2 comparisons were performed for each admission category against all remaining categories combined, and ORs with 95% CIs were calculated. *p*-values from these *post hoc* comparisons were adjusted using the Holm method.

To evaluate whether temporal changes in clinical and rehabilitation practices influenced the observed associations between cause of admission and rehabilitation outcome, additional sensitivity analyses were performed using binary logistic regression. Separate cause-specific models were fitted for unassisted mortality during hospitalization and release, with each cause-of-admission category compared with all remaining categories combined and admission period (1982–2009, 2010–2019, and 2020–2025) included as a categorical covariate. Adjusted odds ratios (aORs) with 95% confidence intervals were calculated, and *p*-values for the cause-specific comparisons were adjusted using the Holm method. Life stage was not included in these models because SCL data required for life-stage classification were unavailable for a substantial proportion of turtles and the availability of these data varied markedly across the study period.

Formal statistical comparisons of rehabilitation outcomes between species were not performed because of the limited number of green turtles admitted alive and the resulting sparse data in several cause-specific categories. Similarly, statistical comparisons of post-release readmission intervals between species were not conducted because of the small number of readmission events recorded for green turtles and the lack of complete independence among repeated observations from the same individuals.

All statistical tests were two-tailed, and differences were considered statistically significant at *p* < 0.05.

## 3 Results

### 3.1 Descriptive analyses

A total of 2,270 individual turtles were included in this study: 2,216 loggerheads (97.6%) and 54 green turtles (2.4%). Most turtles (79.9%, *n* = 1,813) were alive when first admitted (1,779 loggerheads [98.1%] and 34 green turtles [1.9%]). SCL measurements were available for 1,071 loggerheads, with a mean ± SD of 34.6 ± 12.7 cm (range, 14.5–100 cm), whereas body weight was available for 998 individuals, with a mean ± SD of 8.7 ± 11 kg (range, 0.3–97 kg). Based on SCL, 76.1% (*n* = 815) of loggerheads were classified as pelagic juveniles, 22.1% (*n* = 237) as juveniles/subadults, and only 1.8% (*n* = 19) as adults. SCL measurements were available for 28 green turtles, with a mean ± SD of 46.0 ± 12.6 cm (range, 30–76 cm), whereas body weight was available for 22 individuals, with a mean ± SD of 15.4 ± 15.1 kg (range, 3.6–56.7 kg). Based on SCL, 89.3% (*n* = 25) of green turtles were classified as pelagic juveniles and 10.7% (*n* = 3) as juveniles/subadults. BCI at admission could be calculated for 1,000 turtles with both SCL and body weight available (978 loggerheads and 22 green turtles). Median BCI was 1.6 (interquartile range [IQR], 1.4–1.7) in loggerheads and 1.4 (IQR, 1.2–1.4) in green turtles.

### 3.2 Distribution of causes of admission

The number of cases and the frequency distribution of causes at first admission in loggerhead and green turtles are summarized in Table 1, and the corresponding species-specific percentage distributions are shown in Figure 2. Additional data stratified by SCL category are provided in Supplementary Table S2. Considering the first admission of all individual turtles included in the study, regardless of species, entanglement in fishing gear and/or plastic materials was the most common cause of admission (42.8%, *n* = 971), followed by unknown/undetermined cause (21.5%, *n* = 487), and other causes (13%, *n* = 295). These three categories were also the most common among loggerheads (43.6% [*n* = 967], 21.5% [*n* = 476], and 13.2% [*n* = 293], respectively). In contrast, the most frequent causes of admission for green turtles were ingestion of fishing hooks and monofilament line (35.2%, *n* = 19), trauma (vessel collisions) (27.8%, *n* = 15), and unknown/undetermined causes (20.4%, *n* = 11). Despite the limited number of green turtles included in the first-admission dataset (*n* = 54), the distribution of causes at first admission differed significantly between species (Fisher–Freeman–Halton exact test, *p* < 0.001), with a moderate effect size (Cramér’s *V* = 0.27). *Post hoc* comparisons showed that loggerhead turtles had significantly greater odds of admission because of entanglement than green turtles (OR = 9.7; 95% CI: 3.5–26.9; adjusted *p* < 0.001), whereas green turtles had significantly greater odds of admission because of hook/monofilament ingestion (OR = 9.2; 95% CI: 5.1–16.5; adjusted *p* < 0.001) and trauma (OR = 9.9; 95% CI: 5.2–18.6; adjusted *p* < 0.001). No significant interspecific differences were detected for infectious disease, crude oil exposure, other causes, or unknown/undetermined causes after adjustment for multiple comparisons. Complete results of the *post hoc* interspecific comparisons are provided in Supplementary Table S3.

**Table 1:** Number of cases and frequency distribution of causes at first admission in loggerhead and green turtles during the study period (1982–2025).

Cause of admission	Loggerheads		Green turtles		Total turtles	
	Number	%	Number	%	Number	%
Entanglement	967	43.6	4	7.4	971	42.8
Hooks/monofilament lines	124	5.6	19	35.2	143	6.3
Trauma (collisions)	83	3.7	15	27.8	98	4.3
Infectious disease	216	9.7	3	5.6	219	9.6
Crude oil	57	2.6	0	0	57	2.5
Other causes	293	13.2	2	3.7	295	13
Plastic ingestion	3	0.1	0	0	3	0.1
Buoyancy disorder	55	2.5	0	0	55	2.4
Shark attack	8	0.4	0	0	8	0.4
Malnutrition	151	6.8	1	1.9	152	6.7
Miscellany	76	3.4	1	1.9	77	3.4
Unknown/undetermined	476	21.5	11	20.4	487	21.5
Total	2,216	97.6	54	2.4	2,270	100

**Figure 2:**
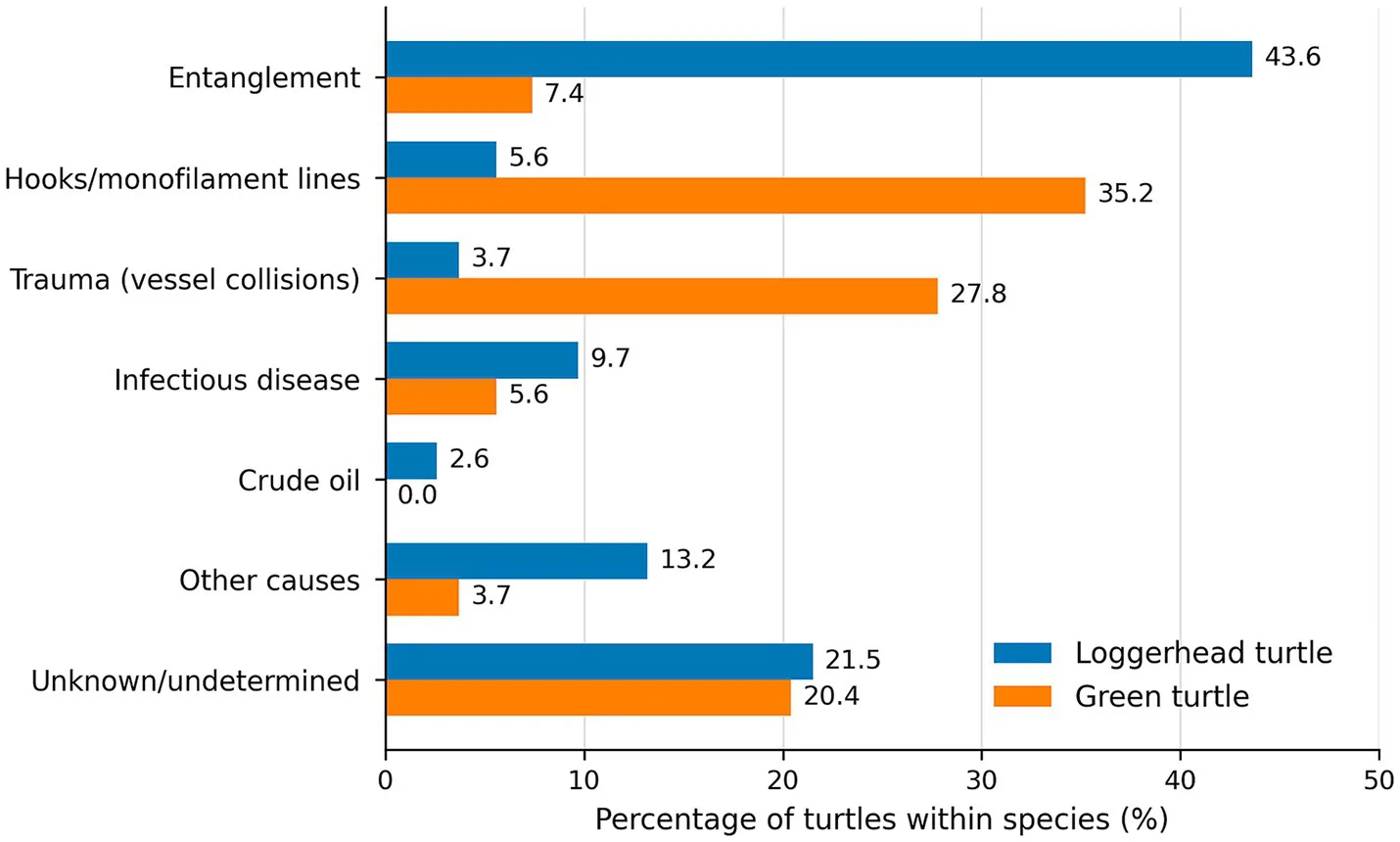
Species-specific distribution of causes at first admission in loggerhead and green turtles admitted to the La Tahonilla Wildlife Rehabilitation Center (1982–2025). Bars represent the percentage of turtles within each species assigned to each admission category. Corresponding numerical frequencies and percentages are provided in Table 1.

### 3.3 Turtles dead on admission

A total of 437 loggerheads and 20 green turtles were dead on first admission (Table 2). When all turtles dead on first admission were considered, regardless of species, unknown/undetermined causes were the most frequently identified causes of mortality (63.9%, *n* = 292), followed by entanglement (18.6%, *n* = 85), trauma (9%, *n* = 41), ingestion of hooks (3.3%, *n* = 15), and crude oil exposure (3.3%, *n* = 15). The distribution of mortality causes at first admission differed significantly between loggerhead and green turtles (Fisher–Freeman–Halton exact test, *p* = 0.005), with a moderate effect size (Cramér’s *V* = 0.24). *Post hoc* comparisons showed that green turtles had significantly greater odds of being admitted dead due to trauma than loggerhead turtles (OR = 8.2; 95% CI: 3.1–21.4; adjusted *p* < 0.001). No other significant interspecific differences were detected after adjustment for multiple comparisons. Complete results of the *post hoc* interspecific comparisons are provided in Supplementary Table S3.

**Table 2:** Causes of mortality in sea turtles dead on first admission during the study period (1982–2025).

Cause of mortality	Loggerheads		Green turtles		Total turtles	
	Number	%	Number	%	Number	%
Entanglement	83	19	2	10	85	18.6
Hooks/monofilament lines	14	3.2	1	5	15	3.3
Trauma (collisions)	33	7.6	8	40	41	9
Infectious disease	1	0.2	0	0	1	0.2
Crude oil	15	3.4	0	0	15	3.3
Other causes	8	1.8	0	0	8	1.8
Plastic ingestion	0	0	0	0	0	0
Buoyancy disorder	3	0.7	0	0	3	0.7
Shark attack	0	0	0	0	0	0
Malnutrition	3	0.7	0	0	3	0.7
Miscellany	2	0.5	0	0	2	0.4
Unknown/undetermined	283	64.8	9	45	292	63.9
Total	437	95.6	20	4.4	457	100

### 3.4 Final disposition

The final disposition of the 1,813 turtles alive at first admission showed the following rates: E_r_ = 1.4% (*n* = 26), M_r_ = 12.5% (*n* = 227), and R_r_ = 86% (*n* = 1,560). Among the 1,779 loggerheads alive at first admission, E_r_, M_r_, and R_r_ were 1.5% (*n* = 26), 12.5% (*n* = 223), and 86% (*n* = 1,530), respectively. Among the 34 green turtles alive at first admission, E_r_, M_r_, and R_r_ were 0% (*n* = 0), 11.8% (*n* = 4), and 88.2% (*n* = 30), respectively. Statistical comparisons of rehabilitation outcomes between species were not conducted because the number of green turtles alive at first admission was limited (*n* = 34), resulting in sparse data and insufficient statistical power in several cause-specific categories. The final dispositions of turtles alive at first admission stratified by cause of admission are shown in Table 3.

**Table 3:** Final disposition of turtles alive at first admission during the study period (1982–2025), stratified by cause of admission.

Cause of admission	Loggerheads							Green turtles							Total turtles						
	*n*	Final disposition						*n*	Final disposition						*n*	Final disposition					
		Euthanized		Dead		Released			Euthanized		Dead		Released			Euthanized		Dead		Released	
		*n*	E_r_ (%)	*n*	M_r_ (%)	*n*	R_r_ (%)		*n*	E_r_ (%)	*n*	M_r_ (%)	*n*	R_r_ (%)		*n*	E_r_ (%)	*n*	M_r_ (%)	*n*	R_r_ (%)
Entanglement	884	13	1.5	74	8.4	797	90.2	2	0	0	0	0	2	100	886	13	1.5	74	8.4	799	90.2
Hooks/monofilament lines	110	3	2.7	34	30.9	73	66.4	18	0	0	0	0	18	100	128	3	2.3	34	26.6	91	71.1
Trauma (collisions)	50	5	10	14	28	31	62	7	0	0	2	28.6	5	71.4	57	5	8.8	16	28.1	36	63.2
Infectious disease	215	1	0.5	52	24.2	162	75.3	3	0	0	1	33.3	2	66.7	218	1	0.5	53	24.3	164	75.2
Crude oil	42	0	0	0	0	42	100	0	—	—	—	—	—	—	42	0	0	0	0	42	100
Other causes	285	4	1.4	31	10.9	250	87.7	2	0	0	1	50	1	50	287	4	1.4	32	11.1	251	87.5
Plastic ingestion	3	0	0	1	33.3	2	66.7	0	—	—	—	—	—	—	3	0	0	1	33.3	2	66.7
Buoyancy disorder	52	0	0	2	3.8	50	96.2	0	—	—	—	—	—	—	52	0	0	2	3.8	50	96.2
Shark attack	8	2	25	1	12.5	5	62.5	0	—	—	—	—	—	—	8	2	25	1	12.5	5	62.5
Malnutrition	148	1	0.7	27	18.2	120	81.1	1	0	0	1	100	0	0	149	1	0.7	28	18.8	120	80.5
Miscellany	74	1	1.4	0	0	73	98.6	1	0	0	0	0	1	100	75	1	1.3	0	0	74	98.7
Unknown/undetermined	193	0	0	18	9.3	175	90.7	2	0	0	0	0	2	100	195	0	0	18	9.2	177	90.8
Total	1,779	26	1.5	223	12.5	1,530	86	34	0	0	4	11.8	30	88.2	1,813	26	1.4	227	12.5	1,560	86

E_r_, euthanasia rate; M_r_, unassisted mortality rate during hospitalization; R_r_, release rate.

Because euthanasia was an infrequent outcome among turtles alive at first admission (1.4%), and several cause-specific categories included zero or very few euthanized animals, separate statistical analysis of euthanasia by cause of admission was not performed.

Unassisted mortality rates during hospitalization were highest for trauma (28.1%), hook/monofilament ingestion (26.6%), and infectious disease (24.3%), whereas substantially lower unassisted mortality rates were observed for entanglement (8.4%) and unknown/undetermined causes (9.2%). The probability of unassisted death during hospitalization differed significantly among causes of admission (*χ*^2^ = 85.8, d*f* = 6, *p* < 0.001), with a moderate effect size (Cramér’s *V* = 0.22). *Post hoc* comparisons showed significantly greater odds of unassisted mortality during hospitalization for turtles admitted because of hook/monofilament ingestion (OR = 2.8; 95% CI: 1.8–4.3), trauma (OR = 2.9; 95% CI: 1.6–5.2), and infectious disease (OR = 2.6; 95% CI: 1.9–3.7), whereas turtles admitted because of entanglement had significantly lower odds of unassisted mortality (OR = 0.5; 95% CI: 0.3–0.6). No unassisted deaths during hospitalization occurred among turtles admitted because of crude oil exposure (0/42; adjusted *p* = 0.022); therefore, the corresponding OR was not estimable (Figure 3A).

**Figure 3:**
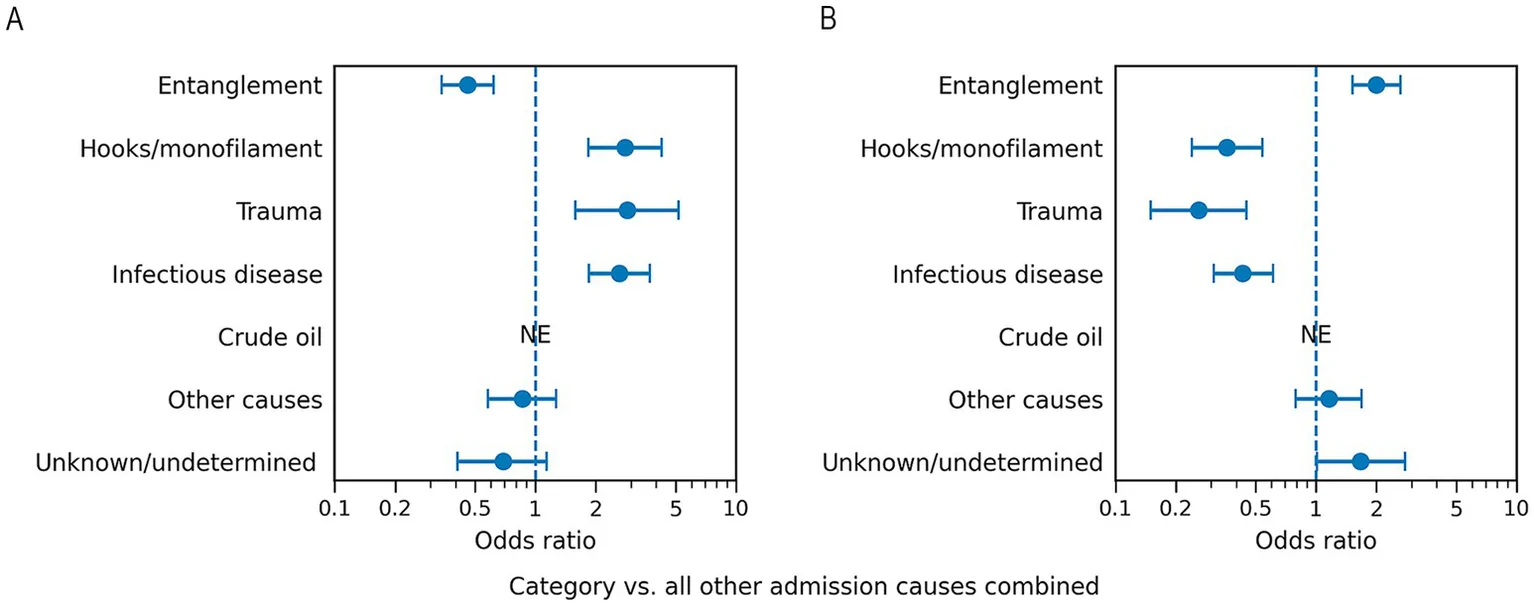
Associations between cause at first admission and rehabilitation outcomes in sea turtles admitted alive to the La Tahonilla Wildlife Rehabilitation Center (1982–2025). Forest plots show unadjusted odds ratios (ORs) and 95% confidence intervals (CIs) for **(A)** unassisted mortality during hospitalization and **(B)** release after rehabilitation. For each admission category, the OR was calculated by comparing turtles in that category with turtles in all remaining admission categories combined. The vertical dashed line indicates the null value (OR = 1). NE, not estimable because of zero cell counts.

Release rates ranged from 63.2% for trauma cases to 100% for turtles admitted because of crude oil exposure, with high release rates also observed for unknown/undetermined causes (90.8%) and entanglement (90.2%). Release outcome was significantly associated with cause of admission (*χ*^2^ = 93.5, d*f* = 6, *p* < 0.001), with a moderate effect size (Cramér’s *V* = 0.23). *Post hoc* comparisons showed significantly greater odds of release for turtles admitted because of entanglement (OR = 2.0; 95% CI: 1.5–2.6), whereas significantly lower odds of release were observed for turtles admitted because of hook/monofilament ingestion (OR = 0.4; 95% CI: 0.2–0.5), trauma (OR = 0.3; 95% CI: 0.1–0.5), and infectious disease (OR = 0.4; 95% CI: 0.3–0.6). All turtles admitted because of crude oil exposure were released (42/42; adjusted *p* = 0.008); therefore, the corresponding OR was not estimable (Figure 3B).

Adjustment for admission period produced only minor changes in the cause-specific effect estimates. The principal associations remained unchanged, with entanglement associated with lower odds of unassisted mortality during hospitalization and higher odds of release, whereas hook/monofilament ingestion, trauma, and infectious disease remained associated with higher unassisted mortality and lower release odds. Complete results of the period-adjusted sensitivity analyses are provided in Supplementary Table S4.

### 3.5 Time to final disposition

Among turtles that died unassisted during hospitalization, the median T_d_ ranged from 2 days (trauma) to 11 days (entanglement) (Table 4). Among released turtles, median rehabilitation time ranged from 10 days in crude oil cases to 37 days in trauma cases, whereas the 90th percentile reached 213 days for trauma-related admissions. Overall, among released turtles, trauma cases showed the longest observed rehabilitation periods, whereas entanglement, hook/monofilament ingestion, infectious disease, and unknown/undetermined causes showed comparatively shorter lengths of stay (Table 4). Descriptive statistics of these parameters for loggerheads and green turtles are shown in Supplementary Table S5.

**Table 4:** Descriptive statistics of time from admission to final disposition in sea turtle hospitalization episodes by cause of admission during the study period (1982–2025).

Cause of admission	Time (days) from admission to final disposition														
	Euthanasia					Unassisted mortality					Release				
	P_10_	P_25_	P_50_	P_75_	P_90_	P_10_	P_25_	P_50_	P_75_	P_90_	P_10_	P_25_	P_50_	P_75_	P_90_
Entanglement	0	1	2	30.5	88.6	1	4	11	30.7	59.5	6	12	23	43	69
Hooks/monofilament lines	0	0	1	—	—	1	1	3	9.2	28.5	4.4	11.5	21	39.5	76
Trauma (collisions)	0	0.5	5	79	—	0	1	2	12.5	81.9	4	7	37	106	213
Infectious disease	N/A	N/A	N/A	N/A	N/A	1	1.5	7	22.5	39	7	12	22	46.3	86.8
Crude oil											2.3	5	10	21.5	54.3
Other causes	0	0.2	1.5	167.7	—	0.3	3	7.5	29.7	66.5	4	8	16	31	53
Plastic ingestion						N/A	N/A	N/A	N/A	N/A	6	6	10.5	—	—
Buoyancy disorder						3	3	20	—	—	5	7.3	14	23	44.6
Shark attack	0	0	0.5	—	—	N/A	N/A	N/A	N/A	N/A	25	25	40	47	—
Malnutrition	N/A	N/A	N/A	N/A	N/A	0.9	3	7.5	29.7	68	5	13.8	24	40.3	78
Miscellany	N/A	N/A	N/A	N/A	N/A						3	5	9	15.2	40.5
Unknown/undetermined						0	1	5.5	19.5	53.3	1	5	14	26	63.3

P_10_, P_25_, P_50_, P_75_, P_90_: 10th, 25th, 50th (median), 75th and 90th percentiles; N/A, not applicable because only one observation was available; —, percentile could not be estimated because of the limited number of observations. Hospitalization episodes include first admissions and subsequent readmissions of turtles admitted alive. Species-specific descriptive statistics for loggerhead and green turtles are provided in Supplementary Table S5.

### 3.6 Post-release readmissions and outcomes

Of the 1,560 turtles released following their first admission, only 16 individuals (13 loggerheads and 3 green turtles) were subsequently readmitted, representing 1.0% of all turtles released following their first admission (Table 5). Thirteen turtles experienced a single readmission, whereas one turtle each underwent two, three, and four readmissions, respectively, resulting in a total of 22 readmission events. At their first readmission, two turtles (12.5%) were found dead, whereas the remaining 14 turtles (87.5%) were admitted alive. The most common causes among all readmission events were buoyancy disorder and infectious disease, each accounting for 27.3% (*n* = 6) of readmission events. The most common cause at first admission among the 16 turtles that were subsequently readmitted was ingestion of hooks/monofilament lines (31.2%, *n* = 5). Furthermore, most readmission events (63.6%, *n* = 14) were attributed to a cause different from that of the immediately preceding admission or readmission.

**Table 5:** Distribution of post-release readmission events by cause and readmission order in sea turtles during the study period (1982–2025).

Cause of readmission	First readmission	Second readmission	Third readmission	Fourth readmission
Entanglement				
Hooks/monofilament lines	1	1		
Trauma (collisions)	4^a^			
Infectious disease	5		1	
Crude oil				
Other causes	4	2	1	1
Plastic ingestion				
Buoyancy disorder	3	1	1	1
Shark attack				
Malnutrition	1	1		
Miscellany				
Unknown/undetermined	2^a^			
Total readmission events (*n* = 22)	16	3	2	1

a One turtle in this readmission-cause category was found dead at the time of readmission.

The interval between release and subsequent readmission showed considerable variability across the 22 readmission events, ranging from 0 to 1,425 days. Overall, the distribution was highly right-skewed, with a median interval of 24.5 days but a small number of turtles being readmitted several years after release (Table 6). Time intervals between release and subsequent readmissions appeared substantially longer in green turtles (median: 1,047 days; range: 862–1,425 days; mean ± SD: 1095.3 ± 265.6 days; *n* = 4 intervals) than in loggerhead turtles (median: 10 days; range: 0–721 days; mean ± SD: 98.7 ± 187.9 days; *n* = 18 intervals). However, formal statistical comparisons between species were not performed because of the small number of readmission intervals recorded for green turtles and the lack of complete independence among some repeated observations.

**Table 6:** Descriptive statistics of time intervals between release and subsequent readmissions in rehabilitated sea turtles during the study period (1982–2025).

Readmission event	*n*	Range (days)	Median (days)	Mean ± SD (days)
First readmission	16	0–1,425	24.5	294.7 ± 479.7
Second readmission	3	10–862	424	432 ± 426.1
Third readmission	2	1–143	72	72 ± 100.4
Fourth readmission	1	2-2	N/A	N/A
Overall	22	0–1,425	24.5	279.9 ± 439.8

N/A, not applicable because only one observation was available.

All 20 readmission events involving turtles admitted alive resulted in successful rehabilitation and release, corresponding to a post-readmission R_r_ of 100%.

### 3.7 Rehabilitation costs

The estimated rehabilitation costs per rehabilitated turtle according to cause of admission based on current costs are summarized in Table 7. Fixed and admission-related costs were identical for all admission categories because they were estimated using standardized assumptions. The estimated standardized feeding cost was 1.84 € per rehabilitated turtle per day. Differences in the estimated rehabilitation costs among admission categories were driven principally by the cost of diagnostic and therapeutic procedures, whereas variation in feeding costs was comparatively small despite differences in rehabilitation duration.

**Table 7:** Estimated rehabilitation cost per rehabilitated turtle according to cause of admission based on current costs.

Cause of admission	Fixed costs^a^ (€)	Admission-related costs (€)	Variable costs^b^ (€)		Total (€)
			Feeding costs (1.84 €/day)	Diagnostic and therapeutic procedures	
Entanglement	974	250	42.3	466	1732.3
Hooks/monofilament lines	974	250	38.6	866	2128.6
Trauma (collisions)	974	250	68.1	1,066	2358.1
Infectious disease	974	250	40.5	641	1905.5
Crude oil	974	250	18.4	256	1498.4
Other causes	974	250	29.4	381	1634.4
Unknown/undetermined	974	250	25.8	656	1905.8

a Calculated by dividing the annual fixed costs (64,480 €) by the mean annual number of admission events generating hospitalization (66.2 events/year) during the most recent five-year period (2021–2025).

b Variable costs were estimated using the median length of stay (P_50_) for hospitalization episodes within each cause of admission together with the estimated frequency of diagnostic and therapeutic procedures.

An important finding was that fixed costs represented the largest cost component in most admission categories, accounting for between 41.3 and 65% of the total estimated rehabilitation cost per turtle depending on the cause of admission. The relative contribution of fixed costs was greatest in admission categories with lower overall rehabilitation costs and decreased progressively as expenditures on diagnostic and therapeutic procedures increased.

## 4 Discussion

### 4.1 Descriptive characteristics of the study population

At first admission, most loggerheads admitted to the LTWRC were pelagic juveniles, and pelagic juveniles also predominated among the considerably smaller sample of green turtles. SCL measurements were unavailable for 51.6% of the turtles included in the study, which limited the assessment of potential life-stage-related differences. Body weight at admission was available for fewer individuals, and both SCL and body weight were available for 1,000 turtles, limiting calculation of BCI to this subset. Moreover, the availability of morphometric data varied markedly across the study period, reflecting less systematic recording during earlier decades; consequently, analyses restricted to turtles with complete morphometric data may not be representative of the full historical cohort.

The BCI values recorded at admission provide additional information on the body condition of the turtles included in the study. The median BCI observed in loggerheads (1.6) was comparable with values previously reported for free-ranging oceanic juvenile loggerheads from the North Atlantic (21). Similarly, the median BCI recorded in green turtles (1.4) was comparable with values reported for free-ranging green turtles in Florida that were subjectively classified as being in good or robust body condition (22). These comparisons suggest that, at the population level, the turtles included in our first-admission dataset did not generally exhibit markedly poor body condition. Nevertheless, BCI should be interpreted cautiously because values may vary according to species, body size, population, and physiological or clinical status, and universally applicable reference ranges have not been established for sea turtles. Moreover, morphometric indices based on body mass and carapace length provide only an indirect estimate of body condition and have limited ability to predict actual adipose tissue reserves (23). In the present study, BCI was calculated from measurements obtained at admission and therefore reflects body condition at that time rather than changes occurring during rehabilitation.

### 4.2 Causes of admission

The contribution of sea turtle rehabilitation centers to the identification of major stranding causes represents a clear example of how veterinary medicine can support wildlife conservation strategies and inform policy development, particularly in efforts to mitigate anthropogenic threats (6, 11, 13). In the northeast (NE) Atlantic region, studies assessing the causes of sea turtle strandings and mortality over periods longer than 10 years remain scarce (6, 24–26). This retrospective study spanned 43 years and 8 months and included the largest sample of turtles examined in any study conducted in the NE Atlantic region.

Entanglement associated with fishing gear and plastic debris (including synthetic raffia) emerged as the most common cause at first admission (42.8%) in our study. Numerous studies have identified entanglement as an important contributor to sea turtle mortality worldwide (6, 15, 27–31). Entanglement prevalence in our study was greater than that described in most previous studies worldwide (27–29) but remained below the levels reported for Gran Canaria Island (50.8%) (6), southeastern Brazil (53%) (30), and the Maldives (66.2%) (15). Sea turtle interactions with the artisanal fisheries operating in the Canary Islands are considered uncommon; nevertheless, loggerhead turtles may occasionally become involved with fishing activities, especially where trasmallos (multi-panel gillnets) are deployed (32). Accurately identifying the source of entanglement is frequently problematic because animals can become trapped in both operational fishing gear and derelict fishing equipment present in the marine environment (33). Interactions with human-derived plastic debris, including abandoned fishing equipment and plastics originating from terrestrial environments, represent a poorly documented and insufficiently investigated threat to sea turtle populations (33). Expert evaluations ranked entanglement in derelict fishing gear among the most important anthropogenic threats affecting sea turtles, surpassing oil pollution, climate change, and direct exploitation, but being considered less severe than fisheries bycatch and plastic ingestion (33). A relevant factor to consider is the widespread use of synthetic raffia in the Canary Islands, especially on islands with highly developed intensive farming systems (6). Our findings corroborate earlier recommendations advocating legal requirements for the use of biodegradable raffia, supporting its implementation as a measure to lessen the impact of raffia-related sea turtle strandings in the Canary Islands (6).

In the present study, admissions attributed to unknown/undetermined causes represented the second most frequent category (21.5%). This prevalence was lower than that reported in most studies of sea turtle stranding causes (11, 34–40), where the proportion of cases assigned to the “unknown/undetermined” category exceeds 60% in some instances (36–40). The identification of a definitive stranding cause is frequently hindered by resource and time constraints that limit diagnostic workups, the rapid onset of postmortem decomposition that may preclude comprehensive pathological and histological assessments, and the inability to reach a conclusive diagnosis even after extensive investigative efforts (6, 28). In addition, the study period extended from 1982 to 2025, during which substantial advances occurred in diagnostic imaging, microbiology, molecular diagnostics, and clinical pathology. Consequently, some turtles admitted during the earlier years of the study may have been classified as having unknown/undetermined causes of admission because the diagnostic tools currently available to the LTWRC were not yet accessible at that time. Although the retrospective review and assignment of cases to predefined admission categories helped standardize classification across the study period, formal inter-rater agreement among clinicians was not assessed and therefore some degree of classification variability cannot be excluded.

The “other causes” category ranked third among the causes at first admission (13%), with malnutrition being particularly prominent (6.7%). In a similar study conducted in the same region (Gran Canaria Island), only 3.4% of admissions were assigned to the “other causes” category. However, comparisons should be interpreted with caution, as that study covered a substantially shorter period (17 years) and involved a smaller number of turtles (6).

Infectious disease ranked fourth among the causes at first admission in our study, accounting for 9.6% of cases. This percentage is nearly twice that reported in a study of strandings on the neighboring island of Gran Canaria (5.5%) (6). Because cases were classified as infectious disease only when infection by a pathogenic microorganism was confirmed through microbiological or histopathological analyses, the observed prevalence of this category reflects the LTWRC’s commitment to achieving the highest possible diagnostic accuracy regarding the cause of admission. In addition, consistent with previous reports from the Canary Islands (5, 6), no cases of fibropapillomatosis were identified among stranded turtles in the present study, although the disease is widely distributed in sea turtle populations worldwide (41).

In our study, the distribution of causes at first admission differed significantly between loggerhead and green turtles. Loggerheads were substantially more likely to be admitted because of entanglement, whereas green turtles had significantly greater odds of admission because of hook/monofilament ingestion and trauma. However, the number of green turtles included in the study was considerably smaller than that of loggerheads, and therefore, interspecific comparisons should be interpreted with caution. The small green turtle sample may have reduced statistical precision, particularly for less frequent admission categories, and may have resulted in less stable odds ratio estimates and wider confidence intervals. Nevertheless, the moderate effect size observed (Cramér’s *V* = 0.27) and the consistency of the patterns identified suggest that the observed interspecific differences are unlikely to be attributable solely to sampling variation. Most loggerheads admitted to the LTWRC were pelagic juveniles, a life stage that spends prolonged periods in oceanic waters where floating plastics and abandoned fishing gear are abundant. In contrast, green turtles inhabiting the Canary Islands are typically associated with more coastal habitats, increasing their exposure to artisanal fisheries, recreational fishing activities, and vessel traffic. Consequently, the higher frequency of hook ingestion and collision-related injuries observed in green turtles appears biologically plausible.

### 4.3 Causes of mortality in turtles dead on first admission

Unknown/undetermined causes represented by far the most common category among turtles dead on first admission, accounting for 63.9% of these cases. This proportion was substantially higher than that observed among turtles alive at first admission. Several factors may explain this finding. Many carcasses were likely recovered after variable periods of postmortem decomposition, which can obscure diagnostic lesions and hinder the identification of the primary cause of death. In addition, some mortality events may have resulted from multifactorial processes or from conditions that left limited gross pathological evidence. As discussed for live-admitted turtles, the retrospective nature of the study and the considerable advances in diagnostic capabilities over the four decades encompassed by the survey may also have contributed to the classification of some historical cases as unknown/undetermined.

Entanglement was the second most common cause of mortality among turtles dead on first admission, accounting for 18.6% of these cases. This finding reinforces the importance of abandoned fishing gear and plastic materials as major threats to sea turtles in the Canary Islands (6). Not only was entanglement the leading cause of admission among turtles alive at first admission, but it also represented a substantial proportion of mortality cases, indicating that interactions with marine debris may result in both sub-lethal and fatal outcomes. The persistence of this threat throughout the study period highlights the need for continued efforts to reduce marine litter and discarded fishing materials in the region.

The significantly greater likelihood of green turtles being dead on first admission due to trauma suggests important interspecific differences in exposure to anthropogenic threats. Green turtles inhabiting the Canary Islands typically use coastal habitats more intensively than loggerheads, increasing their probability of interacting with motorized vessels and other human activities concentrated in nearshore environments. This interpretation is supported by the significantly greater odds of admission because of trauma observed in green turtles. Taken together, these results indicate that vessel-related injuries may represent a particularly important conservation concern for green turtles in the archipelago. However, because several mortality categories contained sparse or zero cell counts, the corresponding effect-size estimates and category-specific comparisons should be interpreted cautiously.

Overall, the mortality patterns observed in turtles admitted dead suggest that anthropogenic factors, particularly marine debris and vessel-related trauma, continue to play a major role in sea turtle mortality in the Canary Islands. These findings emphasize the importance of preventive conservation measures aimed at reducing human-induced threats before rehabilitation becomes necessary.

### 4.4 Rehabilitation outcomes

Wildlife rehabilitation is the practice of caring for sick, injured, or orphaned wild animals with the goal of releasing them back to the wild (42). Within the broader field of wildlife rehabilitation, programs dedicated to sea turtles comprise a distinct and specialized area of practice (43). While welfare-based rehabilitation protocols and pre-release health screening guidelines for sea turtles have been reported (44–46), no validated benchmarks are currently available to evaluate the quality of rehabilitation efforts for injured individuals. In this study, the quality of the sea turtle rehabilitation program at the LTWRC was assessed using the benchmark indicators E_r_, M_r_, and R_r_, which were evaluated according to the primary cause of admission.

Analysis of the rehabilitation outcomes showed that 86% of sea turtles alive at first admission to the LTWRC were successfully returned to the wild, while 14% either died unassisted during hospitalization or were euthanized. This release rate is comparable only to those reported from the neighboring island of Gran Canaria (86.3%) (6) and exceed those documented in other geographic regions (8, 11, 14, 15, 47–50). By contrast, comparable studies involving several thousand sea turtles have reported release rates below 40%; among 1,700 sea turtles admitted to rehabilitation facilities in Florida (USA) between 1986 and 2004, 36.8% were successfully rehabilitated and released (8); between 1996 and 2013, rehabilitation facilities in Queensland (Australia) successfully released 39% of the 2,970 sea turtles that were admitted for treatment (11). However, in similar studies involving only a few hundred sea turtles, release rates varied widely, ranging from 19.3 to 71.2% (14, 15, 47–50). Our findings support the value of the LTWRC as an effective tool for the recovery of stranded sea turtles and provide further evidence that wildlife rehabilitation can contribute substantially to the conservation of affected individuals.

The present study also demonstrated that rehabilitation outcome was strongly influenced by the primary cause of admission. Unassisted mortality during hospitalization and release rates differed significantly among admission categories, indicating that the initial clinical condition of stranded turtles is a major determinant of prognosis. This finding highlights the importance of considering cause-specific outcomes when evaluating rehabilitation performance, as overall release rates alone may mask substantial differences among clinical conditions. Importantly, these associations remained largely unchanged after adjustment for admission period, suggesting that the observed cause-specific differences in rehabilitation outcomes were not primarily driven by temporal changes in clinical and rehabilitation practices.

Turtles admitted because of entanglement exhibited particularly favorable outcomes, with high release rates and significantly reduced odds of unassisted mortality during hospitalization. Entanglement in fishing gear and plastic debris may cause severe soft-tissue injuries, including chronic ulcerative skin lesions and loss of one or more flippers (5). Nevertheless, sea turtles generally show a remarkable capacity to adapt to limb loss, and successful rehabilitation and release have been reported following both forelimb and hindlimb amputations (6). Evidence supporting the long-term viability of amputated sea turtles was provided by a recent study from Cabo Verde, where reproductive performance was evaluated in loggerhead turtles with flipper amputations. The authors found that amputated individuals successfully reproduced, suggesting that turtles released from rehabilitation centers after limb loss may continue to contribute to population recruitment and conservation (51). In many cases, the injuries associated with entanglement may be reversible if the constricting material is removed and appropriate supportive care is provided, allowing relatively rapid recovery. This favorable prognosis may help explain why, despite being the most common cause at first admission, entanglement was associated with significantly lower odds of unassisted mortality and a higher probability of successful release than several other admission categories.

In contrast, turtles admitted because of trauma, hook/monofilament ingestion, and infectious disease showed significantly higher unassisted mortality rates and lower probabilities of successful release. These conditions frequently involve severe tissue damage, systemic compromise, prolonged treatment, or the need for complex medical and surgical interventions (6). Vessel-strike injuries may cause extensive skeletal and soft-tissue trauma (7, 52), whereas hook ingestion can result in gastrointestinal perforation, chronic inflammation, or secondary infections (4, 5). Likewise, infectious diseases often reflect advanced underlying pathology and may require prolonged and intensive treatment (6, 53). Consequently, these categories represent some of the most challenging clinical scenarios encountered in sea turtle rehabilitation.

Because the primary objective of wildlife rehabilitation is the return of animals to the wild, euthanasia is generally reserved for individuals with poor welfare prospects or an unfavorable prognosis for post-release survival (54). The overall euthanasia rate was low (1.4%), suggesting that most turtles alive at first admission retained a reasonable prognosis for rehabilitation and eventual release. This finding may reflect both the nature of the cases received and the clinical capacity of the center to manage a wide range of medical and traumatic conditions. Among the main admission categories, the highest E_r_ was observed in turtles admitted because of vessel-related trauma. Boat strikes frequently cause extensive fractures of the carapace and plastron together with severe damage to underlying organs, particularly the lungs and kidneys (5). As these injuries may substantially compromise survival prospects and post-release quality of life, euthanasia may represent the most appropriate clinical option in the most severely affected individuals (6).

Collectively, the outcome measures reported in this study, including E_r_, M_r_, and R_r_, calculated for turtles alive at first admission, provide objective benchmarks for evaluating rehabilitation performance in sea turtle rehabilitation facilities. Because these indicators were calculated from one of the largest and longest-running sea turtle rehabilitation datasets available worldwide, they may serve as useful reference values for quality assessment and inter-center comparisons.

### 4.5 Time to final disposition

Rehabilitation periods should be minimized whenever possible, as prolonged captivity may increase the likelihood of husbandry-related complications, infectious processes, and abnormal behavioral responses (55). The duration of rehabilitation in our study varied substantially according to the primary cause of admission, indicating marked differences in clinical severity and recovery requirements among admission categories. Trauma cases required the longest rehabilitation periods, with a median length of stay of 37 days and a P_90_ of 213 days among released turtles. This finding is consistent with the complex nature of vessel-strike injuries, which frequently involve extensive carapace fractures, soft-tissue damage, and prolonged healing processes (5, 52). Recovery from these injuries often requires long-term wound management, repeated clinical evaluations, and extended periods of supportive care before release can be considered.

In contrast, turtles admitted because of entanglement, hook/monofilament ingestion, infectious disease, and unknown/undetermined causes generally showed shorter rehabilitation periods. In many entanglement cases, removal of the constricting material combined with supportive treatment may result in relatively rapid clinical improvement, particularly when severe systemic complications have not yet developed (15, 44). Similarly, some turtles admitted for unknown/undetermined causes may have suffered from transient conditions that resolved during rehabilitation, thereby contributing to the comparatively short lengths of stay observed in these categories.

An additional finding of practical importance was the marked right-skewness of rehabilitation duration distributions, particularly among trauma cases. Although most turtles completed rehabilitation within a relatively short period, a small proportion required markedly prolonged hospitalization, especially those admitted because of trauma. Consequently, median values may provide a more representative measure of the typical rehabilitation course than arithmetic means, which can be disproportionately influenced by a limited number of long-term cases. These findings have important implications for rehabilitation planning and resource allocation, as a relatively small number of severely affected turtles may account for a substantial proportion of total hospitalization time and associated rehabilitation costs.

Species-specific descriptive statistics presented in Supplementary Table S5 should be interpreted cautiously because of the limited number of hospitalization episodes involving green turtles. Consequently, percentile estimates could not be calculated for several cause-specific categories, and formal comparisons between species were not performed. Nevertheless, the available data suggest that the overall temporal patterns observed in the combined analysis were largely driven by loggerhead turtles, which represented most admissions. Additional studies including larger numbers of green turtles would be necessary to determine whether species-specific differences in rehabilitation duration truly exist. The time-to-disposition indicators reported in this study were intended as descriptive benchmarks of rehabilitation duration according to final outcome and cause of admission, rather than as adjusted estimates of the independent effects of individual covariates on time to disposition. Because release, unassisted death, and euthanasia represent mutually exclusive competing outcomes, conventional Kaplan–Meier analyses of each disposition would require treating the alternative outcomes as censored observations and would not directly estimate their observed cumulative incidence. Future studies specifically aimed at identifying predictors of time to disposition could therefore apply competing-risks or multistate approaches, while accounting for potential confounding factors and temporal changes in clinical and rehabilitation practices.

The percentile distributions reported in the present study may provide useful benchmark values for rehabilitation centers, allowing clinicians and managers to compare the duration of treatment in their own facilities with those observed in a large long-term dataset. Such benchmarks may facilitate the identification of unusually prolonged rehabilitation periods and contribute to the evaluation of rehabilitation efficiency.

### 4.6 Post-release readmissions and outcomes

From a conservation perspective, rehabilitation can only be considered fully successful when released turtles can return to the wild population and ultimately participate in reproduction (8). Despite the large number of sea turtles released annually from rehabilitation centers, information regarding their long-term post-release survival remains limited. Comprehensive monitoring through satellite telemetry is rarely feasible because of its high cost, and therefore much of the existing evidence concerning post-release performance relies on occasional recaptures, restrandings, or readmissions of previously rehabilitated turtles identified with flipper tags or PIT tags (8, 11, 13). In our study, the proportion of turtles released following their first admission that were subsequently readmitted was remarkably low, representing only 1.0% of these individuals. In the largest sea turtle rehabilitation study conducted to date in the United States, first readmissions were documented in 2.8% of the 11,417 turtles released following rehabilitation (13), a proportion higher than that observed in the present study. An even higher readmission rate (8.6%) was reported among 1,173 rehabilitated and released turtles in Queensland, Australia (11). The interpretation of post-release outcomes should be approached with caution because several factors may limit the detection of previously released turtles, including biological characteristics of the species, constraints associated with identification and tracking methods, and the lack of complete integration among stranding and rehabilitation databases (13). Consequently, the limited number of studies that have examined this issue have been careful not to draw overly definitive conclusions (11, 13).

An additional noteworthy finding was that most readmission events were associated with a cause different from that recorded during the immediately preceding admission or readmission. This pattern suggests that many readmissions did not represent recurrence or persistence of the original condition but rather reflected new health problems or independent stranding events occurring after release. Consequently, the low frequency of readmissions combined with the predominance of different admission causes is consistent with favorable post-release outcomes following rehabilitation.

The wide variation observed in the interval between release and readmission further illustrates the complexity of interpreting post-release outcomes. Although the median interval was only 24.5 days, some turtles were not readmitted until several years after release. These long intervals make it unlikely that all readmission events were directly related to the original admission condition and instead suggest that many turtles survived for extended periods in the wild before experiencing a subsequent and unrelated stranding event. This interpretation is consistent with the observation that most readmissions were attributed to causes different from those recorded previously.

The longer intervals observed between release and readmission in green turtles may reflect species-specific differences in habitat use and movement patterns. In the Canary Islands, juvenile loggerheads are primarily oceanic and highly mobile, whereas green turtles often behave as temporary residents associated with coastal foraging habitats (3). Such differences could potentially increase the probability of detecting previously rehabilitated green turtles within the archipelago long after release. Nevertheless, the small number of observations available precludes any firm conclusions.

All 20 readmission events involving turtles admitted alive resulted in successful rehabilitation and release. Although based on a limited number of readmission events, this finding suggests that prior rehabilitation did not compromise the capacity of these animals to respond positively to subsequent treatment. It also indicates that readmitted turtles generally retained sufficient biological resilience to recover from additional health challenges and return successfully to the wild.

Taken together, the low frequency of readmissions, the predominance of new rather than recurrent causes of admission, and the 100% release rate among readmission events involving turtles admitted alive provide encouraging evidence regarding the long-term effectiveness of sea turtle rehabilitation and release programs.

### 4.7 Rehabilitation costs

Wildlife rehabilitation programs require substantial financial resources, yet relatively few studies have attempted to quantify the economic costs associated with sea turtle rehabilitation (11). Estimating rehabilitation costs according to the primary cause of admission provides useful information for both conservation planning and resource allocation, allowing rehabilitation centers to better anticipate the economic demands associated with different clinical conditions.

The present study showed that differences in rehabilitation costs among admission categories were determined principally by the diagnostic and therapeutic procedures required. Although prolonged hospitalization inevitably increased feeding expenses, this component represented only a small proportion of the overall rehabilitation cost. In contrast, diagnostic and therapeutic costs were influenced both by the severity of the presenting condition and by the length of rehabilitation, since turtles requiring prolonged hospitalization often underwent repeated clinical assessments, additional diagnostic investigations, and multiple therapeutic interventions.

One of the most relevant findings of the present cost analysis was the substantial contribution of fixed costs to the overall rehabilitation cost per turtle. These costs are incurred regardless of the number of turtles admitted and therefore represent an unavoidable operational expense for rehabilitation centers. Consequently, increasing the annual number of admissions would reduce the fixed cost allocated to each rehabilitated turtle, thereby improving cost-efficiency in terms of fixed-cost allocation. This finding highlights the importance of considering admission volume when comparing rehabilitation costs among centers, since facilities operating below their capacity may appear less cost-effective despite providing similar standards of veterinary care.

An additional aspect that should be considered when interpreting these cost estimates is that the LTWRC is a multidisciplinary wildlife rehabilitation center that provides veterinary care for a wide range of free-ranging species. Consequently, for shared resources such as veterinary personnel, only the proportion of personnel costs attributable to sea turtle rehabilitation was included in the estimation of annual fixed costs. This allocation was based on the proportion of sea turtles within the total annual wildlife caseload (approximately 5.3%), thereby avoiding overestimation of the economic burden associated with sea turtle rehabilitation. Although this proportion may vary over time according to the annual caseload, this approach provides a more realistic estimate of the costs attributable to sea turtle rehabilitation in a multispecies wildlife rehabilitation facility.

The cost estimates presented in this study should not be interpreted as universal values applicable to all rehabilitation centers, since operating expenses, personnel costs, and clinical protocols vary among institutions and countries. Nevertheless, the analytical framework proposed here may provide a useful benchmark for future economic evaluations of sea turtle rehabilitation programs and facilitate comparisons among rehabilitation centers using standardized cost categories.

Although the benchmark indicators proposed in the present study provide an objective framework for evaluating the clinical, biological, and economic performance of sea turtle rehabilitation programs, they do not capture all the contributions made by wildlife rehabilitation centers. These facilities also play important roles in environmental education, public engagement, professional training, disease surveillance, and the generation of scientific knowledge (11, 13). Although these outcomes are considerably more difficult to quantify than rehabilitation success, they represent an important component of the overall conservation value of rehabilitation centers (10). By increasing public awareness of the anthropogenic threats affecting sea turtles and promoting responsible environmental behavior, rehabilitation centers may contribute to conservation well beyond the successful treatment and release of individual animals. Future research at the LTWRC will aim to develop and validate objective indicators capable of quantifying the educational and societal impact of its environmental education activities, thereby complementing the clinical, biological, and economic benchmark indicators proposed in the present study.

## 5 Conclusion

This study provides one of the largest and longest-term evaluations of sea turtle rehabilitation outcomes conducted to date and the most comprehensive assessment available for the NE Atlantic region. The benchmark indicators proposed here, including cause-specific rehabilitation outcomes (E_r_, M_r_, and R_r_), time to final disposition, post-release readmissions, and standardized rehabilitation costs, offer an objective framework for evaluating the quality and effectiveness of sea turtle rehabilitation programs. The high release rate achieved, together with the low frequency of post-release readmissions and the successful outcomes of readmitted turtles, provides encouraging evidence that rehabilitation can make a meaningful contribution to the conservation of stranded sea turtles. Furthermore, the marked influence of the primary cause of admission on prognosis, rehabilitation duration, and economic cost highlights the importance of incorporating cause-specific analyses into assessments of rehabilitation performance. Beyond their value for clinical decision-making, these findings demonstrate how veterinary medicine can generate robust epidemiological and economic evidence to support wildlife conservation, optimize rehabilitation strategies, and inform management decisions and conservation policies aimed at mitigating anthropogenic threats to threatened marine species. Collectively, the benchmark indicators presented here provide practical tools for quality assessment and standardized inter-center comparisons while illustrating how veterinary science can bridge individual animal health, conservation management, and evidence-based policy development.

## Data Availability

The original contributions presented in the study are included in the article/Supplementary material, further inquiries can be directed to the corresponding author.
