# Supplementary material for: Benchmark indicators for evaluating sea turtle rehabilitation outcomes at a wildlife rehabilitation center on Tenerife island, Spain (1982–2025)
Source: Front Vet Sci. 2026 Oct 19;13:1948954. doi: 10.3389/fvets.2026.1948954 (PMC13726524; doi:10.3389/fvets.2026.1948954)
Supplement: Supplementary file 1 [file Data_Sheet_1.pdf]

Supplementary Data Sheet. Individual turtle records and readmission events

Section 1. First admissions of 2,270 unique individual sea turtles. Each row represents one individual turtle and its first admission during the study period. Shaded Record IDs identify the 16 turtles with one or more subsequent readmission events; their readmissions are detailed in Section 2.

Caretta caretta

Record ID	Species	Year	Admission date	Departure date	SCL (cm)	Body weight (kg)	Cause of admission	Other-cause subcategory	Final disposition
1	Caretta caretta	1982	1982-11-04	1982-11-05			other causes	Miscellany	Released
2	Caretta caretta	1982	1982-11-17	1982-11-17			other causes	Miscellany	Released
3	Caretta caretta	1983	1983-08-01	1983-12-10	31	6.4	Hooks/monofilament lines		Released
4	Caretta caretta	1986	1986-10-01	1986-10-02			other causes	Malnutrition	Released
5	Caretta caretta	1989	1989-11-14	1989-11-15	31	6.4	other causes	Miscellany	Released
6	Caretta caretta	1990	1990-04-18	1990-04-29	31	6.4	Unknown/undetermined		Died during hospitalization
7	Caretta caretta	1998	1998-01-21	1998-01-26			Unknown/undetermined		Released
8	Caretta caretta	1999	1999-05-02	1999-05-03			other causes	Miscellany	Released
9	Caretta caretta	1999	1999-05-06	1999-05-07			Infectious disease		Released
10	Caretta caretta	1999	1999-05-22	1999-05-27			trauma		Euthanized
11	Caretta caretta	1999	1999-05-23	1999-05-24			trauma		Euthanized
12	Caretta caretta	1999	1999-05-27	1999-05-28			Hooks/monofilament lines		Released
13	Caretta caretta	1999	1999-05-27	1999-05-28			Entanglement		Released
14	Caretta caretta	1999	1999-05-30	1999-07-13			Entanglement		Released
15	Caretta caretta	1999	1999-06-19	1999-06-24			Hooks/monofilament lines		Died during hospitalization
16	Caretta caretta	1999	1999-07-20	1999-07-22			other causes	Miscellany	Euthanized
17	Caretta caretta	1999	1999-08-01	1999-08-10			other causes	Miscellany	Released
18	Caretta caretta	1999	1999-08-11	1999-08-17			Unknown/undetermined		Released
19	Caretta caretta	1999	1999-08-12	1999-08-13			Unknown/undetermined		Died during hospitalization
20	Caretta caretta	1999	1999-08-13	1999-08-16			Unknown/undetermined		Released
21	Caretta caretta	1999	1999-08-29	1999-08-31			Crude oil		Released
22	Caretta caretta	1999	1999-09-02	1999-09-02			Entanglement		Released
23	Caretta caretta	1999	1999-10-10	1999-10-13			Crude oil		Released
24	Caretta caretta	1999	1999-10-15	1999-10-16			Crude oil		Released
25	Caretta caretta	2000	2000-01-03	2000-02-17			Unknown/undetermined		Released
26	Caretta caretta	2000	2000-01-21	2000-02-17			Unknown/undetermined		Released
27	Caretta caretta	2000	2000-02-08	2000-02-21			Unknown/undetermined		Released
28	Caretta caretta	2000	2000-03-31	2000-03-31	31	6.4	Unknown/undetermined		Released
29	Caretta caretta	2000	2000-04-04	2000-04-14			Infectious disease		Released
30	Caretta caretta	2000	2000-04-10	2000-04-15			Entanglement		Released
31	Caretta caretta	2000	2000-04-12	2000-04-27			Entanglement		Released
32	Caretta caretta	2000	2000-04-13	2000-04-17			Unknown/undetermined		Released
33	Caretta caretta	2000	2000-04-24	2000-04-27			Infectious disease		Released
34	Caretta caretta	2000	2000-04-24	2000-05-02			Entanglement		Released
35	Caretta caretta	2000	2000-04-24	2000-05-02			Entanglement		Released
36	Caretta caretta	2000	2000-05-11	2000-05-26			Entanglement		Released
37	Caretta caretta	2000	2000-05-13	2000-06-08			Unknown/undetermined		Released
38	Caretta caretta	2000	2000-06-09	2000-06-09			Unknown/undetermined		Died during hospitalization
39	Caretta caretta	2000	2000-06-20	2000-06-21			Unknown/undetermined		Died during hospitalization
40	Caretta caretta	2000	2000-06-30	2000-07-03			Entanglement		Died during hospitalization
41	Caretta caretta	2000	2000-07-03	2000-07-05			Unknown/undetermined		Died during hospitalization
42	Caretta caretta	2000	2000-07-04	2000-07-13			Unknown/undetermined		Released
43	Caretta caretta	2000	2000-07-04	2000-07-13			Unknown/undetermined		Released
44	Caretta caretta	2000	2000-07-13	2000-07-13			Unknown/undetermined		Admitted dead
45	Caretta caretta	2000	2000-07-21	2000-10-07			Unknown/undetermined		Released
46	Caretta caretta	2000	2000-07-23	2000-07-24			Entanglement		Released
47	Caretta caretta	2000	2000-07-24				Unknown/undetermined		Admitted dead
48	Caretta caretta	2000	2000-07-25	2000-07-27			Hooks/monofilament lines		Died during hospitalization
49	Caretta caretta	2000	2000-07-26	2000-07-27			other causes	Shark attack	Euthanized
50	Caretta caretta	2000	2000-07-26	2000-07-28			Unknown/undetermined		Released
51	Caretta caretta	2000	2000-07-26	2000-07-28			Unknown/undetermined		Released
52	Caretta caretta	2000	2000-07-27	2000-07-28			Hooks/monofilament lines		Released
53	Caretta caretta	2000	2000-07-28	2000-07-29			Hooks/monofilament lines		Died during hospitalization
54	Caretta caretta	2000	2000-07-28	2000-07-28			Unknown/undetermined		Died during hospitalization
55	Caretta caretta	2000	2000-07-29	2000-08-15			Entanglement		Released
56	Caretta caretta	2000	2000-07-31	2000-08-02			Unknown/undetermined		Released
57	Caretta caretta	2000	2000-08-04	2000-08-15			other causes	Miscellany	Released
58	Caretta caretta	2000	2000-08-05	2000-12-31			trauma		Euthanized
59	Caretta caretta	2000	2000-08-17	2000-12-20			Unknown/undetermined		Released
60	Caretta caretta	2000	2000-08-19	2000-08-23			Entanglement		Released
61	Caretta caretta	2000	2000-08-21	2000-08-30			Unknown/undetermined		Released
62	Caretta caretta	2000	2000-08-21	2000-08-30			Unknown/undetermined		Released
63	Caretta caretta	2000	2000-08-24	2000-08-29			Hooks/monofilament lines		Released
64	Caretta caretta	2000	2000-08-28	2000-09-04			Hooks/monofilament lines		Released
65	Caretta caretta	2000	2000-08-30	2000-09-04			Entanglement		Released
66	Caretta caretta	2000	2000-09-02	2000-09-09			Entanglement		Released

Record ID	Species	Year	Admission date	Departure date	SCL (cm)	Body weight (kg)	Cause of admission	Other-cause subcategory	Final disposition
67	Caretta caretta	2000	2000-09-04	2000-09-07			Unknown/undetermined		Released
68	Caretta caretta	2000	2000-09-06	2000-09-20			Unknown/undetermined		Released
69	Caretta caretta	2000	2000-09-06	2000-09-07			Crude oil		Released
70	Caretta caretta	2000	2000-09-06	2000-09-07			Crude oil		Released
71	Caretta caretta	2000	2000-09-06	2000-09-09			Crude oil		Released
72	Caretta caretta	2000	2000-09-11				Unknown/undetermined		Admitted dead
73	Caretta caretta	2000	2000-09-11	2000-10-05			Entanglement		Died during hospitalization
74	Caretta caretta	2000	2000-09-12	2000-09-13			Unknown/undetermined		Released
75	Caretta caretta	2000	2000-09-12	2000-09-13			Unknown/undetermined		Released
76	Caretta caretta	2000	2000-09-12	2000-11-14			Unknown/undetermined		Released
77	Caretta caretta	2000	2000-09-12	2000-11-07			Unknown/undetermined		Died during hospitalization
78	Caretta caretta	2000	2000-09-17	2000-09-19			Unknown/undetermined		Died during hospitalization
79	Caretta caretta	2000	2000-09-19	2000-09-22			Hooks/monofilament lines		Released
80	Caretta caretta	2000	2000-09-23				Unknown/undetermined		Admitted dead
81	Caretta caretta	2000	2000-09-26	2000-09-30			Unknown/undetermined		Released
82	Caretta caretta	2000	2000-09-30				Unknown/undetermined		Admitted dead
83	Caretta caretta	2000	2000-10-04	2000-10-16			Unknown/undetermined		Released
84	Caretta caretta	2000	2000-10-06	2000-10-16			Unknown/undetermined		Released
85	Caretta caretta	2000	2000-10-06	2000-11-02			Entanglement		Released
86	Caretta caretta	2000	2000-10-20	2000-10-25			Unknown/undetermined		Released
87	Caretta caretta	2000	2000-10-22	2000-10-25			Entanglement		Released
88	Caretta caretta	2000	2000-10-23	2000-10-24			Entanglement		Released
89	Caretta caretta	2000	2000-12-05	2001-01-06			Hooks/monofilament lines		Died during hospitalization
90	Caretta caretta	2001	2001-03-17	2001-04-18	56	9	Infectious disease		Released
91	Caretta caretta	2001	2001-04-01	2001-04-02			Infectious disease		Died during hospitalization
92	Caretta caretta	2001	2001-04-01	2001-04-03			Infectious disease		Died during hospitalization
93	Caretta caretta	2001	2001-04-04		53	15	Hooks/monofilament lines		Admitted dead
94	Caretta caretta	2001	2001-04-10	2001-04-23	40		Hooks/monofilament lines		Released
95	Caretta caretta	2001	2001-04-14	2001-04-23			Entanglement		Released
96	Caretta caretta	2001	2001-04-15	2001-06-08			Infectious disease		Released
97	Caretta caretta	2001	2001-04-27	2001-06-26	46.5		Infectious disease		Released
98	Caretta caretta	2001	2001-04-27	2001-04-28			Entanglement		Died during hospitalization
99	Caretta caretta	2001	2001-05-01	2002-01-23			Hooks/monofilament lines		Released
100	Caretta caretta	2001	2001-05-03	2001-11-09			Hooks/monofilament lines		Released
101	Caretta caretta	2001	2001-05-04				Unknown/undetermined		Admitted dead
102	Caretta caretta	2001	2001-05-11	2001-05-15			Infectious disease		Died during hospitalization
103	Caretta caretta	2001	2001-05-14				Unknown/undetermined		Admitted dead
104	Caretta caretta	2001	2001-05-14		35		Unknown/undetermined		Admitted dead
105	Caretta caretta	2001	2001-05-16	2001-05-17			trauma		Died during hospitalization
106	Caretta caretta	2001	2001-05-16				Unknown/undetermined		Admitted dead
107	Caretta caretta	2001	2001-05-16	2001-06-04	16.5	0.7	Infectious disease		Released
108	Caretta caretta	2001	2001-05-16	2001-06-04	16.2	0.7	Crude oil		Released
109	Caretta caretta	2001	2001-05-17	2001-06-27			other causes	Miscellany	Released
110	Caretta caretta	2001	2001-05-20	2001-05-20			Unknown/undetermined		Admitted dead
111	Caretta caretta	2001	2001-05-21	2001-05-21			Hooks/monofilament lines		Died during hospitalization
112	Caretta caretta	2001	2001-05-23	2001-05-28			Infectious disease		Died during hospitalization
113	Caretta caretta	2001	2001-05-30	2001-06-29			other causes	Miscellany	Released
114	Caretta caretta	2001	2001-05-30	2001-06-04			Crude oil		Released
115	Caretta caretta	2001	2001-06-02	2001-07-20			Infectious disease		Released
116	Caretta caretta	2001	2001-06-04	2001-06-29			other causes	Plastic ingestion	Died during hospitalization
117	Caretta caretta	2001	2001-06-05	2001-06-06			Entanglement		Died during hospitalization
118	Caretta caretta	2001	2001-06-08	2001-09-15	18	1	trauma		Released
119	Caretta caretta	2001	2001-06-08	2001-06-12	24.5	2.2	Unknown/undetermined		Released
120	Caretta caretta	2001	2001-06-08	2001-06-29	15.5	0.5	Infectious disease		Released
121	Caretta caretta	2001	2001-06-12	2001-07-23			Hooks/monofilament lines		Released
122	Caretta caretta	2001	2001-06-14	2001-08-14			Hooks/monofilament lines		Released
123	Caretta caretta	2001	2001-06-14	2001-07-06			Entanglement		Released
124	Caretta caretta	2001	2001-06-15	2001-06-16			trauma		Died during hospitalization
125	Caretta caretta	2001	2001-06-18	2001-06-29			Unknown/undetermined		Released
126	Caretta caretta	2001	2001-06-18	2001-08-03			Infectious disease		Released
127	Caretta caretta	2001	2001-06-19	2001-07-06			Unknown/undetermined		Released
128	Caretta caretta	2001	2001-06-20	2001-07-29			other causes	Malnutrition	Released
129	Caretta caretta	2001	2001-06-21	2001-06-26			Entanglement		Died during hospitalization
130	Caretta caretta	2001	2001-06-22				Unknown/undetermined		Admitted dead
131	Caretta caretta	2001	2001-06-22	2001-10-15			Infectious disease		Released
132	Caretta caretta	2001	2001-06-24	2001-06-29			other causes	Malnutrition	Released
133	Caretta caretta	2001	2001-06-24	2001-07-04			Entanglement		Released
134	Caretta caretta	2001	2001-06-25	2001-06-26	41.5		Hooks/monofilament lines		Died during hospitalization
135	Caretta caretta	2001	2001-06-25	2001-08-03			Infectious disease		Released
136	Caretta caretta	2001	2001-06-25	2001-08-08			Entanglement		Released
137	Caretta caretta	2001	2001-06-26	2001-07-15	19	1	Infectious disease		Released
138	Caretta caretta	2001	2001-06-26	2001-08-03	39		Entanglement		Released

Record ID	Species	Year	Admission date	Departure date	SCL (cm)	Body weight (kg)	Cause of admission	Other-cause subcategory	Final disposition
139	Caretta caretta	2001	2001-06-28	2001-07-03			Hooks/monofilament lines		Released
140	Caretta caretta	2001	2001-06-28	2001-07-20			Hooks/monofilament lines		Released
141	Caretta caretta	2001	2001-07-01	2001-08-08			Entanglement		Released
142	Caretta caretta	2001	2001-07-06	2001-07-09			Unknown/undetermined		Released
143	Caretta caretta	2001	2001-07-09	2001-07-23			other causes	Buoyancy disorder	Released
144	Caretta caretta	2001	2001-07-09	2001-07-19			Infectious disease		Died during hospitalization
145	Caretta caretta	2001	2001-07-10	2001-07-20			Infectious disease		Released
146	Caretta caretta	2001	2001-07-11	2001-07-20			Infectious disease		Released
147	Caretta caretta	2001	2001-07-14	2001-07-23	47		Infectious disease		Released
148	Caretta caretta	2001	2001-07-14	2001-07-23	52.5		Infectious disease		Released
149	Caretta caretta	2001	2001-07-25	2001-10-01	48	18.6	Hooks/monofilament lines		Released
150	Caretta caretta	2001	2001-07-25	2001-11-09	46	19.6	trauma		Released
151	Caretta caretta	2001	2001-07-27	2001-08-08			other causes	Buoyancy disorder	Released
152	Caretta caretta	2001	2001-07-30	2001-08-10			Crude oil		Released
153	Caretta caretta	2001	2001-07-30	2001-08-10			Crude oil		Released
154	Caretta caretta	2001	2001-07-30	2001-08-10			Crude oil		Released
155	Caretta caretta	2001	2001-07-30	2001-08-10			Crude oil		Released
156	Caretta caretta	2001	2001-07-31	2001-08-03			Crude oil		Released
157	Caretta caretta	2001	2001-07-31	2001-08-10			Crude oil		Released
158	Caretta caretta	2001	2001-07-31				Crude oil		Admitted dead
159	Caretta caretta	2001	2001-08-01				Crude oil		Admitted dead
160	Caretta caretta	2001	2001-08-01				Crude oil		Admitted dead
161	Caretta caretta	2001	2001-08-01				Crude oil		Admitted dead
162	Caretta caretta	2001	2001-08-01				Crude oil		Admitted dead
163	Caretta caretta	2001	2001-08-01				Crude oil		Admitted dead
164	Caretta caretta	2001	2001-08-01				Crude oil		Admitted dead
165	Caretta caretta	2001	2001-08-01				Crude oil		Admitted dead
166	Caretta caretta	2001	2001-08-02	2001-08-10			Crude oil		Released
167	Caretta caretta	2001	2001-08-05	2001-08-10			Crude oil		Released
168	Caretta caretta	2001	2001-08-06	2001-08-19			Infectious disease		Released
169	Caretta caretta	2001	2001-08-12	2001-08-29			Entanglement		Released
170	Caretta caretta	2001	2001-08-14	2001-08-17			Entanglement		Released
171	Caretta caretta	2001	2001-08-18	2001-08-23			Crude oil		Released
172	Caretta caretta	2001	2001-08-18	2001-08-28			Entanglement		Released
173	Caretta caretta	2001	2001-08-20	2001-08-21	19	1.2	Unknown/undetermined		Released
174	Caretta caretta	2001	2001-08-28	2001-09-19	38		Entanglement		Released
175	Caretta caretta	2001	2001-09-05	2001-09-11			trauma		Released
176	Caretta caretta	2001	2001-09-07	2001-09-16			trauma		Died during hospitalization
177	Caretta caretta	2001	2001-09-08	2001-09-20			Infectious disease		Released
178	Caretta caretta	2001	2001-09-10	2001-09-20	45	13	trauma		Released
179	Caretta caretta	2001	2001-09-10	2001-09-11			Unknown/undetermined		Released
180	Caretta caretta	2001	2001-09-10	2001-09-20		7.7	Entanglement		Released
181	Caretta caretta	2001	2001-10-04	2001-10-16		11	Entanglement		Released
182	Caretta caretta	2001	2001-10-10	2001-10-11			Infectious disease		Died during hospitalization
183	Caretta caretta	2001	2001-10-12	2001-11-02			Entanglement		Released
184	Caretta caretta	2001	2001-10-19	2001-10-19	40		Entanglement		Released
185	Caretta caretta	2001	2001-10-27	2002-06-05			Entanglement		Released
186	Caretta caretta	2001	2001-11-01	2001-11-13			Entanglement		Released
187	Caretta caretta	2001	2001-11-06	2001-11-09			Entanglement		Released
188	Caretta caretta	2001	2001-11-12		43.5		Entanglement		Admitted dead
189	Caretta caretta	2001	2001-11-20	2001-12-04			Hooks/monofilament lines		Released
190	Caretta caretta	2001	2001-11-22				Unknown/undetermined		Admitted dead
191	Caretta caretta	2001	2001-11-23	2001-12-04			Entanglement		Released
192	Caretta caretta	2001	2001-12-06	2001-12-20	25		Hooks/monofilament lines		Released
193	Caretta caretta	2001	2001-12-09	2001-12-09			Unknown/undetermined		Released
194	Caretta caretta	2001	2001-12-18	2001-12-20	55		Entanglement		Released
195	Caretta caretta	2001	2001-12-22	2002-01-06			Unknown/undetermined		Died during hospitalization
196	Caretta caretta	2001	2001-12-26	2002-01-11	33		Entanglement		Died during hospitalization
197	Caretta caretta	2002	2002-01-08	2002-03-19			Hooks/monofilament lines		Released
198	Caretta caretta	2002	2002-03-25	2002-04-20			Entanglement		Released
199	Caretta caretta	2002	2002-03-26	2002-04-20			Hooks/monofilament lines		Released
200	Caretta caretta	2002	2002-04-02	2002-04-12	35		Entanglement		Released
201	Caretta caretta	2002	2002-04-10	2002-05-24			Entanglement		Released
202	Caretta caretta	2002	2002-04-14				Unknown/undetermined		Admitted dead
203	Caretta caretta	2002	2002-04-21				Unknown/undetermined		Admitted dead
204	Caretta caretta	2002	2002-04-24	2002-06-26			Hooks/monofilament lines		Euthanized
205	Caretta caretta	2002	2002-04-24	2002-05-13			Entanglement		Released
206	Caretta caretta	2002	2002-05-13	2002-05-13			Unknown/undetermined		Released
207	Caretta caretta	2002	2002-05-14	2002-05-16			trauma		Died during hospitalization
208	Caretta caretta	2002	2002-05-15	2002-08-30			Entanglement		Released
209	Caretta caretta	2002	2002-05-16	2002-07-09	48	20	Hooks/monofilament lines		Died during hospitalization
210	Caretta caretta	2002	2002-05-18	2002-06-13			Hooks/monofilament lines		Released

Record ID	Species	Year	Admission date	Departure date	SCL (cm)	Body weight (kg)	Cause of admission	Other-cause subcategory	Final disposition
211	Caretta caretta	2002	2002-05-18	2002-07-31			trauma		Released
212	Caretta caretta	2002	2002-05-18				Unknown/undetermined		Admitted dead
213	Caretta caretta	2002	2002-05-18	2002-05-21	47		Crude oil		Released
214	Caretta caretta	2002	2002-05-26	2003-05-30			Infectious disease		Released
215	Caretta caretta	2002	2002-05-26	2002-07-10			Entanglement		Released
216	Caretta caretta	2002	2002-05-27	2002-06-27			Hooks/monofilament lines		Died during hospitalization
217	Caretta caretta	2002	2002-05-28	2002-05-30			Hooks/monofilament lines		Died during hospitalization
218	Caretta caretta	2002	2002-06-01	2002-06-19			Entanglement		Released
219	Caretta caretta	2002	2002-06-03				Entanglement		Admitted dead
220	Caretta caretta	2002	2002-06-04	2002-06-05			Hooks/monofilament lines		Died during hospitalization
221	Caretta caretta	2002	2002-06-07	2002-06-24			Hooks/monofilament lines		Released
222	Caretta caretta	2002	2002-06-10		50		Entanglement		Admitted dead
223	Caretta caretta	2002	2002-06-15	2002-06-19			Unknown/undetermined		Released
224	Caretta caretta	2002	2002-06-21	2002-08-13			Hooks/monofilament lines		Released
225	Caretta caretta	2002	2002-06-23	2002-06-25			Entanglement		Euthanized
226	Caretta caretta	2002	2002-06-24				Unknown/undetermined		Admitted dead
227	Caretta caretta	2002	2002-06-27	2002-08-13			Entanglement		Released
228	Caretta caretta	2002	2002-06-29	2002-06-29			other causes	Malnutrition	Released
229	Caretta caretta	2002	2002-07-01	2002-07-19			Hooks/monofilament lines		Released
230	Caretta caretta	2002	2002-07-02				Unknown/undetermined		Admitted dead
231	Caretta caretta	2002	2002-07-05	2002-07-16			Entanglement		Released
232	Caretta caretta	2002	2002-07-08	2002-07-19			Entanglement		Released
233	Caretta caretta	2002	2002-07-09	2002-07-19			Hooks/monofilament lines		Released
234	Caretta caretta	2002	2002-07-10				Unknown/undetermined		Admitted dead
235	Caretta caretta	2002	2002-07-10				Unknown/undetermined		Admitted dead
236	Caretta caretta	2002	2002-07-13	2002-07-19			Entanglement		Released
237	Caretta caretta	2002	2002-07-23	2002-08-12			Hooks/monofilament lines		Released
238	Caretta caretta	2002	2002-07-25	2002-08-02	31	6.4	trauma		Released
239	Caretta caretta	2002	2002-07-29				Hooks/monofilament lines		Admitted dead
240	Caretta caretta	2002	2002-08-02	2002-08-12			Entanglement		Released
241	Caretta caretta	2002	2002-08-06	2002-08-06			other causes	Shark attack	Died during hospitalization
242	Caretta caretta	2002	2002-08-06	2002-08-12			Entanglement		Released
243	Caretta caretta	2002	2002-08-07	2002-08-12			Entanglement		Released
244	Caretta caretta	2002	2002-08-11	2002-08-18			Hooks/monofilament lines		Released
245	Caretta caretta	2002	2002-08-12	2002-08-22			Hooks/monofilament lines		Died during hospitalization
246	Caretta caretta	2002	2002-08-12				Entanglement		Admitted dead
247	Caretta caretta	2002	2002-08-13	2002-08-21			Entanglement		Released
248	Caretta caretta	2002	2002-08-14				Crude oil		Admitted dead
249	Caretta caretta	2002	2002-08-15	2002-08-21			Entanglement		Released
250	Caretta caretta	2002	2002-08-16	2002-08-30			Entanglement		Released
251	Caretta caretta	2002	2002-08-18	2002-09-20			Infectious disease		Died during hospitalization
252	Caretta caretta	2002	2002-08-19				trauma		Admitted dead
253	Caretta caretta	2002	2002-08-20	2002-09-15			Hooks/monofilament lines		Died during hospitalization
254	Caretta caretta	2002	2002-08-20	2002-08-30			Entanglement		Released
255	Caretta caretta	2002	2002-08-22	2002-08-23		22	Hooks/monofilament lines		Died during hospitalization
256	Caretta caretta	2002	2002-08-24	2002-09-05			Hooks/monofilament lines		Died during hospitalization
257	Caretta caretta	2002	2002-08-24	2002-08-29			Infectious disease		Died during hospitalization
258	Caretta caretta	2002	2002-08-24	2002-09-03			Entanglement		Released
259	Caretta caretta	2002	2002-09-02	2002-09-06			Hooks/monofilament lines		Released
260	Caretta caretta	2002	2002-09-03	2002-11-07			Hooks/monofilament lines		Released
261	Caretta caretta	2002	2002-09-05	2002-10-01			Entanglement		Released
262	Caretta caretta	2002	2002-09-08				trauma		Admitted dead
263	Caretta caretta	2002	2002-09-09	2002-10-16			Entanglement		Released
264	Caretta caretta	2002	2002-09-09	2002-09-09			Entanglement		Died during hospitalization
265	Caretta caretta	2002	2002-09-10	2002-09-12			Hooks/monofilament lines		Released
266	Caretta caretta	2002	2002-09-12	2002-09-13			other causes	Malnutrition	Released
267	Caretta caretta	2002	2002-09-19	2002-09-25			Entanglement		Released
268	Caretta caretta	2002	2002-09-22	2002-10-01			Entanglement		Released
269	Caretta caretta	2002	2002-09-24	2002-11-07			Entanglement		Released
270	Caretta caretta	2002	2002-09-30	2002-11-07			Entanglement		Released
271	Caretta caretta	2002	2002-10-04	2002-10-16			Entanglement		Released
272	Caretta caretta	2002	2002-10-09	2002-10-22			Entanglement		Released
273	Caretta caretta	2002	2002-10-10	2002-10-25			Entanglement		Released
274	Caretta caretta	2002	2002-10-11	2002-10-14			Entanglement		Released
275	Caretta caretta	2002	2002-10-13	2002-11-18			Entanglement		Died during hospitalization
276	Caretta caretta	2002	2002-10-14	2002-10-29			Hooks/monofilament lines		Released
277	Caretta caretta	2002	2002-10-18	2002-11-05			Entanglement		Died during hospitalization
278	Caretta caretta	2002	2002-10-30				Unknown/undetermined		Admitted dead
279	Caretta caretta	2002	2002-11-02	2002-11-08			Entanglement		Released
280	Caretta caretta	2002	2002-11-02	2002-11-08			Entanglement		Released
281	Caretta caretta	2002	2002-11-17	2002-11-20			Unknown/undetermined		Released
282	Caretta caretta	2002	2002-11-19	2002-12-08			Entanglement		Died during hospitalization

Record ID	Species	Year	Admission date	Departure date	SCL (cm)	Body weight (kg)	Cause of admission	Other-cause subcategory	Final disposition
283	Caretta caretta	2002	2002-11-20				Unknown/undetermined		Admitted dead
284	Caretta caretta	2002	2002-11-25	2002-11-29			Entangement		Released
285	Caretta caretta	2002	2002-12-03	2002-12-11			Crude oil		Released
286	Caretta caretta	2002	2002-12-07	2002-12-27			Entanglement		Released
287	Caretta caretta	2002	2002-12-30	2003-01-19			Entanglement		Died during hospitalization
288	Caretta caretta	2003	2003-03-10	2003-03-17	33		Infectious disease		Released
289	Caretta caretta	2003	2003-03-16	2003-03-18			Infectious disease		Died during hospitalization
290	Caretta caretta	2003	2003-03-16	2003-03-17			Entanglement		Released
291	Caretta caretta	2003	2003-03-23	2003-05-30	49.5		Entanglement		Released
292	Caretta caretta	2003	2003-04-01	2003-04-03			Entanglement		Euthanized
293	Caretta caretta	2003	2003-04-03	2003-04-03			trauma		Died during hospitalization
294	Caretta caretta	2003	2003-04-08	2003-05-26			Entanglement		Released
295	Caretta caretta	2003	2003-04-10	2003-04-11			Entanglement		Released
296	Caretta caretta	2003	2003-04-10	2003-05-01			Entanglement		Released
297	Caretta caretta	2003	2003-04-10	2003-05-01			Entanglement		Released
298	Caretta caretta	2003	2003-04-11	2003-04-16			Crude oil		Released
299	Caretta caretta	2003	2003-04-15	2003-05-22			Entanglement		Released
300	Caretta caretta	2003	2003-04-17	2003-04-22			Entanglement		Died during hospitalization
301	Caretta caretta	2003	2003-04-19	2003-04-28			Infectious disease		Released
302	Caretta caretta	2003	2003-04-19	2003-05-01			Entanglement		Released
303	Caretta caretta	2003	2003-04-19	2003-05-01			Entanglement		Released
304	Caretta caretta	2003	2003-04-20	2004-05-15	38	9.5	Entanglement		Released
305	Caretta caretta	2003	2003-04-27	2003-04-30			Hooks/monofilament lines		Died during hospitalization
306	Caretta caretta	2003	2003-04-27	2003-05-14			Entanglement		Released
307	Caretta caretta	2003	2003-04-29	2003-04-30			Hooks/monofilament lines		Euthanized
308	Caretta caretta	2003	2003-04-30	2003-05-14			Hooks/monofilament lines		Released
309	Caretta caretta	2003	2003-04-30	2003-07-11			Entanglement		Released
310	Caretta caretta	2003	2003-05-02	2003-05-26			Entanglement		Released
311	Caretta caretta	2003	2003-05-06	2003-11-27			Infectious disease		Released
312	Caretta caretta	2003	2003-05-08	2003-05-19			Hooks/monofilament lines		Released
313	Caretta caretta	2003	2003-05-10	2003-06-16			other causes	Buoyancy disorder	Died during hospitalization
314	Caretta caretta	2003	2003-05-10	2003-06-11			Infectious disease		Died during hospitalization
315	Caretta caretta	2003	2003-05-11	2003-05-26			Infectious disease		Released
316	Caretta caretta	2003	2003-05-15				Unknown/undetermined		Admitted dead
317	Caretta caretta	2003	2003-05-15				Unknown/undetermined		Admitted dead
318	Caretta caretta	2003	2003-05-16	2003-08-13			Entanglement		Died during hospitalization
319	Caretta caretta	2003	2003-05-17	2003-06-27			trauma		Released
320	Caretta caretta	2003	2003-05-17	2003-11-24			trauma		Released
321	Caretta caretta	2003	2003-05-18	2003-07-11			Hooks/monofilament lines		Released
322	Caretta caretta	2003	2003-05-18	2003-05-26			Infectious disease		Released
323	Caretta caretta	2003	2003-05-26	2003-06-12			Hooks/monofilament lines		Died during hospitalization
324	Caretta caretta	2003	2003-05-26	2003-05-26			Unknown/undetermined		Released
325	Caretta caretta	2003	2003-05-28	2003-09-04			Infectious disease		Released
326	Caretta caretta	2003	2003-05-28	2003-06-03			Entanglement		Released
327	Caretta caretta	2003	2003-05-30	2003-06-10			Entanglement		Released
328	Caretta caretta	2003	2003-06-01	2003-06-10			Entanglement		Released
329	Caretta caretta	2003	2003-06-02	2003-06-10			Infectious disease		Released
330	Caretta caretta	2003	2003-06-05	2003-06-10			Entanglement		Died during hospitalization
331	Caretta caretta	2003	2003-06-07	2003-06-27			Infectious disease		Released
332	Caretta caretta	2003	2003-06-07	2003-07-01			Infectious disease		Released
333	Caretta caretta	2003	2003-06-08	2003-06-30			Infectious disease		Released
334	Caretta caretta	2003	2003-06-08				Entanglement		Admitted dead
335	Caretta caretta	2003	2003-06-09	2003-06-13			other causes	Malnutrition	Released
336	Caretta caretta	2003	2003-06-11				Unknown/undetermined		Admitted dead
337	Caretta caretta	2003	2003-06-15	2003-06-16			Unknown/undetermined		Released
338	Caretta caretta	2003	2003-06-15	2003-06-16			Unknown/undetermined		Released
339	Caretta caretta	2003	2003-06-15	2003-06-16			Unknown/undetermined		Released
340	Caretta caretta	2003	2003-06-15	2003-06-27			Crude oil		Released
341	Caretta caretta	2003	2003-06-15	2003-06-16			Entanglement		Released
342	Caretta caretta	2003	2003-06-18	2003-06-27			Crude oil		Released
343	Caretta caretta	2003	2003-06-18	2003-07-11			Entanglement		Released
344	Caretta caretta	2003	2003-06-22	2003-07-04			Entanglement		Released
345	Caretta caretta	2003	2003-06-22	2003-11-24			Entanglement		Released
346	Caretta caretta	2003	2003-06-23	2003-06-23			Entanglement		Released
347	Caretta caretta	2003	2003-06-25				Unknown/undetermined		Admitted dead
348	Caretta caretta	2003	2003-06-25				Crude oil		Admitted dead
349	Caretta caretta	2003	2003-06-25	2003-07-01			Entanglement		Released
350	Caretta caretta	2003	2003-06-26				Unknown/undetermined		Admitted dead
351	Caretta caretta	2003	2003-06-28	2003-07-08			other causes	Miscellany	Released
352	Caretta caretta	2003	2003-06-28	2003-07-24			Entanglement		Released
353	Caretta caretta	2003	2003-06-29	2003-10-15			trauma		Released
354	Caretta caretta	2003	2003-06-29	2003-07-08			other causes	Miscellany	Released

Record ID	Species	Year	Admission date	Departure date	SCL (cm)	Body weight (kg)	Cause of admission	Other-cause subcategory	Final disposition
355	Caretta caretta	2003	2003-06-29	2003-08-21			Entanglement		Released
356	Caretta caretta	2003	2003-07-01		37		Entanglement		Admitted dead
357	Caretta caretta	2003	2003-07-02	2003-10-15			Hooks/monofilament lines		Released
358	Caretta caretta	2003	2003-07-03	2003-08-15			Entanglement		Released
359	Caretta caretta	2003	2003-07-04	2003-07-07			other causes	Malnutrition	Released
360	Caretta caretta	2003	2003-07-04	2003-07-07			Unknown/undetermined		Died during hospitalization
361	Caretta caretta	2003	2003-07-09	2003-08-08			Infectious disease		Released
362	Caretta caretta	2003	2003-07-12	2003-08-20			Infectious disease		Released
363	Caretta caretta	2003	2003-07-16	2003-07-16			Unknown/undetermined		Released
364	Caretta caretta	2003	2003-07-18	2003-07-23			Infectious disease		Released
365	Caretta caretta	2003	2003-07-22	2003-07-27			Entanglement		Released
366	Caretta caretta	2003	2003-07-22	2003-08-04			Entanglement		Released
367	Caretta caretta	2003	2003-07-23	2003-08-04			Entanglement		Released
368	Caretta caretta	2003	2003-07-24	2003-07-24			Unknown/undetermined		Released
369	Caretta caretta	2003	2003-07-28	2003-08-20			Crude oil		Released
370	Caretta caretta	2003	2003-07-31	2003-08-05			Infectious disease		Died during hospitalization
371	Caretta caretta	2003	2003-08-01	2003-08-20			Hooks/monofilament lines		Released
372	Caretta caretta	2003	2003-08-01	2003-08-06			trauma		Released
373	Caretta caretta	2003	2003-08-03	2003-08-06			other causes	Miscellany	Released
374	Caretta caretta	2003	2003-08-07	2003-08-11			trauma		Released
375	Caretta caretta	2003	2003-08-07	2003-08-07			Unknown/undetermined		Released
376	Caretta caretta	2003	2003-08-07	2003-09-10			Infectious disease		Died during hospitalization
377	Caretta caretta	2003	2003-08-07	2003-08-08	31	6.4	Infectious disease		Died during hospitalization
378	Caretta caretta	2003	2003-08-08	2003-08-11			Entanglement		Released
379	Caretta caretta	2003	2003-08-11	2003-08-16			Entanglement		Released
380	Caretta caretta	2003	2003-08-11	2003-08-27			Entanglement		Released
381	Caretta caretta	2003	2003-08-12	2003-08-13			Entanglement		Euthanized
382	Caretta caretta	2003	2003-08-15	2003-08-15			Entanglement		Released
383	Caretta caretta	2003	2003-08-15	2003-08-24			Entanglement		Released
384	Caretta caretta	2003	2003-08-22	2003-08-25			Entanglement		Released
385	Caretta caretta	2003	2003-09-03	2003-09-22			Entanglement		Released
386	Caretta caretta	2003	2003-09-08	2003-10-15			trauma		Released
387	Caretta caretta	2003	2003-09-14	2003-09-17			other causes	Malnutrition	Released
388	Caretta caretta	2003	2003-09-14	2003-10-15			Infectious disease		Released
389	Caretta caretta	2003	2003-09-29	2003-10-07			Infectious disease		Died during hospitalization
390	Caretta caretta	2003	2003-10-19	2003-12-13			Entanglement		Released
391	Caretta caretta	2003	2003-10-24	2003-10-24			Entanglement		Released
392	Caretta caretta	2003	2003-11-18	2003-12-13			Entanglement		Released
393	Caretta caretta	2004	2004-01-19				trauma		Admitted dead
394	Caretta caretta	2004	2004-02-22	2004-05-26	46	14.2	Entanglement		Released
395	Caretta caretta	2004	2004-03-11	2004-04-14	51	21.7	Entanglement		Released
396	Caretta caretta	2004	2004-04-04	2004-04-16	55	25.1	Infectious disease		Released
397	Caretta caretta	2004	2004-04-06	2004-04-16	38	8.7	Entanglement		Released
398	Caretta caretta	2004	2004-04-07	2004-04-15	52	22.2	Entanglement		Released
399	Caretta caretta	2004	2004-04-07	2004-04-11			Entanglement		Died during hospitalization
400	Caretta caretta	2004	2004-04-11	2004-06-09			Entanglement		Released
401	Caretta caretta	2004	2004-04-13	2004-06-21	17.2	0.9	Entanglement		Released
402	Caretta caretta	2004	2004-04-27	2004-04-29			Entanglement		Died during hospitalization
403	Caretta caretta	2004	2004-04-29	2004-07-06	39	8	Entanglement		Released
404	Caretta caretta	2004	2004-05-06	2004-09-04	29.8	4	Entanglement		Released
405	Caretta caretta	2004	2004-05-09	2004-05-11			Entanglement		Released
406	Caretta caretta	2004	2004-05-12	2004-08-26	18	0.9	Infectious disease		Released
407	Caretta caretta	2004	2004-05-27	2004-07-06	37	8	other causes	Shark attack	Released
408	Caretta caretta	2004	2004-05-27				Entanglement		Admitted dead
409	Caretta caretta	2004	2004-05-29	2004-05-29			Entanglement		Released
410	Caretta caretta	2004	2004-05-29	2004-06-21	45	14.6	Entanglement		Released
411	Caretta caretta	2004	2004-06-09	2004-06-21	21.3	1.5	other causes	Buoyancy disorder	Released
412	Caretta caretta	2004	2004-06-12	2004-06-12			Unknown/undetermined		Released
413	Caretta caretta	2004	2004-06-27	2004-07-26	20	1.4	Entanglement		Released
414	Caretta caretta	2004	2004-06-27	2004-07-26	36	8	Entanglement		Released
415	Caretta caretta	2004	2004-07-01	2004-12-04			other causes	Malnutrition	Released
416	Caretta caretta	2004	2004-07-02	2004-07-05			Unknown/undetermined		Released
417	Caretta caretta	2004	2004-07-07	2004-07-16			Entanglement		Released
418	Caretta caretta	2004	2004-07-15				Unknown/undetermined		Admitted dead
419	Caretta caretta	2004	2004-07-17				Entanglement		Admitted dead
420	Caretta caretta	2004	2004-07-19	2004-07-23			Crude oil		Released
421	Caretta caretta	2004	2004-07-21	2004-08-26			Entanglement		Released
422	Caretta caretta	2004	2004-07-22				Unknown/undetermined		Admitted dead
423	Caretta caretta	2004	2004-07-22				Unknown/undetermined		Admitted dead
424	Caretta caretta	2004	2004-07-28	2004-08-11			Entanglement		Released
425	Caretta caretta	2004	2004-07-30	2004-07-30			Unknown/undetermined		Released
426	Caretta caretta	2004	2004-08-01	2004-08-01			Entanglement		Released

Record ID	Species	Year	Admission date	Departure date	SCL (cm)	Body weight (kg)	Cause of admission	Other-cause subcategory	Final disposition
427	Caretta caretta	2004	2004-08-02	2004-08-26			Entanglement		Released
428	Caretta caretta	2004	2004-08-04	2004-08-05			Entanglement		Released
429	Caretta caretta	2004	2004-08-10	2004-08-13			Unknown/undetermined		Released
430	Caretta caretta	2004	2004-08-11	2004-08-26			Hooks/monofilament lines		Released
431	Caretta caretta	2004	2004-08-11	2004-09-03			Infectious disease		Died during hospitalization
432	Caretta caretta	2004	2004-08-11				Entanglement		Admitted dead
433	Caretta caretta	2004	2004-08-13	2004-08-26			Entanglement		Released
434	Caretta caretta	2004	2004-08-13	2004-10-13			Entanglement		Released
435	Caretta caretta	2004	2004-08-17	2004-09-03			Entanglement		Released
436	Caretta caretta	2004	2004-08-18	2004-11-26			Infectious disease		Released
437	Caretta caretta	2004	2004-08-21	2004-11-26			other causes	Miscellany	Released
438	Caretta caretta	2004	2004-08-28	2004-11-26			Entanglement		Released
439	Caretta caretta	2004	2004-09-06	2004-09-09			Infectious disease		Released
440	Caretta caretta	2004	2004-09-14				Unknown/undetermined		Admitted dead
441	Caretta caretta	2004	2004-10-03	2004-12-04			Unknown/undetermined		Released
442	Caretta caretta	2004	2004-10-05	2004-12-04			Entanglement		Released
443	Caretta caretta	2004	2004-10-10				Unknown/undetermined		Admitted dead
444	Caretta caretta	2004	2004-10-13				Unknown/undetermined		Admitted dead
445	Caretta caretta	2004	2004-11-09	2004-12-15			Entanglement		Released
446	Caretta caretta	2005	2005-01-07	2005-03-06			Entanglement		Euthanized
447	Caretta caretta	2005	2005-01-25	2005-04-20			Entanglement		Released
448	Caretta caretta	2005	2005-01-26				other causes	Miscellany	Admitted dead
449	Caretta caretta	2005	2005-03-07				trauma		Admitted dead
450	Caretta caretta	2005	2005-03-12	2005-03-22			Entanglement		Released
451	Caretta caretta	2005	2005-03-24	2005-03-28			Entanglement		Died during hospitalization
452	Caretta caretta	2005	2005-03-26	2005-04-01			Infectious disease		Died during hospitalization
453	Caretta caretta	2005	2005-03-27	2005-04-21			Hooks/monofilament lines		Released
454	Caretta caretta	2005	2005-03-30	2005-04-01			Entanglement		Released
455	Caretta caretta	2005	2005-03-30	2005-04-05			Entanglement		Released
456	Caretta caretta	2005	2005-04-20	2005-06-25			Infectious disease		Released
457	Caretta caretta	2005	2005-04-26				Entanglement		Admitted dead
458	Caretta caretta	2005	2005-05-06				Crude oil		Admitted dead
459	Caretta caretta	2005	2005-05-15	2005-05-25			Entanglement		Released
460	Caretta caretta	2005	2005-05-18	2005-05-25			Entanglement		Released
461	Caretta caretta	2005	2005-05-31				Unknown/undetermined		Admitted dead
462	Caretta caretta	2005	2005-06-06	2005-06-10			Entanglement		Released
463	Caretta caretta	2005	2005-06-06	2005-06-10			Entanglement		Released
464	Caretta caretta	2005	2005-06-12	2005-06-24			Entanglement		Released
465	Caretta caretta	2005	2005-06-17	2005-06-24			Entanglement		Released
466	Caretta caretta	2005	2005-06-19	2005-06-24			Entanglement		Released
467	Caretta caretta	2005	2005-06-19	2005-07-04			Entanglement		Released
468	Caretta caretta	2005	2005-06-27	2005-07-04			Entanglement		Released
469	Caretta caretta	2005	2005-07-03	2005-12-21	31.5	4.7	Infectious disease		Released
470	Caretta caretta	2005	2005-07-12	2005-07-23			Crude oil		Released
471	Caretta caretta	2005	2005-07-13	2005-07-23			Crude oil		Released
472	Caretta caretta	2005	2005-07-15				Hooks/monofilament lines		Admitted dead
473	Caretta caretta	2005	2005-07-17	2005-10-21	48.5	16.7	Crude oil		Released
474	Caretta caretta	2005	2005-07-17	2005-07-23			Entanglement		Released
475	Caretta caretta	2005	2005-07-17	2005-07-27	28.7	3.7	Entanglement		Released
476	Caretta caretta	2005	2005-07-19				Unknown/undetermined		Admitted dead
477	Caretta caretta	2005	2005-07-19	2005-07-23			Entanglement		Released
478	Caretta caretta	2005	2005-07-19	2005-07-23			Entanglement		Released
479	Caretta caretta	2005	2005-07-20	2005-08-20			Entanglement		Released
480	Caretta caretta	2005	2005-07-20	2005-09-16			Entanglement		Released
481	Caretta caretta	2005	2005-07-23	2005-08-04			Entanglement		Released
482	Caretta caretta	2005	2005-07-23	2005-07-27	30	5.5	Entanglement		Released
483	Caretta caretta	2005	2005-07-24	2005-08-04			Entanglement		Released
484	Caretta caretta	2005	2005-07-25	2005-08-04			Entanglement		Released
485	Caretta caretta	2005	2005-07-26	2005-08-04			Entanglement		Released
486	Caretta caretta	2005	2005-07-27	2005-08-04			Entanglement		Released
487	Caretta caretta	2005	2005-07-31	2005-08-10			Entanglement		Released
488	Caretta caretta	2005	2005-08-01	2005-08-12			other causes	Malnutrition	Released
489	Caretta caretta	2005	2005-08-02	2005-08-04			Entanglement		Released
490	Caretta caretta	2005	2005-08-02	2005-08-10			Entanglement		Released
491	Caretta caretta	2005	2005-08-05	2005-08-12			Entanglement		Released
492	Caretta caretta	2005	2005-08-09	2005-08-12			Unknown/undetermined		Released
493	Caretta caretta	2005	2005-08-10	2005-08-20			Entanglement		Released
494	Caretta caretta	2005	2005-08-12	2005-08-20			Entanglement		Released
495	Caretta caretta	2005	2005-08-16	2005-08-20			Hooks/monofilament lines		Released
496	Caretta caretta	2005	2005-08-16	2005-08-20			Entanglement		Released
497	Caretta caretta	2005	2005-08-20	2005-09-02			Entanglement		Released
498	Caretta caretta	2005	2005-08-24	2005-09-02			Entanglement		Released

Record ID	Species	Year	Admission date	Departure date	SCL (cm)	Body weight (kg)	Cause of admission	Other-cause subcategory	Final disposition
499	Caretta caretta	2005	2005-08-26	2005-09-02			Entanglement		Released
500	Caretta caretta	2005	2005-08-27	2005-09-02			Entanglement		Released
501	Caretta caretta	2005	2005-08-28	2005-09-02			Crude oil		Released
502	Caretta caretta	2005	2005-08-30	2005-10-21			Infectious disease		Released
503	Caretta caretta	2005	2005-08-30	2005-09-09			Crude oil		Released
504	Caretta caretta	2005	2005-08-30	2005-09-09			Crude oil		Released
505	Caretta caretta	2005	2005-08-30				Crude oil		Admitted dead
506	Caretta caretta	2005	2005-08-30				Crude oil		Admitted dead
507	Caretta caretta	2005	2005-08-31	2005-09-09			Crude oil		Released
508	Caretta caretta	2005	2005-09-05	2005-09-16			Entanglement		Released
509	Caretta caretta	2005	2005-09-12	2005-09-16			Entanglement		Released
510	Caretta caretta	2005	2005-09-14	2005-10-02			Entanglement		Released
511	Caretta caretta	2005	2005-09-16	2005-10-15			Entanglement		Released
512	Caretta caretta	2005	2005-09-21				Entanglement		Admitted dead
513	Caretta caretta	2005	2005-09-22	2005-10-02			Entanglement		Released
514	Caretta caretta	2005	2005-10-02				Unknown/undetermined		Admitted dead
515	Caretta caretta	2005	2005-10-06	2005-10-16			Entanglement		Released
516	Caretta caretta	2005	2005-10-11	2005-10-21			Entanglement		Released
517	Caretta caretta	2005	2005-10-19	2005-11-01			Entanglement		Released
518	Caretta caretta	2005	2005-10-26	2005-11-05			Entanglement		Released
519	Caretta caretta	2005	2005-10-26	2005-12-21			Entanglement		Released
520	Caretta caretta	2005	2005-11-01	2005-11-05			other causes	Miscellany	Released
521	Caretta caretta	2005	2005-11-01	2005-11-05			other causes	Miscellany	Released
522	Caretta caretta	2005	2005-11-01	2005-11-05			other causes	Miscellany	Released
523	Caretta caretta	2005	2005-11-10	2005-11-24			Entanglement		Released
524	Caretta caretta	2005	2005-11-10	2005-11-18			Entanglement		Died during hospitalization
525	Caretta caretta	2005	2005-11-13	2005-12-27			Entanglement		Died during hospitalization
526	Caretta caretta	2006	2006-02-08				Unknown/undetermined		Admitted dead
527	Caretta caretta	2006	2006-03-04	2006-03-11			Infectious disease		Released
528	Caretta caretta	2006	2006-03-09	2006-04-10			Entanglement		Released
529	Caretta caretta	2006	2006-03-11	2006-03-23			Infectious disease		Released
530	Caretta caretta	2006	2006-03-11	2006-03-31			Entanglement		Released
531	Caretta caretta	2006	2006-03-25	2006-03-31			Entanglement		Released
532	Caretta caretta	2006	2006-04-01	2006-04-25			Entanglement		Released
533	Caretta caretta	2006	2006-04-08	2006-04-10			Entanglement		Euthanized
534	Caretta caretta	2006	2006-04-09	2006-04-25			Entanglement		Released
535	Caretta caretta	2006	2006-04-21	2006-04-30			Infectious disease		Released
536	Caretta caretta	2006	2006-04-21				Entanglement		Admitted dead
537	Caretta caretta	2006	2006-04-28	2006-05-19			Infectious disease		Died during hospitalization
538	Caretta caretta	2006	2006-05-08	2006-05-12			Entanglement		Died during hospitalization
539	Caretta caretta	2006	2006-05-10				trauma		Admitted dead
540	Caretta caretta	2006	2006-05-12	2006-05-26			Entanglement		Released
541	Caretta caretta	2006	2006-05-13	2006-05-26			Entanglement		Released
542	Caretta caretta	2006	2006-05-14	2006-05-26			Entanglement		Released
543	Caretta caretta	2006	2006-05-14	2006-05-31			Entanglement		Released
544	Caretta caretta	2006	2006-05-15	2006-05-26			Entanglement		Released
545	Caretta caretta	2006	2006-05-20	2006-05-26			Entanglement		Released
546	Caretta caretta	2006	2006-05-24	2006-06-01			Entanglement		Released
547	Caretta caretta	2006	2006-05-27	2006-06-01			trauma		Released
548	Caretta caretta	2006	2006-06-03	2006-06-11			trauma		Released
549	Caretta caretta	2006	2006-06-04	2006-06-20			Infectious disease		Released
550	Caretta caretta	2006	2006-06-05	2006-06-16			Entanglement		Released
551	Caretta caretta	2006	2006-06-06	2006-06-16			Infectious disease		Released
552	Caretta caretta	2006	2006-06-08				Unknown/undetermined		Admitted dead
553	Caretta caretta	2006	2006-06-12	2006-06-16			Entanglement		Released
554	Caretta caretta	2006	2006-06-17	2006-06-18			Hooks/monofilament lines		Died during hospitalization
555	Caretta caretta	2006	2006-06-19	2006-07-05			Entanglement		Released
556	Caretta caretta	2006	2006-06-20	2006-07-05			Entanglement		Released
557	Caretta caretta	2006	2006-06-20	2006-07-05			Entanglement		Released
558	Caretta caretta	2006	2006-06-23	2006-07-11			Entanglement		Released
559	Caretta caretta	2006	2006-06-26	2006-07-01			Entanglement		Released
560	Caretta caretta	2006	2006-06-30	2006-07-15			Entanglement		Released
561	Caretta caretta	2006	2006-07-01				Unknown/undetermined		Admitted dead
562	Caretta caretta	2006	2006-07-01	2006-07-20			Infectious disease		Died during hospitalization
563	Caretta caretta	2006	2006-07-02	2006-08-11			other causes	Miscellany	Released
564	Caretta caretta	2006	2006-07-02	2006-07-03			Infectious disease		Died during hospitalization
565	Caretta caretta	2006	2006-07-04				Entanglement		Admitted dead
566	Caretta caretta	2006	2006-07-05	2006-07-11			Unknown/undetermined		Released
567	Caretta caretta	2006	2006-07-05	2006-08-13			Entanglement		Released
568	Caretta caretta	2006	2006-07-16	2006-07-28			Entanglement		Released
569	Caretta caretta	2006	2006-07-20	2006-07-28			Entanglement		Released
570	Caretta caretta	2006	2006-07-20	2006-08-11			Entanglement		Released

Record ID	Species	Year	Admission date	Departure date	SCL (cm)	Body weight (kg)	Cause of admission	Other-cause subcategory	Final disposition
571	Caretta caretta	2006	2006-07-24	2006-11-10			Infectious disease		Died during hospitalization
572	Caretta caretta	2006	2006-07-25	2006-08-04			Entanglement		Released
573	Caretta caretta	2006	2006-07-27	2006-08-11			Entanglement		Released
574	Caretta caretta	2006	2006-07-28	2006-07-28			other causes	Miscellany	Released
575	Caretta caretta	2006	2006-07-30	2006-08-11			trauma		Released
576	Caretta caretta	2006	2006-07-30	2006-08-11			other causes	Malnutrition	Released
577	Caretta caretta	2006	2006-07-31	2006-08-04			Entanglement		Released
578	Caretta caretta	2006	2006-08-08	2009-10-06			Infectious disease		Released
579	Caretta caretta	2006	2006-08-10	2006-08-11			other causes	Malnutrition	Released
580	Caretta caretta	2006	2006-08-10	2006-09-06			other causes	Malnutrition	Released
581	Caretta caretta	2006	2006-08-12	2006-08-12			Entanglement		Released
582	Caretta caretta	2006	2006-08-12	2006-08-28			Entanglement		Released
583	Caretta caretta	2006	2006-08-18	2006-08-30			Entanglement		Released
584	Caretta caretta	2006	2006-08-25	2006-08-30			Entanglement		Released
585	Caretta caretta	2006	2006-08-27	2006-09-28			Entanglement		Released
586	Caretta caretta	2006	2006-09-01	2009-10-06			Entanglement		Released
587	Caretta caretta	2006	2006-09-06	2006-10-12			Hooks/monofilament lines		Released
588	Caretta caretta	2006	2006-09-07	2006-09-19			trauma		Released
589	Caretta caretta	2006	2006-09-07	2006-09-28			Entanglement		Released
590	Caretta caretta	2006	2006-09-09	2006-09-10			Infectious disease		Died during hospitalization
591	Caretta caretta	2006	2006-09-10	2006-09-26			Infectious disease		Died during hospitalization
592	Caretta caretta	2006	2006-09-10	2006-09-28			Entanglement		Released
593	Caretta caretta	2006	2006-09-18	2006-10-06			Entanglement		Released
594	Caretta caretta	2006	2006-09-19	2006-09-28			Entanglement		Released
595	Caretta caretta	2006	2006-09-22	2006-10-06			Infectious disease		Released
596	Caretta caretta	2006	2006-09-22	2006-10-06			Entanglement		Released
597	Caretta caretta	2006	2006-09-29	2006-09-29			Infectious disease		Died during hospitalization
598	Caretta caretta	2006	2006-10-02	2006-10-06			Entanglement		Released
599	Caretta caretta	2006	2006-10-03	2006-10-21			Unknown/undetermined		Released
600	Caretta caretta	2006	2006-10-06	2006-10-21			Entanglement		Released
601	Caretta caretta	2006	2006-10-14	2006-10-21			Entanglement		Released
602	Caretta caretta	2006	2006-10-24	2006-12-15			Entanglement		Released
603	Caretta caretta	2006	2006-11-16	2006-11-17			Entanglement		Euthanized
604	Caretta caretta	2006	2006-11-28	2006-12-07			Entanglement		Died during hospitalization
605	Caretta caretta	2006	2006-12-01	2007-01-17			Entanglement		Released
606	Caretta caretta	2006	2006-12-04	2007-01-17			Entanglement		Released
607	Caretta caretta	2006	2006-12-11	2007-02-02			Entanglement		Died during hospitalization
608	Caretta caretta	2006	2006-12-18	2006-12-27			Infectious disease		Released
609	Caretta caretta	2006	2006-12-24	2007-01-17			Entanglement		Released
610	Caretta caretta	2007	2007-01-02				Entanglement		Admitted dead
611	Caretta caretta	2007	2007-01-03	2007-04-12			Infectious disease		Released
612	Caretta caretta	2007	2007-01-09	2007-01-24			Hooks/monofilament lines		Died during hospitalization
613	Caretta caretta	2007	2007-02-12	2007-02-16			Infectious disease		Died during hospitalization
614	Caretta caretta	2007	2007-03-06	2007-03-07			Entanglement		Died during hospitalization
615	Caretta caretta	2007	2007-03-11	2007-03-15			Entanglement		Died during hospitalization
616	Caretta caretta	2007	2007-03-19	2007-04-28			Infectious disease		Died during hospitalization
617	Caretta caretta	2007	2007-03-31	2007-04-19			Unknown/undetermined		Released
618	Caretta caretta	2007	2007-04-03	2007-04-19			Infectious disease		Released
619	Caretta caretta	2007	2007-04-05	2007-04-26			Entanglement		Released
620	Caretta caretta	2007	2007-04-12	2007-05-02			Entanglement		Released
621	Caretta caretta	2007	2007-04-15	2007-04-26			Infectious disease		Released
622	Caretta caretta	2007	2007-04-19	2007-04-20			Infectious disease		Died during hospitalization
623	Caretta caretta	2007	2007-04-23	2007-04-26			Entanglement		Died during hospitalization
624	Caretta caretta	2007	2007-04-26	2007-06-04			Infectious disease		Died during hospitalization
625	Caretta caretta	2007	2007-05-09	2007-05-17			Infectious disease		Released
626	Caretta caretta	2007	2007-05-17	2007-06-04			Infectious disease		Euthanized
627	Caretta caretta	2007	2007-05-19	2007-07-01			Infectious disease		Released
628	Caretta caretta	2007	2007-05-24	2007-06-05			Infectious disease		Died during hospitalization
629	Caretta caretta	2007	2007-06-02	2007-06-10			Infectious disease		Released
630	Caretta caretta	2007	2007-06-02	2007-06-10			Entanglement		Released
631	Caretta caretta	2007	2007-06-11	2007-07-01			Entanglement		Released
632	Caretta caretta	2007	2007-06-12	2007-07-01			Infectious disease		Released
633	Caretta caretta	2007	2007-06-16	2007-06-28			Entanglement		Died during hospitalization
634	Caretta caretta	2007	2007-06-21	2007-07-01			Entanglement		Released
635	Caretta caretta	2007	2007-06-25				Entanglement		Admitted dead
636	Caretta caretta	2007	2007-06-30	2007-07-13			Entanglement		Released
637	Caretta caretta	2007	2007-07-01	2007-07-25			Hooks/monofilament lines		Released
638	Caretta caretta	2007	2007-07-01	2007-07-01			other causes	Shark attack	Euthanized
639	Caretta caretta	2007	2007-07-02	2007-07-13			Unknown/undetermined		Released
640	Caretta caretta	2007	2007-07-04	2007-07-29			other causes	Shark attack	Released
641	Caretta caretta	2007	2007-07-08	2007-10-17			trauma		Released
642	Caretta caretta	2007	2007-07-08	2007-07-18			Entanglement		Released

Record ID	Species	Year	Admission date	Departure date	SCL (cm)	Body weight (kg)	Cause of admission	Other-cause subcategory	Final disposition
643	Caretta caretta	2007	2007-07-08	2007-07-18			Entanglement		Released
644	Caretta caretta	2007	2007-07-10	2007-07-29			Entanglement		Released
645	Caretta caretta	2007	2007-07-11	2007-07-14			Hooks/monofilament lines		Died during hospitalization
646	Caretta caretta	2007	2007-07-12	2007-07-29			Entanglement		Released
647	Caretta caretta	2007	2007-07-20	2007-10-23			Entanglement		Released
648	Caretta caretta	2007	2007-07-27	2007-08-18			Entanglement		Released
649	Caretta caretta	2007	2007-07-28	2007-08-24			Entanglement		Released
650	Caretta caretta	2007	2007-07-30	2007-07-30			Hooks/monofilament lines		Euthanized
651	Caretta caretta	2007	2007-07-31	2007-08-18			Hooks/monofilament lines		Released
652	Caretta caretta	2007	2007-08-03	2007-08-11			Infectious disease		Released
653	Caretta caretta	2007	2007-08-05				Entanglement		Admitted dead
654	Caretta caretta	2007	2007-08-09	2007-09-06			Entanglement		Released
655	Caretta caretta	2007	2007-08-13	2007-08-25			Entanglement		Released
656	Caretta caretta	2007	2007-08-13	2007-09-06			Entanglement		Released
657	Caretta caretta	2007	2007-08-14	2007-08-24			Unknown/undetermined		Released
658	Caretta caretta	2007	2007-08-14	2007-08-14			Entanglement		Released
659	Caretta caretta	2007	2007-08-15	2007-08-25			Hooks/monofilament lines		Released
660	Caretta caretta	2007	2007-08-16	2007-08-25			Entanglement		Released
661	Caretta caretta	2007	2007-08-16	2007-09-15			Entanglement		Released
662	Caretta caretta	2007	2007-08-20	2007-09-06			Entanglement		Released
663	Caretta caretta	2007	2007-08-22	2007-09-06			Entanglement		Released
664	Caretta caretta	2007	2007-08-24	2007-09-15			Entanglement		Released
665	Caretta caretta	2007	2007-08-27	2007-09-15			Entanglement		Released
666	Caretta caretta	2007	2007-08-29	2007-09-15			Hooks/monofilament lines		Released
667	Caretta caretta	2007	2007-08-30	2007-10-23			Entanglement		Released
668	Caretta caretta	2007	2007-09-01	2007-09-29			Hooks/monofilament lines		Released
669	Caretta caretta	2007	2007-09-03	2007-10-05			Entanglement		Released
670	Caretta caretta	2007	2007-09-04	2007-09-15			Entanglement		Released
671	Caretta caretta	2007	2007-09-07	2007-09-22			Infectious disease		Released
672	Caretta caretta	2007	2007-09-07	2007-09-22			Entanglement		Released
673	Caretta caretta	2007	2007-09-10	2007-10-30			Hooks/monofilament lines		Released
674	Caretta caretta	2007	2007-09-10	2007-09-22			Entanglement		Released
675	Caretta caretta	2007	2007-09-10	2007-10-10			Entanglement		Released
676	Caretta caretta	2007	2007-09-10	2007-10-02			Entanglement		Died during hospitalization
677	Caretta caretta	2007	2007-09-11	2007-10-10			Entanglement		Released
678	Caretta caretta	2007	2007-09-11	2007-09-16			Entanglement		Died during hospitalization
679	Caretta caretta	2007	2007-09-12	2007-09-18			Infectious disease		Died during hospitalization
680	Caretta caretta	2007	2007-09-15	2007-09-25			Infectious disease		Released
681	Caretta caretta	2007	2007-09-16	2007-10-05			Entanglement		Released
682	Caretta caretta	2007	2007-09-16	2007-10-05			Entanglement		Released
683	Caretta caretta	2007	2007-09-21	2007-10-05			Entanglement		Released
684	Caretta caretta	2007	2007-09-21	2007-10-18			Entanglement		Released
685	Caretta caretta	2007	2007-09-21	2007-11-13			Entanglement		Released
686	Caretta caretta	2007	2007-09-21	2008-03-25			Entanglement		Released
687	Caretta caretta	2007	2007-09-27	2007-10-06			Infectious disease		Died during hospitalization
688	Caretta caretta	2007	2007-09-28	2007-10-18			Entanglement		Released
689	Caretta caretta	2007	2007-09-29	2007-10-18			Entanglement		Released
690	Caretta caretta	2007	2007-09-30	2007-10-18			Entanglement		Released
691	Caretta caretta	2007	2007-10-03	2007-10-23			other causes	Malnutrition	Released
692	Caretta caretta	2007	2007-10-06	2007-10-25			other causes	Malnutrition	Released
693	Caretta caretta	2007	2007-10-07	2007-10-25			Entanglement		Released
694	Caretta caretta	2007	2007-10-09	2007-11-13			Entanglement		Released
695	Caretta caretta	2007	2007-10-10	2007-10-13			other causes	Malnutrition	Died during hospitalization
696	Caretta caretta	2007	2007-10-14	2007-10-16			Entanglement		Euthanized
697	Caretta caretta	2007	2007-10-14	2007-11-08			Entanglement		Released
698	Caretta caretta	2007	2007-10-15	2007-10-30			Entanglement		Released
699	Caretta caretta	2007	2007-10-20	2007-11-08			Entanglement		Released
700	Caretta caretta	2007	2007-10-22	2007-10-25			Entanglement		Died during hospitalization
701	Caretta caretta	2007	2007-11-04	2008-03-25			Entanglement		Released
702	Caretta caretta	2007	2007-11-30				Hooks/monofilament lines		Admitted dead
703	Caretta caretta	2007	2007-12-10	2008-04-02			Entanglement		Released
704	Caretta caretta	2007	2007-12-26	2008-04-12			Entanglement		Released
705	Caretta caretta	2008	2008-01-22				Entanglement		Admitted dead
706	Caretta caretta	2008	2008-02-04	2008-05-13			Hooks/monofilament lines		Released
707	Caretta caretta	2008	2008-02-04	2008-04-26			Entanglement		Released
708	Caretta caretta	2008	2008-02-21	2008-04-02			Entanglement		Released
709	Caretta caretta	2008	2008-03-05	2008-05-29			Entanglement		Died during hospitalization
710	Caretta caretta	2008	2008-03-07	2008-05-27			Entanglement		Released
711	Caretta caretta	2008	2008-03-10	2008-04-26			Entanglement		Released
712	Caretta caretta	2008	2008-03-18				Crude oil		Admitted dead
713	Caretta caretta	2008	2008-03-19	2008-04-11			Crude oil		Released
714	Caretta caretta	2008	2008-03-23	2008-04-12			Crude oil		Released

Record ID	Species	Year	Admission date	Departure date	SCL (cm)	Body weight (kg)	Cause of admission	Other-cause subcategory	Final disposition
715	Caretta caretta	2008	2008-03-23	2008-05-13			Entanglement		Released
716	Caretta caretta	2008	2008-03-31				Entanglement		Admitted dead
717	Caretta caretta	2008	2008-04-12	2008-04-15			Entanglement		Euthanized
718	Caretta caretta	2008	2008-04-16	2008-05-20			Entanglement		Released
719	Caretta caretta	2008	2008-04-16	2008-06-17			Entanglement		Released
720	Caretta caretta	2008	2008-04-23	2008-04-23			Entanglement		Euthanized
721	Caretta caretta	2008	2008-04-26	2008-06-17			Entanglement		Released
722	Caretta caretta	2008	2008-04-28	2008-05-08			Entanglement		Released
723	Caretta caretta	2008	2008-04-30				Entanglement		Admitted dead
724	Caretta caretta	2008	2008-05-01	2008-06-22			Entanglement		Released
725	Caretta caretta	2008	2008-05-03	2008-05-17			Infectious disease		Released
726	Caretta caretta	2008	2008-05-03	2008-05-17			Entanglement		Released
727	Caretta caretta	2008	2008-05-04				Entanglement		Admitted dead
728	Caretta caretta	2008	2008-05-05	2008-05-20			Crude oil		Released
729	Caretta caretta	2008	2008-05-05	2008-06-22			Crude oil		Released
730	Caretta caretta	2008	2008-05-05	2008-05-15			Entanglement		Released
731	Caretta caretta	2008	2008-05-05				Entanglement		Admitted dead
732	Caretta caretta	2008	2008-05-11	2008-06-03			Entanglement		Released
733	Caretta caretta	2008	2008-05-11	2008-07-05			Entanglement		Released
734	Caretta caretta	2008	2008-05-14	2008-07-10			Crude oil		Released
735	Caretta caretta	2008	2008-05-14	2008-09-06			Crude oil		Released
736	Caretta caretta	2008	2008-05-14	2008-05-14			Entanglement		Euthanized
737	Caretta caretta	2008	2008-05-14	2008-05-27			Entanglement		Released
738	Caretta caretta	2008	2008-05-14				Entanglement		Admitted dead
739	Caretta caretta	2008	2008-05-17	2008-06-22			Entanglement		Released
740	Caretta caretta	2008	2008-05-18				Entanglement		Admitted dead
741	Caretta caretta	2008	2008-05-19	2008-06-03			Entanglement		Released
742	Caretta caretta	2008	2008-05-20	2008-07-10			Infectious disease		Released
743	Caretta caretta	2008	2008-05-21	2008-07-05			Entanglement		Released
744	Caretta caretta	2008	2008-05-23				Entanglement		Admitted dead
745	Caretta caretta	2008	2008-05-30				trauma		Admitted dead
746	Caretta caretta	2008	2008-06-06	2008-06-18			Entanglement		Released
747	Caretta caretta	2008	2008-06-07				Entanglement		Admitted dead
748	Caretta caretta	2008	2008-06-11	2008-08-16			Entanglement		Released
749	Caretta caretta	2008	2008-06-13	2008-07-19			Entanglement		Released
750	Caretta caretta	2008	2008-06-14	2008-06-22			Entanglement		Released
751	Caretta caretta	2008	2008-06-16	2008-07-10			Entanglement		Released
752	Caretta caretta	2008	2008-06-19	2008-07-05			Entanglement		Released
753	Caretta caretta	2008	2008-06-19				Entanglement		Admitted dead
754	Caretta caretta	2008	2008-06-22	2008-06-23			Infectious disease		Died during hospitalization
755	Caretta caretta	2008	2008-06-22	2008-08-30			Entanglement		Released
756	Caretta caretta	2008	2008-06-24	2008-06-25			Entanglement		Died during hospitalization
757	Caretta caretta	2008	2008-06-25	2008-08-30			Entanglement		Released
758	Caretta caretta	2008	2008-06-28	2008-07-19			Entanglement		Released
759	Caretta caretta	2008	2008-06-28	2008-07-19			Entanglement		Released
760	Caretta caretta	2008	2008-06-29	2008-06-30			Entanglement		Euthanized
761	Caretta caretta	2008	2008-07-03	2008-07-18			Entanglement		Released
762	Caretta caretta	2008	2008-07-03	2008-07-19			Entanglement		Released
763	Caretta caretta	2008	2008-07-04	2008-08-16			Entanglement		Released
764	Caretta caretta	2008	2008-07-08	2008-07-18			Entanglement		Released
765	Caretta caretta	2008	2008-07-12	2008-08-02			Entanglement		Released
766	Caretta caretta	2008	2008-07-12	2008-08-16			Entanglement		Released
767	Caretta caretta	2008	2008-07-13	2008-08-16			Entanglement		Released
768	Caretta caretta	2008	2008-07-18				Entanglement		Admitted dead
769	Caretta caretta	2008	2008-07-23	2008-07-24			Hooks/monofilament lines		Died during hospitalization
770	Caretta caretta	2008	2008-07-24				Unknown/undetermined		Admitted dead
771	Caretta caretta	2008	2008-07-26	2008-08-30			Entanglement		Released
772	Caretta caretta	2008	2008-07-27	2008-08-02			Entanglement		Released
773	Caretta caretta	2008	2008-08-04	2008-08-30			Infectious disease		Released
774	Caretta caretta	2008	2008-08-05	2008-09-07			Entanglement		Released
775	Caretta caretta	2008	2008-08-07	2008-08-07			Entanglement		Released
776	Caretta caretta	2008	2008-08-19				Unknown/undetermined		Admitted dead
777	Caretta caretta	2008	2008-08-19	2008-09-07			Entanglement		Released
778	Caretta caretta	2008	2008-08-20	2008-09-06			Entanglement		Released
779	Caretta caretta	2008	2008-08-25	2008-08-30			Entanglement		Released
780	Caretta caretta	2008	2008-08-28				Unknown/undetermined		Admitted dead
781	Caretta caretta	2008	2008-08-28	2008-09-28			Entanglement		Released
782	Caretta caretta	2008	2008-08-28				Entanglement		Admitted dead
783	Caretta caretta	2008	2008-08-29				Unknown/undetermined		Admitted dead
784	Caretta caretta	2008	2008-08-30	2008-09-20			Entanglement		Released
785	Caretta caretta	2008	2008-09-08	2009-02-17			Entanglement		Released
786	Caretta caretta	2008	2008-09-08				Entanglement		Admitted dead

Record ID	Species	Year	Admission date	Departure date	SCL (cm)	Body weight (kg)	Cause of admission	Other-cause subcategory	Final disposition
787	Caretta caretta	2008	2008-09-09				Unknown/undetermined		Admitted dead
788	Caretta caretta	2008	2008-09-19	2009-04-06			other causes	Malnutrition	Released
789	Caretta caretta	2008	2008-10-18	2009-01-31			Entanglement		Released
790	Caretta caretta	2008	2008-10-18	2008-12-16	66	47	Entanglement		Released
791	Caretta caretta	2008	2008-10-24				trauma		Admitted dead
792	Caretta caretta	2008	2008-12-01	2009-01-31			Entanglement		Released
793	Caretta caretta	2008	2008-12-07				Entanglement		Admitted dead
794	Caretta caretta	2008	2008-12-09	2008-12-15			Entanglement		Died during hospitalization
795	Caretta caretta	2008	2008-12-26	2009-02-17			Unknown/undetermined		Released
796	Caretta caretta	2009	2009-01-12	2010-03-03			trauma		Released
797	Caretta caretta	2009	2009-02-14	2009-02-27			trauma		Died during hospitalization
798	Caretta caretta	2009	2009-03-07	2009-06-04			Crude oil		Released
799	Caretta caretta	2009	2009-03-14	2009-06-04			Infectious disease		Released
800	Caretta caretta	2009	2009-03-21				trauma		Admitted dead
801	Caretta caretta	2009	2009-03-21				Unknown/undetermined		Admitted dead
802	Caretta caretta	2009	2009-03-26	2009-06-21			Entanglement		Released
803	Caretta caretta	2009	2009-03-29	2009-03-29			trauma		Euthanized
804	Caretta caretta	2009	2009-04-01	2009-04-11			Entanglement		Released
805	Caretta caretta	2009	2009-05-02	2009-06-21			Entanglement		Released
806	Caretta caretta	2009	2009-05-08	2009-06-23			Entanglement		Released
807	Caretta caretta	2009	2009-05-10	2009-06-04			Entanglement		Released
808	Caretta caretta	2009	2009-05-10	2009-06-15			Entanglement		Released
809	Caretta caretta	2009	2009-05-13	2009-06-17			Infectious disease		Died during hospitalization
810	Caretta caretta	2009	2009-05-15				Unknown/undetermined		Admitted dead
811	Caretta caretta	2009	2009-05-16	2009-07-12			Entanglement		Released
812	Caretta caretta	2009	2009-05-17	2009-06-04			Entanglement		Released
813	Caretta caretta	2009	2009-05-17	2009-07-01			Entanglement		Released
814	Caretta caretta	2009	2009-05-20	2009-06-21			Infectious disease		Released
815	Caretta caretta	2009	2009-05-20	2009-05-29			Entanglement		Died during hospitalization
816	Caretta caretta	2009	2009-06-02	2009-06-24			Entanglement		Released
817	Caretta caretta	2009	2009-06-07	2009-07-12			Entanglement		Released
818	Caretta caretta	2009	2009-06-09	2009-09-05			Unknown/undetermined		Released
819	Caretta caretta	2009	2009-06-13	2009-07-04			Entanglement		Released
820	Caretta caretta	2009	2009-06-13	2009-06-25			Entanglement		Died during hospitalization
821	Caretta caretta	2009	2009-06-14				Unknown/undetermined		Admitted dead
822	Caretta caretta	2009	2009-06-14				Unknown/undetermined		Admitted dead
823	Caretta caretta	2009	2009-06-22	2009-09-18			Entanglement		Released
824	Caretta caretta	2009	2009-06-25	2009-07-15			Entanglement		Released
825	Caretta caretta	2009	2009-07-01				Entanglement		Admitted dead
826	Caretta caretta	2009	2009-07-06	2009-07-23			Entanglement		Released
827	Caretta caretta	2009	2009-07-14	2009-08-01			Entanglement		Released
828	Caretta caretta	2009	2009-07-19	2009-08-29			Entanglement		Released
829	Caretta caretta	2009	2009-07-29	2009-08-29			Entanglement		Released
830	Caretta caretta	2009	2009-07-31	2009-08-21			Entanglement		Released
831	Caretta caretta	2009	2009-08-05				Entanglement		Admitted dead
832	Caretta caretta	2009	2009-08-06	2009-08-30			Entanglement		Released
833	Caretta caretta	2009	2009-08-08	2009-08-21			Entanglement		Released
834	Caretta caretta	2009	2009-08-09	2009-08-10			Infectious disease		Died during hospitalization
835	Caretta caretta	2009	2009-08-15	2009-09-05			Unknown/undetermined		Released
836	Caretta caretta	2009	2009-08-16				Unknown/undetermined		Admitted dead
837	Caretta caretta	2009	2009-08-17	2009-09-05			Entanglement		Released
838	Caretta caretta	2009	2009-08-17	2009-09-26			Entanglement		Released
839	Caretta caretta	2009	2009-08-20	2009-09-11			Entanglement		Released
840	Caretta caretta	2009	2009-08-23	2009-09-11			Entanglement		Released
841	Caretta caretta	2009	2009-08-25	2009-12-03			Entanglement		Released
842	Caretta caretta	2009	2009-08-26	2009-09-05			Entanglement		Released
843	Caretta caretta	2009	2009-08-31	2009-09-11			Entanglement		Released
844	Caretta caretta	2009	2009-08-31	2009-09-18			Entanglement		Released
845	Caretta caretta	2009	2009-08-31	2009-10-09			Entanglement		Released
846	Caretta caretta	2009	2009-09-02	2009-09-18			Entanglement		Released
847	Caretta caretta	2009	2009-09-07	2009-09-26			Entanglement		Released
848	Caretta caretta	2009	2009-09-08	2009-09-18			Entanglement		Released
849	Caretta caretta	2009	2009-09-09	2009-10-09			Entanglement		Released
850	Caretta caretta	2009	2009-09-10	2009-09-11			Entanglement		Released
851	Caretta caretta	2009	2009-09-10	2009-10-03			Entanglement		Released
852	Caretta caretta	2009	2009-09-11	2009-09-26			Entanglement		Released
853	Caretta caretta	2009	2009-09-11	2009-10-24			Entanglement		Released
854	Caretta caretta	2009	2009-09-12	2009-10-03			Entanglement		Released
855	Caretta caretta	2009	2009-09-12	2009-11-04			Entanglement		Released
856	Caretta caretta	2009	2009-09-13	2009-09-25			Entanglement		Released
857	Caretta caretta	2009	2009-09-14	2009-09-26			Entanglement		Released
858	Caretta caretta	2009	2009-09-18	2009-10-03			Entanglement		Released

Record ID	Species	Year	Admission date	Departure date	SCL (cm)	Body weight (kg)	Cause of admission	Other-cause subcategory	Final disposition
859	Caretta caretta	2009	2009-09-23	2009-10-24			Entanglement		Released
860	Caretta caretta	2009	2009-10-03	2009-11-07			Entanglement		Released
861	Caretta caretta	2009	2009-10-04	2009-10-24			Entanglement		Released
862	Caretta caretta	2009	2009-10-04	2009-11-07			Entanglement		Released
863	Caretta caretta	2009	2009-10-05	2009-10-13			Entanglement		Died during hospitalization
864	Caretta caretta	2009	2009-10-09	2009-12-19			Unknown/undetermined		Released
865	Caretta caretta	2009	2009-10-11	2009-11-07			Hooks/monofilament lines		Released
866	Caretta caretta	2009	2009-10-11				Entanglement		Admitted dead
867	Caretta caretta	2009	2009-10-19	2009-11-04			Entanglement		Released
868	Caretta caretta	2009	2009-10-19	2009-12-19			Entanglement		Released
869	Caretta caretta	2009	2009-10-23				Unknown/undetermined		Admitted dead
870	Caretta caretta	2009	2009-10-26	2009-11-14			Entanglement		Released
871	Caretta caretta	2009	2009-11-21	2010-02-05			Hooks/monofilament lines		Released
872	Caretta caretta	2009	2009-11-25				Unknown/undetermined		Admitted dead
873	Caretta caretta	2009	2009-12-11				Unknown/undetermined		Admitted dead
874	Caretta caretta	2009	2009-12-19				Unknown/undetermined		Admitted dead
875	Caretta caretta	2010	2010-01-12	2010-01-16			Entanglement		Died during hospitalization
876	Caretta caretta	2010	2010-01-30	2010-03-26			Entanglement		Released
877	Caretta caretta	2010	2010-01-30	2010-03-26			Entanglement		Released
878	Caretta caretta	2010	2010-02-21	2010-04-26			Entanglement		Released
879	Caretta caretta	2010	2010-03-13	2010-05-19			Entanglement		Died during hospitalization
880	Caretta caretta	2010	2010-03-18	2010-06-05			Entanglement		Released
881	Caretta caretta	2010	2010-03-27	2010-07-15			Entanglement		Released
882	Caretta caretta	2010	2010-04-03	2010-04-26			Entanglement		Released
883	Caretta caretta	2010	2010-04-03	2010-06-06			Entanglement		Released
884	Caretta caretta	2010	2010-04-09	2010-04-10			other causes	Malnutrition	Died during hospitalization
885	Caretta caretta	2010	2010-04-27				Infectious disease		Admitted dead
886	Caretta caretta	2010	2010-05-16				Unknown/undetermined		Admitted dead
887	Caretta caretta	2010	2010-06-03	2010-06-10			other causes	Malnutrition	Died during hospitalization
888	Caretta caretta	2010	2010-06-06	2010-07-15			Entanglement		Released
889	Caretta caretta	2010	2010-06-09	2010-07-17			Hooks/monofilament lines		Released
890	Caretta caretta	2010	2010-06-17	2010-10-01			Entanglement		Released
891	Caretta caretta	2010	2010-06-21	2010-06-24			Infectious disease		Died during hospitalization
892	Caretta caretta	2010	2010-06-23	2010-07-17			Entanglement		Released
893	Caretta caretta	2010	2010-06-23	2010-08-06			Entanglement		Released
894	Caretta caretta	2010	2010-07-06	2010-09-04			Entanglement		Released
895	Caretta caretta	2010	2010-07-09				Unknown/undetermined		Admitted dead
896	Caretta caretta	2010	2010-07-10	2010-08-06			Unknown/undetermined		Released
897	Caretta caretta	2010	2010-07-21	2010-08-06			Infectious disease		Released
898	Caretta caretta	2010	2010-07-22	2010-08-06			Entanglement		Released
899	Caretta caretta	2010	2010-07-22				Entanglement		Admitted dead
900	Caretta caretta	2010	2010-07-24	2012-09-05			Infectious disease		Died during hospitalization
901	Caretta caretta	2010	2010-07-24	2010-10-29			Entanglement		Released
902	Caretta caretta	2010	2010-07-25	2010-07-26			Infectious disease		Died during hospitalization
903	Caretta caretta	2010	2010-07-27	2010-08-14			other causes	Malnutrition	Released
904	Caretta caretta	2010	2010-07-27	2010-08-14			other causes	Malnutrition	Released
905	Caretta caretta	2010	2010-07-27				Unknown/undetermined		Admitted dead
906	Caretta caretta	2010	2010-07-28	2010-09-18			Entanglement		Released
907	Caretta caretta	2010	2010-08-07	2010-08-21			other causes	Malnutrition	Released
908	Caretta caretta	2010	2010-08-07	2010-08-21			Entanglement		Released
909	Caretta caretta	2010	2010-08-17				Entanglement		Admitted dead
910	Caretta caretta	2010	2010-08-20	2010-11-02			Entanglement		Released
911	Caretta caretta	2010	2010-08-21	2010-12-02			Unknown/undetermined		Released
912	Caretta caretta	2010	2010-08-22				Entanglement		Admitted dead
913	Caretta caretta	2010	2010-08-23	2010-09-13			Infectious disease		Died during hospitalization
914	Caretta caretta	2010	2010-08-27	2010-09-19			Unknown/undetermined		Released
915	Caretta caretta	2010	2010-08-27				Unknown/undetermined		Admitted dead
916	Caretta caretta	2010	2010-08-27				Unknown/undetermined		Admitted dead
917	Caretta caretta	2010	2010-09-08				Unknown/undetermined		Admitted dead
918	Caretta caretta	2010	2010-09-10	2010-09-10			Hooks/monofilament lines		Died during hospitalization
919	Caretta caretta	2010	2010-09-13	2010-09-19			Entanglement		Released
920	Caretta caretta	2010	2010-09-18				Unknown/undetermined		Admitted dead
921	Caretta caretta	2010	2010-09-28				Unknown/undetermined		Admitted dead
922	Caretta caretta	2010	2010-09-28	2010-10-29			Entanglement		Released
923	Caretta caretta	2010	2010-09-28	2010-11-02			Entanglement		Released
924	Caretta caretta	2010	2010-09-29	2010-10-29			Entanglement		Released
925	Caretta caretta	2010	2010-10-06	2010-10-18			Hooks/monofilament lines		Released
926	Caretta caretta	2010	2010-10-21	2010-12-15			Entanglement		Released
927	Caretta caretta	2010	2010-11-14	2010-11-17			Infectious disease		Released
928	Caretta caretta	2010	2010-11-15	2010-11-26			Entanglement		Died during hospitalization
929	Caretta caretta	2010	2010-12-02	2010-12-29			Infectious disease		Released
930	Caretta caretta	2011	2011-01-11	2011-02-01	46	14.2	trauma		Released

Record ID	Species	Year	Admission date	Departure date	SCL (cm)	Body weight (kg)	Cause of admission	Other-cause subcategory	Final disposition
931	Caretta caretta	2011	2011-02-06	2011-02-07			Hooks/monofilament lines		Died during hospitalization
932	Caretta caretta	2011	2011-04-01	2011-04-20			Entanglement		Died during hospitalization
933	Caretta caretta	2011	2011-04-11				Crude oil		Admitted dead
934	Caretta caretta	2011	2011-04-21	2011-05-04			Entanglement		Died during hospitalization
935	Caretta caretta	2011	2011-04-21				Entanglement		Admitted dead
936	Caretta caretta	2011	2011-04-26	2011-07-25			Entanglement		Released
937	Caretta caretta	2011	2011-04-27	2011-05-02			trauma		Died during hospitalization
938	Caretta caretta	2011	2011-05-11	2011-06-08			Entanglement		Released
939	Caretta caretta	2011	2011-05-30	2011-05-31			trauma		Died during hospitalization
940	Caretta caretta	2011	2011-06-03				Entanglement		Admitted dead
941	Caretta caretta	2011	2011-06-27				Hooks/monofilament lines		Admitted dead
942	Caretta caretta	2011	2011-07-07	2011-07-25			Entanglement		Released
943	Caretta caretta	2011	2011-07-11	2011-09-03			Entanglement		Released
944	Caretta caretta	2011	2011-07-14				Unknown/undetermined		Admitted dead
945	Caretta caretta	2011	2011-07-15				Unknown/undetermined		Admitted dead
946	Caretta caretta	2011	2011-07-15				Unknown/undetermined		Admitted dead
947	Caretta caretta	2011	2011-07-18				Entanglement		Admitted dead
948	Caretta caretta	2011	2011-07-20				Unknown/undetermined		Admitted dead
949	Caretta caretta	2011	2011-07-21	2011-09-24			Entanglement		Released
950	Caretta caretta	2011	2011-07-21	2011-07-28			Entanglement		Died during hospitalization
951	Caretta caretta	2011	2011-07-21	2011-09-15			Entanglement		Died during hospitalization
952	Caretta caretta	2011	2011-07-24	2011-08-02			Hooks/monofilament lines		Died during hospitalization
953	Caretta caretta	2011	2011-07-25				Unknown/undetermined		Admitted dead
954	Caretta caretta	2011	2011-08-08	2011-09-03			Entanglement		Released
955	Caretta caretta	2011	2011-08-21				Unknown/undetermined		Admitted dead
956	Caretta caretta	2011	2011-08-23	2011-09-20			Infectious disease		Died during hospitalization
957	Caretta caretta	2011	2011-08-29				Entanglement		Admitted dead
958	Caretta caretta	2011	2011-09-08	2011-09-24			Entanglement		Released
959	Caretta caretta	2011	2011-09-09	2011-11-30			Entanglement		Released
960	Caretta caretta	2011	2011-09-11	2011-09-13			Infectious disease		Died during hospitalization
961	Caretta caretta	2011	2011-09-11	2011-10-10			Entanglement		Released
962	Caretta caretta	2011	2011-09-13				Unknown/undetermined		Admitted dead
963	Caretta caretta	2011	2011-09-19	2011-10-22			Unknown/undetermined		Died during hospitalization
964	Caretta caretta	2011	2011-09-19	2011-09-29			Infectious disease		Died during hospitalization
965	Caretta caretta	2011	2011-09-27	2011-12-20			Unknown/undetermined		Released
966	Caretta caretta	2011	2011-09-27	2011-10-10			Entanglement		Released
967	Caretta caretta	2011	2011-09-30	2011-11-30			Entanglement		Released
968	Caretta caretta	2011	2011-10-03	2011-11-17			Entanglement		Released
969	Caretta caretta	2011	2011-10-14				other causes	Malnutrition	Admitted dead
970	Caretta caretta	2011	2011-10-16				Unknown/undetermined		Admitted dead
971	Caretta caretta	2011	2011-10-23	2011-11-17	31.5	2.7	Infectious disease		Released
972	Caretta caretta	2011	2011-10-31	2011-12-20	41	11	Entanglement		Released
973	Caretta caretta	2011	2011-11-07	2012-02-24	34	5.5	Entanglement		Released
974	Caretta caretta	2011	2011-11-09	2011-11-30			Entanglement		Released
975	Caretta caretta	2011	2011-11-14	2011-11-25			Entanglement		Died during hospitalization
976	Caretta caretta	2011	2011-11-14				Entanglement		Admitted dead
977	Caretta caretta	2011	2011-12-03	2012-01-28			Entanglement		Released
978	Caretta caretta	2011	2011-12-03				Entanglement		Admitted dead
979	Caretta caretta	2011	2011-12-11	2012-01-28			Entanglement		Released
980	Caretta caretta	2011	2011-12-15	2012-03-07	34		Entanglement		Released
981	Caretta caretta	2011	2011-12-20				Hooks/monofilament lines		Admitted dead
982	Caretta caretta	2011	2011-12-22				Unknown/undetermined		Admitted dead
983	Caretta caretta	2011	2011-12-29				Unknown/undetermined		Admitted dead
984	Caretta caretta	2012	2012-01-24	2012-05-03			Unknown/undetermined		Released
985	Caretta caretta	2012	2012-01-28	2012-03-21	27		Unknown/undetermined		Died during hospitalization
986	Caretta caretta	2012	2012-01-30	2012-03-07			Entanglement		Released
987	Caretta caretta	2012	2012-02-04	2012-02-24	19.5	1.2	Entanglement		Released
988	Caretta caretta	2012	2012-02-14	2012-03-07	33	5	Entanglement		Released
989	Caretta caretta	2012	2012-02-20	2012-03-17	48	17.5	Entanglement		Released
990	Caretta caretta	2012	2012-02-22	2012-03-17	29.8	4.7	Entanglement		Released
991	Caretta caretta	2012	2012-02-22	2012-02-25	28	3.4	Entanglement		Died during hospitalization
992	Caretta caretta	2012	2012-02-24	2012-03-30	27.2	3.3	Entanglement		Released
993	Caretta caretta	2012	2012-02-25	2012-03-17	36.5	9	Unknown/undetermined		Released
994	Caretta caretta	2012	2012-02-25	2012-06-08	53	21	Entanglement		Released
995	Caretta caretta	2012	2012-03-01	2012-03-24	25	2.4	Entanglement		Released
996	Caretta caretta	2012	2012-03-01	2012-03-30	34.5	5.4	Entanglement		Released
997	Caretta caretta	2012	2012-03-04	2012-03-30	22.5	2.1	Unknown/undetermined		Released
998	Caretta caretta	2012	2012-03-15	2012-06-05	25.8	2.8	Entanglement		Released
999	Caretta caretta	2012	2012-03-18	2012-03-24	36.5		Unknown/undetermined		Released
1000	Caretta caretta	2012	2012-03-25		35		Unknown/undetermined		Admitted dead
1001	Caretta caretta	2012	2012-03-25	2012-05-03	31.5		Entanglement		Released
1002	Caretta caretta	2012	2012-03-31				Unknown/undetermined		Admitted dead

Record ID	Species	Year	Admission date	Departure date	SCL (cm)	Body weight (kg)	Cause of admission	Other-cause subcategory	Final disposition
1003	Caretta caretta	2012	2012-04-24	2012-06-08	32.5	6.1	Entanglement		Released
1004	Caretta caretta	2012	2012-05-01	2012-05-07	84	97	trauma		Released
1005	Caretta caretta	2012	2012-05-11	2012-06-05	33	5.8	Entanglement		Released
1006	Caretta caretta	2012	2012-05-16	2012-06-08	40		Unknown/undetermined		Released
1007	Caretta caretta	2012	2012-05-21		22	1.8	Unknown/undetermined		Admitted dead
1008	Caretta caretta	2012	2012-05-29				Unknown/undetermined		Admitted dead
1009	Caretta caretta	2012	2012-06-01	2012-07-23	30.2	4.1	Entanglement		Released
1010	Caretta caretta	2012	2012-06-01	2012-07-23	34	6.5	Entanglement		Released
1011	Caretta caretta	2012	2012-06-03	2012-06-30	42.2	16	Unknown/undetermined		Released
1012	Caretta caretta	2012	2012-06-05	2012-06-30	26	2.5	Entanglement		Released
1013	Caretta caretta	2012	2012-06-06				Unknown/undetermined		Admitted dead
1014	Caretta caretta	2012	2012-06-09	2012-06-30	25.5	3.4	Unknown/undetermined		Released
1015	Caretta caretta	2012	2012-06-28		63		Unknown/undetermined		Admitted dead
1016	Caretta caretta	2012	2012-06-29	2012-07-23	27	3.5	Entanglement		Released
1017	Caretta caretta	2012	2012-07-02	2012-08-22	43	10.6	Entanglement		Released
1018	Caretta caretta	2012	2012-07-04	2012-07-23	40	12	Infectious disease		Released
1019	Caretta caretta	2012	2012-07-07	2012-08-22	35	6.8	Entanglement		Released
1020	Caretta caretta	2012	2012-07-09				Unknown/undetermined		Admitted dead
1021	Caretta caretta	2012	2012-07-12	2012-08-22			Entanglement		Released
1022	Caretta caretta	2012	2012-07-19	2012-08-11	33	5	Entanglement		Released
1023	Caretta caretta	2012	2012-07-24	2012-08-26	53	36.5	Infectious disease		Released
1024	Caretta caretta	2012	2012-07-25	2012-09-08	34	6.2	Entanglement		Released
1025	Caretta caretta	2012	2012-07-27	2012-08-26			Hooks/monofilament lines		Released
1026	Caretta caretta	2012	2012-07-30				Unknown/undetermined		Admitted dead
1027	Caretta caretta	2012	2012-07-30	2012-08-11			Entanglement		Released
1028	Caretta caretta	2012	2012-07-30	2012-08-11			Entanglement		Released
1029	Caretta caretta	2012	2012-07-31	2012-10-09	34		Entanglement		Released
1030	Caretta caretta	2012	2012-08-10	2012-09-08	41.1	12	Entanglement		Released
1031	Caretta caretta	2012	2012-08-12	2012-09-12	35.1	8	other causes	Malnutrition	Died during hospitalization
1032	Caretta caretta	2012	2012-08-15	2012-08-15	46		other causes	Malnutrition	Died during hospitalization
1033	Caretta caretta	2012	2012-08-15				Unknown/undetermined		Admitted dead
1034	Caretta caretta	2012	2012-08-16	2012-08-26			Unknown/undetermined		Released
1035	Caretta caretta	2012	2012-08-19				Unknown/undetermined		Admitted dead
1036	Caretta caretta	2012	2012-08-20	2012-11-30	48	19.2	Unknown/undetermined		Released
1037	Caretta caretta	2012	2012-08-20	2012-09-29			Entanglement		Released
1038	Caretta caretta	2012	2012-08-22	2012-09-02	34.5	7	Entanglement		Released
1039	Caretta caretta	2012	2012-08-28	2012-11-02	33.5	6.8	Unknown/undetermined		Released
1040	Caretta caretta	2012	2012-09-01	2012-09-29	33	5.8	Entanglement		Released
1041	Caretta caretta	2012	2012-09-01	2012-09-29	38.5	10.2	Entanglement		Released
1042	Caretta caretta	2012	2012-09-05	2013-01-15	26.9	3.2	Entanglement		Released
1043	Caretta caretta	2012	2012-09-07	2012-09-07	31	6.4	Unknown/undetermined		Released
1044	Caretta caretta	2012	2012-09-07	2012-10-09			Entanglement		Released
1045	Caretta caretta	2012	2012-09-12	2012-10-10	31	5.7	Entanglement		Released
1046	Caretta caretta	2012	2012-09-19	2012-09-19	37	9.5	Entanglement		Released
1047	Caretta caretta	2012	2012-09-29	2012-10-10	36.5	9.3	Infectious disease		Released
1048	Caretta caretta	2012	2012-10-13	2013-01-15	35	7.5	Entanglement		Released
1049	Caretta caretta	2012	2012-10-15	2012-11-30			Entanglement		Released
1050	Caretta caretta	2012	2012-12-30	2013-01-15	20.5	1.3	Entanglement		Released
1051	Caretta caretta	2013	2013-01-23	2013-05-14	22.7	2	Entanglement		Released
1052	Caretta caretta	2013	2013-02-16	2013-03-22	35	6.8	Entanglement		Released
1053	Caretta caretta	2013	2013-02-17	2013-05-22			Infectious disease		Released
1054	Caretta caretta	2013	2013-03-22				Unknown/undetermined		Admitted dead
1055	Caretta caretta	2013	2013-04-10	2013-04-14	35	6	Entanglement		Released
1056	Caretta caretta	2013	2013-04-27	2013-05-30	50.2	22.2	Unknown/undetermined		Released
1057	Caretta caretta	2013	2013-04-27	2013-06-17	38	8.5	Entanglement		Released
1058	Caretta caretta	2013	2013-04-27	2013-05-30	48	18.3	Entanglement		Released
1059	Caretta caretta	2013	2013-05-17	2013-06-19	38	9.4	Entanglement		Released
1060	Caretta caretta	2013	2013-05-18	2013-05-25	53.4	20.7	Infectious disease		Died during hospitalization
1061	Caretta caretta	2013	2013-05-18	2013-07-05			Crude oil		Released
1062	Caretta caretta	2013	2013-05-20		31	6.4	trauma		Admitted dead
1063	Caretta caretta	2013	2013-05-20	2013-06-19	32	5.1	Entanglement		Released
1064	Caretta caretta	2013	2013-05-20	2013-07-20	33.5	5.7	Entanglement		Released
1065	Caretta caretta	2013	2013-05-23	2013-06-17	24.5	2.4	other causes	Malnutrition	Released
1066	Caretta caretta	2013	2013-05-23	2013-06-28	29.5	4.1	Entanglement		Died during hospitalization
1067	Caretta caretta	2013	2013-05-24	2013-06-17	37.5	7.2	Entanglement		Released
1068	Caretta caretta	2013	2013-06-03	2013-06-19	38.5	7.9	Entanglement		Released
1069	Caretta caretta	2013	2013-06-04	2013-06-06	42	11.2	Hooks/monofilament lines		Died during hospitalization
1070	Caretta caretta	2013	2013-06-06	2013-07-18			other causes	Malnutrition	Released
1071	Caretta caretta	2013	2013-06-12	2013-07-20			Entanglement		Released
1072	Caretta caretta	2013	2013-06-13	2013-06-18			Entanglement		Released
1073	Caretta caretta	2013	2013-06-14				Unknown/undetermined		Admitted dead
1074	Caretta caretta	2013	2013-06-14	2013-07-27			Entanglement		Released

Record ID	Species	Year	Admission date	Departure date	SCL (cm)	Body weight (kg)	Cause of admission	Other-cause subcategory	Final disposition
1075	Caretta caretta	2013	2013-06-19	2013-07-22			Entanglement		Released
1076	Caretta caretta	2013	2013-06-21	2013-06-27			Entanglement		Died during hospitalization
1077	Caretta caretta	2013	2013-06-25	2013-07-30			other causes	Malnutrition	Released
1078	Caretta caretta	2013	2013-06-26	2013-08-07			other causes	Malnutrition	Released
1079	Caretta caretta	2013	2013-06-26	2013-07-12			Entanglement		Released
1080	Caretta caretta	2013	2013-06-28	2013-08-23			other causes	Malnutrition	Released
1081	Caretta caretta	2013	2013-06-29	2013-07-20			Infectious disease		Released
1082	Caretta caretta	2013	2013-07-07				Unknown/undetermined		Admitted dead
1083	Caretta caretta	2013	2013-07-07	2013-07-18	31	6.4	Infectious disease		Released
1084	Caretta caretta	2013	2013-07-07	2013-07-27	37.5	7.9	Infectious disease		Released
1085	Caretta caretta	2013	2013-07-08	2013-07-27			Infectious disease		Released
1086	Caretta caretta	2013	2013-07-13	2013-08-13			Entanglement		Released
1087	Caretta caretta	2013	2013-07-14	2013-07-20			Infectious disease		Released
1088	Caretta caretta	2013	2013-07-15				trauma		Admitted dead
1089	Caretta caretta	2013	2013-07-15	2013-08-15			other causes	Malnutrition	Released
1090	Caretta caretta	2013	2013-07-16	2013-07-20			Entanglement		Released
1091	Caretta caretta	2013	2013-07-16	2013-08-08			Entanglement		Released
1092	Caretta caretta	2013	2013-07-18	2013-08-13			Infectious disease		Released
1093	Caretta caretta	2013	2013-07-18	2013-08-08	32	5.4	Entanglement		Released
1094	Caretta caretta	2013	2013-07-21	2013-07-30	24.5	3.1	Infectious disease		Released
1095	Caretta caretta	2013	2013-07-22	2013-07-22	45.2	14.5	Unknown/undetermined		Released
1096	Caretta caretta	2013	2013-07-22	2013-08-15	21.3	1.8	Entanglement		Released
1097	Caretta caretta	2013	2013-07-26	2013-08-08	29.5	4.1	Entanglement		Released
1098	Caretta caretta	2013	2013-07-28	2013-08-15	18.2	1	Infectious disease		Released
1099	Caretta caretta	2013	2013-07-28	2013-08-13	19.8	1.3	Infectious disease		Released
1100	Caretta caretta	2013	2013-07-30				trauma		Admitted dead
1101	Caretta caretta	2013	2013-07-31	2013-09-18	35.5	6.7	Unknown/undetermined		Released
1102	Caretta caretta	2013	2013-08-01	2013-08-15	27.5	3.9	Entanglement		Released
1103	Caretta caretta	2013	2013-08-04	2013-08-08	40	9.2	Infectious disease		Released
1104	Caretta caretta	2013	2013-08-07	2013-08-13	18.5	1.2	Unknown/undetermined		Released
1105	Caretta caretta	2013	2013-08-12	2013-09-18	42.2	12	Infectious disease		Released
1106	Caretta caretta	2013	2013-08-14	2013-08-23	68	59	other causes	Malnutrition	Released
1107	Caretta caretta	2013	2013-08-15	2013-08-31	29.5	3.7	Entanglement		Released
1108	Caretta caretta	2013	2013-08-18	2013-09-18	34	5.7	Hooks/monofilament lines		Released
1109	Caretta caretta	2013	2013-08-21	2013-08-31	57.5	40	Unknown/undetermined		Released
1110	Caretta caretta	2013	2013-08-22	2013-09-18	35.5	6.7	other causes	Malnutrition	Released
1111	Caretta caretta	2013	2013-08-27	2013-09-19	21.5	1.6	Unknown/undetermined		Released
1112	Caretta caretta	2013	2013-08-29	2013-09-19	26	3	Infectious disease		Released
1113	Caretta caretta	2013	2013-08-30	2013-09-26	30.2	4.2	Entanglement		Released
1114	Caretta caretta	2013	2013-09-03	2013-09-19	18.4	1	other causes	Malnutrition	Released
1115	Caretta caretta	2013	2013-09-03	2013-09-26	41	8.6	Entanglement		Released
1116	Caretta caretta	2013	2013-09-04	2013-09-19	25.5	2.7	Entanglement		Released
1117	Caretta caretta	2013	2013-09-04	2013-09-26	40	8.4	Entanglement		Released
1118	Caretta caretta	2013	2013-09-07	2013-09-07	20	1.5	Infectious disease		Released
1119	Caretta caretta	2013	2013-09-07	2013-09-23	31	5.6	Entanglement		Released
1120	Caretta caretta	2013	2013-09-08	2013-09-23	20	1.5	other causes	Malnutrition	Released
1121	Caretta caretta	2013	2013-09-08	2013-09-23	28.1	3.6	Infectious disease		Released
1122	Caretta caretta	2013	2013-09-08	2013-09-26	32	4.7	Entanglement		Released
1123	Caretta caretta	2013	2013-09-14	2013-09-16	40.4	8.6	Infectious disease		Released
1124	Caretta caretta	2013	2013-09-29	2013-10-30	35	6.5	Entanglement		Released
1125	Caretta caretta	2013	2013-10-05	2013-10-30	26.5	2.9	Entanglement		Released
1126	Caretta caretta	2013	2013-10-10	2013-10-22	34	5	Entanglement		Released
1127	Caretta caretta	2013	2013-10-13	2014-02-07	43.5	9	Infectious disease		Released
1128	Caretta caretta	2013	2013-10-20	2013-10-22	49	20.4	other causes	Miscellany	Released
1129	Caretta caretta	2013	2013-10-27				Unknown/undetermined		Admitted dead
1130	Caretta caretta	2013	2013-11-03	2013-11-27	29	3.5	Entanglement		Released
1131	Caretta caretta	2013	2013-11-07	2013-11-27	50.9	23	Infectious disease		Released
1132	Caretta caretta	2013	2013-11-17	2013-12-05	36	6.4	Entanglement		Released
1133	Caretta caretta	2013	2013-11-24	2013-12-05	39	8.8	Entanglement		Released
1134	Caretta caretta	2014	2014-01-03				Entanglement		Admitted dead
1135	Caretta caretta	2014	2014-01-14	2014-01-19	19.5	0.8	other causes	Malnutrition	Died during hospitalization
1136	Caretta caretta	2014	2014-02-12				trauma		Admitted dead
1137	Caretta caretta	2014	2014-02-19		40	15	Unknown/undetermined		Admitted dead
1138	Caretta caretta	2014	2014-03-14	2014-04-03	39.5	9.5	Infectious disease		Released
1139	Caretta caretta	2014	2014-03-17	2014-04-03	37.5	9.5	other causes	Malnutrition	Released
1140	Caretta caretta	2014	2014-03-22	2014-03-24			Infectious disease		Died during hospitalization
1141	Caretta caretta	2014	2014-03-25	2014-04-14	37.5	9.2	other causes	Malnutrition	Released
1142	Caretta caretta	2014	2014-04-10		37.5	6.5	Unknown/undetermined		Admitted dead
1143	Caretta caretta	2014	2014-04-30	2014-05-28	31	6.4	Unknown/undetermined		Released
1144	Caretta caretta	2014	2014-05-04	2014-05-28	30	4.9	Infectious disease		Released
1145	Caretta caretta	2014	2014-05-17	2014-05-28	22.8	2.2	other causes	Malnutrition	Released
1146	Caretta caretta	2014	2014-05-22	2014-05-23			Entanglement		Died during hospitalization

Record ID	Species	Year	Admission date	Departure date	SCL (cm)	Body weight (kg)	Cause of admission	Other-cause subcategory	Final disposition
1147	Caretta caretta	2014	2014-05-30	2014-06-02	21.8	2	other causes	Malnutrition	Released
1148	Caretta caretta	2014	2014-05-30	2014-07-02	29.5		Entanglement		Released
1149	Caretta caretta	2014	2014-05-31	2014-06-12	34.5	7.5	other causes	Malnutrition	Released
1150	Caretta caretta	2014	2014-06-03	2014-07-22	40.2	10.3	Entanglement		Released
1151	Caretta caretta	2014	2014-06-22	2014-09-11	17.5	0.9	other causes	Malnutrition	Released
1152	Caretta caretta	2014	2014-06-25	2014-07-02	17.5		trauma		Released
1153	Caretta caretta	2014	2014-06-25	2014-07-22	32.8	4.4	Entanglement		Released
1154	Caretta caretta	2014	2014-06-25	2014-08-01	42.3	12.1	Entanglement		Released
1155	Caretta caretta	2014	2014-06-26	2014-09-11	42.8	10.8	Entanglement		Released
1156	Caretta caretta	2014	2014-06-28	2014-07-15	28.5	3.9	Entanglement		Released
1157	Caretta caretta	2014	2014-07-01	2014-08-01	30.5	4	Infectious disease		Released
1158	Caretta caretta	2014	2014-07-04	2017-04-15	43	12.3	Unknown/undetermined		Released
1159	Caretta caretta	2014	2014-07-08	2014-08-07	33	5.2	Entanglement		Released
1160	Caretta caretta	2014	2014-07-11	2014-08-01	40	14.3	other causes	Malnutrition	Released
1161	Caretta caretta	2014	2014-07-16	2014-08-07	18.5	1.1	Infectious disease		Released
1162	Caretta caretta	2014	2014-07-16	2014-08-08	45	17	Entanglement		Released
1163	Caretta caretta	2014	2014-07-18	2014-08-08	18	1	other causes	Malnutrition	Released
1164	Caretta caretta	2014	2014-07-22	2014-08-11	17.5	1	Infectious disease		Died during hospitalization
1165	Caretta caretta	2014	2014-07-27	2014-09-11	41	11.5	Unknown/undetermined		Released
1166	Caretta caretta	2014	2014-08-02	2014-08-12	16.5	0.7	Unknown/undetermined		Died during hospitalization
1167	Caretta caretta	2014	2014-08-05				Unknown/undetermined		Admitted dead
1168	Caretta caretta	2014	2014-08-07	2014-08-22	34.5	7.4	other causes	Malnutrition	Released
1169	Caretta caretta	2014	2014-08-09	2014-08-24	35.8	7.3	other causes	Malnutrition	Released
1170	Caretta caretta	2014	2014-08-13	2014-08-31	37	10.3	Entanglement		Released
1171	Caretta caretta	2014	2014-08-15	2014-09-05	52	19	Entanglement		Released
1172	Caretta caretta	2014	2014-08-16	2014-09-11			Entanglement		Released
1173	Caretta caretta	2014	2014-08-19	2014-09-12	46	14.5	Unknown/undetermined		Released
1174	Caretta caretta	2014	2014-08-19	2014-09-11	31	4.6	Entanglement		Released
1175	Caretta caretta	2014	2014-08-22	2014-08-26	33.5	8.5	Entanglement		Released
1176	Caretta caretta	2014	2014-08-22	2014-09-12	47	14	Entanglement		Released
1177	Caretta caretta	2014	2014-08-29	2014-09-26	20.8	1.4	other causes	Malnutrition	Released
1178	Caretta caretta	2014	2014-08-29	2014-09-28	34.1	6.9	Unknown/undetermined		Released
1179	Caretta caretta	2014	2014-08-29	2014-09-12	28.9	4.7	Entanglement		Released
1180	Caretta caretta	2014	2014-09-02	2014-09-28	20.2	1.5	other causes	Malnutrition	Released
1181	Caretta caretta	2014	2014-09-02	2014-09-26	20.5	1.4	other causes	Malnutrition	Released
1182	Caretta caretta	2014	2014-09-03	2014-09-06	27.2	2.1	Infectious disease		Died during hospitalization
1183	Caretta caretta	2014	2014-09-14	2014-10-14	39.2	9.2	Entanglement		Released
1184	Caretta caretta	2014	2014-09-25	2014-10-14	34	5.8	Entanglement		Released
1185	Caretta caretta	2014	2014-10-01	2014-11-07	22.5	1.8	Entanglement		Released
1186	Caretta caretta	2014	2014-10-08	2014-11-22			Entanglement		Released
1187	Caretta caretta	2014	2014-10-23	2014-11-07	30	4.2	Entanglement		Released
1188	Caretta caretta	2015	2015-02-01	2015-04-18	36.2	13.2	Entanglement		Died during hospitalization
1189	Caretta caretta	2015	2015-02-03				Unknown/undetermined		Admitted dead
1190	Caretta caretta	2015	2015-03-06	2015-04-24	47	15	other causes	Malnutrition	Released
1191	Caretta caretta	2015	2015-03-26	2015-04-16	22.5	2.2	Unknown/undetermined		Released
1192	Caretta caretta	2015	2015-03-26	2015-04-16	23	2.1	Unknown/undetermined		Released
1193	Caretta caretta	2015	2015-03-29	2015-04-01	28.8	4	other causes	Malnutrition	Died during hospitalization
1194	Caretta caretta	2015	2015-04-05	2015-05-03	21	1.5	Entanglement		Released
1195	Caretta caretta	2015	2015-04-21	2015-06-17	23	2.2	Infectious disease		Released
1196	Caretta caretta	2015	2015-04-25	2015-05-16	28.7	4.3	Unknown/undetermined		Released
1197	Caretta caretta	2015	2015-04-25	2015-05-16	33	5.5	Crude oil		Released
1198	Caretta caretta	2015	2015-04-27		22.8	1.6	Unknown/undetermined		Admitted dead
1199	Caretta caretta	2015	2015-04-29	2015-05-02	29.9	4.1	other causes	Malnutrition	Died during hospitalization
1200	Caretta caretta	2015	2015-05-07	2015-06-18	30.8	4.7	Infectious disease		Released
1201	Caretta caretta	2015	2015-05-08				Unknown/undetermined		Admitted dead
1202	Caretta caretta	2015	2015-05-10	2015-06-18	27	3	Entanglement		Released
1203	Caretta caretta	2015	2015-05-19	2015-06-07	42.5	11.5	Hooks/monofilament lines		Released
1204	Caretta caretta	2015	2015-05-28	2015-06-08	45.5	14.5	Entanglement		Released
1205	Caretta caretta	2015	2015-05-28				Entanglement		Admitted dead
1206	Caretta caretta	2015	2015-05-29	2015-06-08	35	7.5	Unknown/undetermined		Released
1207	Caretta caretta	2015	2015-05-30	2015-09-27	35.5	6.1	Entanglement		Released
1208	Caretta caretta	2015	2015-06-01	2015-06-17	24.7	2.7	Entanglement		Released
1209	Caretta caretta	2015	2015-06-01	2015-07-21	58.5	31.5	Entanglement		Released
1210	Caretta caretta	2015	2015-06-12	2015-07-03	22.5	1.9	Entanglement		Released
1211	Caretta caretta	2015	2015-06-17	2015-07-16	42	12.5	other causes	Malnutrition	Released
1212	Caretta caretta	2015	2015-06-18	2015-08-13			other causes	Malnutrition	Released
1213	Caretta caretta	2015	2015-06-18	2015-08-04	31	4.1	Entanglement		Released
1214	Caretta caretta	2015	2015-06-20	2015-07-23	25.5	2.7	Entanglement		Released
1215	Caretta caretta	2015	2015-06-21	2015-07-23	27.5	3.5	Entanglement		Released
1216	Caretta caretta	2015	2015-06-23	2015-07-16	41	10.6	Infectious disease		Released
1217	Caretta caretta	2015	2015-06-24	2015-07-21	26	2.8	Infectious disease		Released
1218	Caretta caretta	2015	2015-06-25	2015-08-11	17.5	0.8	Infectious disease		Released

Record ID	Species	Year	Admission date	Departure date	SCL (cm)	Body weight (kg)	Cause of admission	Other-cause subcategory	Final disposition
1219	Caretta caretta	2015	2015-06-26	2015-07-21	31.2	5	other causes	Malnutrition	Released
1220	Caretta caretta	2015	2015-06-28	2015-07-03	29.5	3	Entanglement		Released
1221	Caretta caretta	2015	2015-06-29	2015-08-04	38.8	12.6	other causes	Malnutrition	Released
1222	Caretta caretta	2015	2015-07-01				Unknown/undetermined		Admitted dead
1223	Caretta caretta	2015	2015-07-02	2015-07-16	18	1.2	other causes	Malnutrition	Released
1224	Caretta caretta	2015	2015-07-04		60		Entanglement		Admitted dead
1225	Caretta caretta	2015	2015-07-09	2015-07-23	24.5	2.4	Infectious disease		Released
1226	Caretta caretta	2015	2015-07-14		20	1.2	Entanglement		Admitted dead
1227	Caretta caretta	2015	2015-07-16	2015-07-23	35	7.5	Infectious disease		Released
1228	Caretta caretta	2015	2015-07-16	2015-08-13	24	2.2	Entanglement		Released
1229	Caretta caretta	2015	2015-07-17	2015-07-20	49	20	Hooks/monofilament lines		Died during hospitalization
1230	Caretta caretta	2015	2015-07-20				Unknown/undetermined		Admitted dead
1231	Caretta caretta	2015	2015-07-21	2015-08-04	36.5	7.5	Infectious disease		Released
1232	Caretta caretta	2015	2015-07-21	2015-08-20	39	10	Entanglement		Released
1233	Caretta caretta	2015	2015-07-22	2015-09-24	19.9	1.7	Entanglement		Released
1234	Caretta caretta	2015	2015-07-22	2015-08-04	32.5	5.7	Entanglement		Released
1235	Caretta caretta	2015	2015-07-25				Unknown/undetermined		Admitted dead
1236	Caretta caretta	2015	2015-07-25				Unknown/undetermined		Admitted dead
1237	Caretta caretta	2015	2015-07-27	2015-08-11	18.5	1.1	other causes	Malnutrition	Released
1238	Caretta caretta	2015	2015-07-29	2015-08-09	55		Infectious disease		Died during hospitalization
1239	Caretta caretta	2015	2015-08-03	2015-09-24	21.5	1.7	Entanglement		Released
1240	Caretta caretta	2015	2015-08-04	2015-08-13	22.1	1.8	Entanglement		Released
1241	Caretta caretta	2015	2015-08-04	2015-08-20	24	2.1	Entanglement		Released
1242	Caretta caretta	2015	2015-08-05	2015-08-11	37		Entanglement		Released
1243	Caretta caretta	2015	2015-08-11	2016-01-23	34	5.2	Entanglement		Released
1244	Caretta caretta	2015	2015-08-16	2015-09-12	20.5	1.3	Entanglement		Released
1245	Caretta caretta	2015	2015-08-16	2015-08-20	31.5	3.8	Entanglement		Released
1246	Caretta caretta	2015	2015-08-24				Unknown/undetermined		Admitted dead
1247	Caretta caretta	2015	2015-08-25	2015-09-17	39	10	Entanglement		Released
1248	Caretta caretta	2015	2015-08-28	2015-08-30			Hooks/monofilament lines		Died during hospitalization
1249	Caretta caretta	2015	2015-08-28		79	90	Unknown/undetermined		Admitted dead
1250	Caretta caretta	2015	2015-09-01	2015-09-12	18.5	1.1	Infectious disease		Released
1251	Caretta caretta	2015	2015-09-04	2015-09-27	37.4	6	Entanglement		Released
1252	Caretta caretta	2015	2015-09-06	2015-09-11	34.2	6.7	other causes	Malnutrition	Died during hospitalization
1253	Caretta caretta	2015	2015-09-10	2015-10-22	19.1	1.1	Entanglement		Released
1254	Caretta caretta	2015	2015-09-14	2015-10-15	18.5	0.9	Entanglement		Released
1255	Caretta caretta	2015	2015-09-16	2016-01-23	30.6	5.5	Entanglement		Released
1256	Caretta caretta	2015	2015-09-20		45		Entanglement		Admitted dead
1257	Caretta caretta	2015	2015-09-22	2015-11-19	36.8	6	Entanglement		Died during hospitalization
1258	Caretta caretta	2015	2015-09-23	2015-11-23	37	7.2	Entanglement		Released
1259	Caretta caretta	2015	2015-09-30	2015-09-30	33.8	7.3	Entanglement		Released
1260	Caretta caretta	2015	2015-10-04	2015-10-22	25	2.4	Entanglement		Released
1261	Caretta caretta	2015	2015-10-08	2015-10-22	31.5	4.3	Entanglement		Released
1262	Caretta caretta	2015	2015-10-26	2015-11-10	53.5	28.5	Hooks/monofilament lines		Released
1263	Caretta caretta	2015	2015-11-02				Unknown/undetermined		Admitted dead
1264	Caretta caretta	2015	2015-11-06	2015-11-23	42	10.9	Entanglement		Released
1265	Caretta caretta	2015	2015-12-24				Unknown/undetermined		Admitted dead
1266	Caretta caretta	2016	2016-01-29	2016-05-24	42.2	12.9	Entanglement		Released
1267	Caretta caretta	2016	2016-01-29	2016-04-07	44.5	11.6	Entanglement		Released
1268	Caretta caretta	2016	2016-02-01	2016-02-08			Infectious disease		Died during hospitalization
1269	Caretta caretta	2016	2016-02-09	2016-02-15	45.8	12.5	Unknown/undetermined		Released
1270	Caretta caretta	2016	2016-03-12	2016-05-28	28	4.3	Entanglement		Released
1271	Caretta caretta	2016	2016-03-17	2016-05-17	36.5	12.8	Entanglement		Released
1272	Caretta caretta	2016	2016-03-19	2016-04-07	30.8	5	Entanglement		Released
1273	Caretta caretta	2016	2016-04-03	2016-04-04	36	5.7	other causes	Malnutrition	Released
1274	Caretta caretta	2016	2016-04-04	2016-04-15	23	2	Entanglement		Released
1275	Caretta caretta	2016	2016-04-06	2016-05-24	38.8	9.5	Entanglement		Released
1276	Caretta caretta	2016	2016-04-29	2016-05-28			Entanglement		Released
1277	Caretta caretta	2016	2016-05-01	2016-06-17	32	5.6	other causes	Malnutrition	Released
1278	Caretta caretta	2016	2016-05-04	2016-06-01	37	7.9	Infectious disease		Released
1279	Caretta caretta	2016	2016-05-12		30		Unknown/undetermined		Admitted dead
1280	Caretta caretta	2016	2016-05-16	2016-07-06	18.8	1.1	Unknown/undetermined		Released
1281	Caretta caretta	2016	2016-05-17	2016-05-19	18.5	0.3	Unknown/undetermined		Released
1282	Caretta caretta	2016	2016-05-17				Unknown/undetermined		Admitted dead
1283	Caretta caretta	2016	2016-05-17	2016-07-06	33.5	6.3	Entanglement		Released
1284	Caretta caretta	2016	2016-05-23	2016-06-07	22	1.7	Infectious disease		Released
1285	Caretta caretta	2016	2016-05-23	2016-07-28	22	1.7	Entanglement		Released
1286	Caretta caretta	2016	2016-05-24	2016-06-17	40.5	8	Unknown/undetermined		Released
1287	Caretta caretta	2016	2016-05-24	2016-07-13	57.6	17.3	Unknown/undetermined		Released
1288	Caretta caretta	2016	2016-05-26	2016-07-14	39	11.8	Infectious disease		Released
1289	Caretta caretta	2016	2016-05-31	2016-06-07	16	0.7	Unknown/undetermined		Released
1290	Caretta caretta	2016	2016-06-01	2016-07-13	42	10.7	Hooks/monofilament lines		Released

Record ID	Species	Year	Admission date	Departure date	SCL (cm)	Body weight (kg)	Cause of admission	Other-cause subcategory	Final disposition
1291	Caretta caretta	2016	2016-06-03	2016-08-02	23	1.7	Infectious disease		Released
1292	Caretta caretta	2016	2016-06-05	2016-08-03	42	13.4	Hooks/monofilament lines		Released
1293	Caretta caretta	2016	2016-06-06	2016-06-07	15.5	0.7	Unknown/undetermined		Released
1294	Caretta caretta	2016	2016-06-06	2016-06-06	16.8	0.8	Unknown/undetermined		Released
1295	Caretta caretta	2016	2016-06-06	2016-06-07	19.9	1.6	Unknown/undetermined		Released
1296	Caretta caretta	2016	2016-06-07	2016-09-04	25.5	3	Entanglement		Released
1297	Caretta caretta	2016	2016-06-08	2016-07-28	19.5	1.3	Infectious disease		Released
1298	Caretta caretta	2016	2016-06-10	2016-07-06	53.5	23	Unknown/undetermined		Released
1299	Caretta caretta	2016	2016-06-12	2016-08-19	18.1	1.3	other causes	Malnutrition	Died during hospitalization
1300	Caretta caretta	2016	2016-06-12	2016-06-15	18	0.9	Unknown/undetermined		Released
1301	Caretta caretta	2016	2016-06-12	2016-07-28	36.2	7	Infectious disease		Released
1302	Caretta caretta	2016	2016-06-12	2016-07-28	25	2.4	Entanglement		Released
1303	Caretta caretta	2016	2016-06-12	2016-07-28	29	3.4	Entanglement		Released
1304	Caretta caretta	2016	2016-06-13	2016-08-02	38.5	13	other causes	Shark attack	Released
1305	Caretta caretta	2016	2016-06-13	2016-10-15	28.5	3.2	Entanglement		Died during hospitalization
1306	Caretta caretta	2016	2016-06-14	2016-06-17			other causes	Miscellany	Released
1307	Caretta caretta	2016	2016-06-14	2016-06-22	15.5	0.6	other causes	Miscellany	Released
1308	Caretta caretta	2016	2016-06-14	2016-06-17	17	0.9	other causes	Miscellany	Released
1309	Caretta caretta	2016	2016-06-14		35		Unknown/undetermined		Admitted dead
1310	Caretta caretta	2016	2016-06-15	2016-06-20			other causes	Miscellany	Released
1311	Caretta caretta	2016	2016-06-15	2016-06-20	16.5	0.8	other causes	Miscellany	Released
1312	Caretta caretta	2016	2016-06-15	2016-06-22	17	0.7	other causes	Miscellany	Released
1313	Caretta caretta	2016	2016-06-15	2016-06-20	19	1	other causes	Miscellany	Released
1314	Caretta caretta	2016	2016-06-18	2016-07-06	36.5	6.2	Entanglement		Released
1315	Caretta caretta	2016	2016-06-19	2016-10-15	44	3.2	trauma		Released
1316	Caretta caretta	2016	2016-06-20	2016-07-01	15	0.5	Unknown/undetermined		Released
1317	Caretta caretta	2016	2016-06-20	2016-06-22	16	0.6	Unknown/undetermined		Released
1318	Caretta caretta	2016	2016-06-20				Unknown/undetermined		Admitted dead
1319	Caretta caretta	2016	2016-06-28	2016-07-06	21.5	2.1	other causes	Miscellany	Released
1320	Caretta caretta	2016	2016-07-01	2016-07-06	20.9	1.4	other causes	Miscellany	Released
1321	Caretta caretta	2016	2016-07-02				trauma		Admitted dead
1322	Caretta caretta	2016	2016-07-05	2016-08-03	18	1	other causes	Malnutrition	Released
1323	Caretta caretta	2016	2016-07-08	2016-08-16	14.5	0.7	Infectious disease		Died during hospitalization
1324	Caretta caretta	2016	2016-07-09		40	6	Unknown/undetermined		Admitted dead
1325	Caretta caretta	2016	2016-07-10		44	6	Unknown/undetermined		Admitted dead
1326	Caretta caretta	2016	2016-07-11	2016-08-24	27	2.6	Entanglement		Released
1327	Caretta caretta	2016	2016-07-12	2016-08-24	35	5.8	other causes	Buoyancy disorder	Released
1328	Caretta caretta	2016	2016-07-12	2016-08-03	35.5	7.7	other causes	Buoyancy disorder	Released
1329	Caretta caretta	2016	2016-07-14	2016-07-28	18.5	1.2	other causes	Buoyancy disorder	Released
1330	Caretta caretta	2016	2016-07-15	2016-07-28	21	1.5	other causes	Miscellany	Released
1331	Caretta caretta	2016	2016-07-15	2016-08-03	35.5	6.8	other causes	Miscellany	Released
1332	Caretta caretta	2016	2016-07-16	2016-07-28	17	0.8	other causes	Buoyancy disorder	Released
1333	Caretta caretta	2016	2016-07-16		22	1.7	Unknown/undetermined		Admitted dead
1334	Caretta caretta	2016	2016-07-20		52	6.2	Unknown/undetermined		Admitted dead
1335	Caretta caretta	2016	2016-07-20	2016-09-17	30	3.6	Entanglement		Released
1336	Caretta caretta	2016	2016-07-21	2016-07-28	18.5	1.1	other causes	Malnutrition	Released
1337	Caretta caretta	2016	2016-07-22	2016-07-26	21.5	1.7	other causes	Miscellany	Released
1338	Caretta caretta	2016	2016-07-24	2016-08-23	18	0.7	Entanglement		Died during hospitalization
1339	Caretta caretta	2016	2016-07-25	2016-08-03	16.2	0.8	other causes	Miscellany	Released
1340	Caretta caretta	2016	2016-07-27	2016-08-03			Entanglement		Released
1341	Caretta caretta	2016	2016-07-31	2016-08-24	17.5	0.9	other causes	Malnutrition	Released
1342	Caretta caretta	2016	2016-08-18				Entanglement		Admitted dead
1343	Caretta caretta	2016	2016-08-22	2016-11-18	38	8.3	Entanglement		Released
1344	Caretta caretta	2016	2016-08-23	2016-10-14	39.2	9.1	Entanglement		Released
1345	Caretta caretta	2016	2016-09-05	2016-09-22	45.5	15.5	Hooks/monofilament lines		Released
1346	Caretta caretta	2016	2016-09-06	2016-10-14	36	6.6	Entanglement		Released
1347	Caretta caretta	2016	2016-09-06	2016-10-14	39	7.9	Entanglement		Released
1348	Caretta caretta	2016	2016-09-07	2016-09-17	35.5	6.8	Infectious disease		Released
1349	Caretta caretta	2016	2016-09-07	2016-10-06	42.5	9.7	Entanglement		Released
1350	Caretta caretta	2016	2016-09-08	2016-09-22	47.5	17	Unknown/undetermined		Released
1351	Caretta caretta	2016	2016-09-09		44	14.5	Unknown/undetermined		Admitted dead
1352	Caretta caretta	2016	2016-09-13	2016-10-06	31	4.2	Entanglement		Released
1353	Caretta caretta	2016	2016-09-22	2016-11-09	27	2.6	other causes	Malnutrition	Released
1354	Caretta caretta	2016	2016-09-25	2016-11-15	53	24.5	Entanglement		Released
1355	Caretta caretta	2016	2016-10-05	2016-11-10	52	29.5	Unknown/undetermined		Released
1356	Caretta caretta	2016	2016-10-17	2016-10-25	21	1.4	other causes	Malnutrition	Died during hospitalization
1357	Caretta caretta	2016	2016-10-20	2016-11-15	32	4.9	other causes	Malnutrition	Released
1358	Caretta caretta	2016	2016-11-14	2016-12-07	21	1.3	Infectious disease		Died during hospitalization
1359	Caretta caretta	2016	2016-12-08	2017-12-08			Hooks/monofilament lines		Released
1360	Caretta caretta	2017	2017-01-05	2017-01-18	59	39	Hooks/monofilament lines		Released
1361	Caretta caretta	2017	2017-01-20				Unknown/undetermined		Admitted dead
1362	Caretta caretta	2017	2017-01-20				Unknown/undetermined		Admitted dead

Record ID	Species	Year	Admission date	Departure date	SCL (cm)	Body weight (kg)	Cause of admission	Other-cause subcategory	Final disposition
1363	Caretta caretta	2017	2017-04-06	2017-04-26	26.3	3	other causes	Malnutrition	Released
1364	Caretta caretta	2017	2017-04-13	2017-05-17	34.6	6	Entanglement		Released
1365	Caretta caretta	2017	2017-04-27	2017-06-28	35	6	trauma		Died during hospitalization
1366	Caretta caretta	2017	2017-05-02				other causes	Buoyancy disorder	Admitted dead
1367	Caretta caretta	2017	2017-05-19	2017-06-04	29.5	4	Entanglement		Released
1368	Caretta caretta	2017	2017-05-23	2017-05-23	47	17	Unknown/undetermined		Released
1369	Caretta caretta	2017	2017-05-28	2017-08-13	51	22	Infectious disease		Released
1370	Caretta caretta	2017	2017-06-05	2017-06-10	44	25	Infectious disease		Released
1371	Caretta caretta	2017	2017-06-05	2017-06-10	25	2	Entanglement		Released
1372	Caretta caretta	2017	2017-06-06	2017-08-07	43.5	18.8	Infectious disease		Released
1373	Caretta caretta	2017	2017-06-06	2017-06-22	29	3.4	Entanglement		Released
1374	Caretta caretta	2017	2017-06-11				Unknown/undetermined		Admitted dead
1375	Caretta caretta	2017	2017-06-14	2017-08-07	41	10.2	Entanglement		Released
1376	Caretta caretta	2017	2017-06-17	2017-09-20	38	8.7	Entanglement		Euthanized
1377	Caretta caretta	2017	2017-06-19	2017-09-02	44	9	Entanglement		Released
1378	Caretta caretta	2017	2017-06-21	2017-08-07	29	3	Infectious disease		Released
1379	Caretta caretta	2017	2017-06-26	2017-09-02	33.2	5.7	Entanglement		Released
1380	Caretta caretta	2017	2017-06-29	2017-08-07	30	5.2	other causes	Malnutrition	Released
1381	Caretta caretta	2017	2017-06-29	2017-09-02	27.8	3.6	Entanglement		Released
1382	Caretta caretta	2017	2017-07-02	2017-08-07	37.5	8.5	Infectious disease		Released
1383	Caretta caretta	2017	2017-07-07	2017-09-30	36	7.8	Entanglement		Released
1384	Caretta caretta	2017	2017-07-12	2017-09-30	38	9.1	Entanglement		Released
1385	Caretta caretta	2017	2017-07-20	2017-08-02	40.5	11.4	other causes	Malnutrition	Died during hospitalization
1386	Caretta caretta	2017	2017-07-27	2017-08-27	38.5	9	Entanglement		Released
1387	Caretta caretta	2017	2017-08-01	2017-12-05	30	3.9	Entanglement		Released
1388	Caretta caretta	2017	2017-08-02	2017-09-02	19	1.1	other causes	Malnutrition	Released
1389	Caretta caretta	2017	2017-08-07	2017-10-20	29.5	4.3	Entanglement		Released
1390	Caretta caretta	2017	2017-08-08	2017-08-27	52	11	Infectious disease		Released
1391	Caretta caretta	2017	2017-08-11	2017-08-12			Infectious disease		Died during hospitalization
1392	Caretta caretta	2017	2017-08-12		54		Hooks/monofilament lines		Admitted dead
1393	Caretta caretta	2017	2017-08-13	2017-09-02	52	19.6	Entanglement		Released
1394	Caretta caretta	2017	2017-08-14		62		Hooks/monofilament lines		Admitted dead
1395	Caretta caretta	2017	2017-08-17	2017-08-17	38	7.7	Entanglement		Released
1396	Caretta caretta	2017	2017-08-18				Unknown/undetermined		Admitted dead
1397	Caretta caretta	2017	2017-08-25				Unknown/undetermined		Admitted dead
1398	Caretta caretta	2017	2017-08-27				trauma		Admitted dead
1399	Caretta caretta	2017	2017-09-08				Unknown/undetermined		Admitted dead
1400	Caretta caretta	2017	2017-09-14	2017-12-14	26	2.5	other causes	Malnutrition	Released
1401	Caretta caretta	2017	2017-09-20	2017-10-20	45	13.6	Hooks/monofilament lines		Released
1402	Caretta caretta	2017	2017-10-02	2017-10-02	44	14.9	other causes	Malnutrition	Released
1403	Caretta caretta	2017	2017-11-01	2017-12-14	41	10.2	Entanglement		Released
1404	Caretta caretta	2017	2017-11-12	2017-12-05	31	4.8	Entanglement		Released
1405	Caretta caretta	2017	2017-11-17				Unknown/undetermined		Admitted dead
1406	Caretta caretta	2017	2017-11-23	2017-11-25	70	60	Hooks/monofilament lines		Died during hospitalization
1407	Caretta caretta	2017	2017-12-17	2018-05-23	34	6.8	Entanglement		Released
1408	Caretta caretta	2017	2017-12-31	2018-03-21			Hooks/monofilament lines		Released
1409	Caretta caretta	2018	2018-01-02		39	7.7	Unknown/undetermined		Admitted dead
1410	Caretta caretta	2018	2018-01-06		63	50	Hooks/monofilament lines		Admitted dead
1411	Caretta caretta	2018	2018-01-11	2018-02-21	51	20	Infectious disease		Released
1412	Caretta caretta	2018	2018-02-01		55	24	Hooks/monofilament lines		Admitted dead
1413	Caretta caretta	2018	2018-02-14	2018-02-21	53	22	Infectious disease		Released
1414	Caretta caretta	2018	2018-02-15	2018-05-23	38	9	other causes	Miscellany	Released
1415	Caretta caretta	2018	2018-02-17	2018-04-04	48	16	other causes	Miscellany	Released
1416	Caretta caretta	2018	2018-03-06				Entanglement		Admitted dead
1417	Caretta caretta	2018	2018-03-29	2018-05-19	36	7	other causes	Buoyancy disorder	Released
1418	Caretta caretta	2018	2018-04-06	2018-05-15	46		other causes	Malnutrition	Released
1419	Caretta caretta	2018	2018-04-09		40		Unknown/undetermined		Admitted dead
1420	Caretta caretta	2018	2018-04-12		19	1	Unknown/undetermined		Admitted dead
1421	Caretta caretta	2018	2018-04-16	2018-07-25	50		Entanglement		Released
1422	Caretta caretta	2018	2018-04-23	2018-06-05	29	4.6	Entanglement		Released
1423	Caretta caretta	2018	2018-04-26	2018-05-25	45		Unknown/undetermined		Released
1424	Caretta caretta	2018	2018-04-27	2018-06-03	29	4	Unknown/undetermined		Released
1425	Caretta caretta	2018	2018-04-29	2018-06-01	54		Unknown/undetermined		Released
1426	Caretta caretta	2018	2018-05-06		28		Unknown/undetermined		Admitted dead
1427	Caretta caretta	2018	2018-05-13	2018-07-26	34	7	Infectious disease		Released
1428	Caretta caretta	2018	2018-05-15	2018-06-06	43		Infectious disease		Released
1429	Caretta caretta	2018	2018-05-17				Unknown/undetermined		Admitted dead
1430	Caretta caretta	2018	2018-05-24		44		Unknown/undetermined		Admitted dead
1431	Caretta caretta	2018	2018-05-30		52		trauma		Admitted dead
1432	Caretta caretta	2018	2018-05-31				Unknown/undetermined		Admitted dead
1433	Caretta caretta	2018	2018-06-01	2018-07-02	40.8	10.7	Entanglement		Released
1434	Caretta caretta	2018	2018-06-03	2018-06-24	38	9.8	Infectious disease		Released

Record ID	Species	Year	Admission date	Departure date	SCL (cm)	Body weight (kg)	Cause of admission	Other-cause subcategory	Final disposition
1435	Caretta caretta	2018	2018-06-03	2018-08-11	25	2.5	Entanglement		Released
1436	Caretta caretta	2018	2018-06-06	2018-06-30	57	25.6	Entanglement		Died during hospitalization
1437	Caretta caretta	2018	2018-06-06		42	18	Entanglement		Admitted dead
1438	Caretta caretta	2018	2018-06-11	2018-06-17	43.5	11.4	Entanglement		Released
1439	Caretta caretta	2018	2018-06-11				Entanglement		Admitted dead
1440	Caretta caretta	2018	2018-06-12	2018-07-28	20	1	Infectious disease		Released
1441	Caretta caretta	2018	2018-06-13	2018-07-28	16.2	0.9	Infectious disease		Released
1442	Caretta caretta	2018	2018-06-14		30	4.8	Unknown/undetermined		Admitted dead
1443	Caretta caretta	2018	2018-06-15		36	7	Unknown/undetermined		Admitted dead
1444	Caretta caretta	2018	2018-06-16	2018-06-16	28	2	Infectious disease		Died during hospitalization
1445	Caretta caretta	2018	2018-06-20	2018-07-25			Entanglement		Released
1446	Caretta caretta	2018	2018-06-20	2018-07-23	35	7	Entanglement		Released
1447	Caretta caretta	2018	2018-06-26	2018-07-26	45	15	Unknown/undetermined		Released
1448	Caretta caretta	2018	2018-06-27	2018-07-20	37	8	Entanglement		Released
1449	Caretta caretta	2018	2018-07-03		40		Unknown/undetermined		Admitted dead
1450	Caretta caretta	2018	2018-07-06			3	Unknown/undetermined		Admitted dead
1451	Caretta caretta	2018	2018-07-09	2018-08-11	17	1	Unknown/undetermined		Released
1452	Caretta caretta	2018	2018-07-16	2018-08-20	19	1	other causes	Malnutrition	Released
1453	Caretta caretta	2018	2018-07-24	2018-10-19	46	16	Infectious disease		Released
1454	Caretta caretta	2018	2018-07-26	2018-09-10	44	14	other causes	Buoyancy disorder	Released
1455	Caretta caretta	2018	2018-07-31	2018-09-15	52	23	other causes	Buoyancy disorder	Released
1456	Caretta caretta	2018	2018-08-03	2018-10-19	46	16	other causes	Malnutrition	Released
1457	Caretta caretta	2018	2018-08-03	2018-10-19	41	12	Unknown/undetermined		Released
1458	Caretta caretta	2018	2018-08-05	2018-10-19	44	13	Infectious disease		Released
1459	Caretta caretta	2018	2018-08-10		42	12	Entanglement		Admitted dead
1460	Caretta caretta	2018	2018-08-12	2018-10-19	49	18	Unknown/undetermined		Released
1461	Caretta caretta	2018	2018-08-15				Entanglement		Admitted dead
1462	Caretta caretta	2018	2018-08-17	2018-09-12	33	5	other causes	Malnutrition	Died during hospitalization
1463	Caretta caretta	2018	2018-08-21	2018-10-19	29		other causes	Malnutrition	Released
1464	Caretta caretta	2018	2018-08-21	2018-10-19	51	21	other causes	Malnutrition	Released
1465	Caretta caretta	2018	2018-08-25		32		Unknown/undetermined		Admitted dead
1466	Caretta caretta	2018	2018-08-25		35		Unknown/undetermined		Admitted dead
1467	Caretta caretta	2018	2018-08-26		71		Unknown/undetermined		Admitted dead
1468	Caretta caretta	2018	2018-08-26	2018-10-19	26	3	Entanglement		Released
1469	Caretta caretta	2018	2018-09-01	2018-09-26	19	1	other causes	Shark attack	Released
1470	Caretta caretta	2018	2018-09-01	2018-10-09	20	1	Unknown/undetermined		Died during hospitalization
1471	Caretta caretta	2018	2018-09-05	2018-10-19	27	3	other causes	Buoyancy disorder	Released
1472	Caretta caretta	2018	2018-09-06	2018-10-12	26	3	other causes	Buoyancy disorder	Released
1473	Caretta caretta	2018	2018-09-07				Entanglement		Admitted dead
1474	Caretta caretta	2018	2018-09-08	2018-09-15	24	2	Unknown/undetermined		Released
1475	Caretta caretta	2018	2018-09-09	2018-12-05	32	5	Entanglement		Released
1476	Caretta caretta	2018	2018-09-10				Entanglement		Admitted dead
1477	Caretta caretta	2018	2018-09-12				Unknown/undetermined		Admitted dead
1478	Caretta caretta	2018	2018-09-12				Unknown/undetermined		Admitted dead
1479	Caretta caretta	2018	2018-09-13	2018-10-19	49.9	14.3	Entanglement		Released
1480	Caretta caretta	2018	2018-09-14	2018-10-06			other causes	Malnutrition	Released
1481	Caretta caretta	2018	2018-09-14	2018-10-19	30.6	4.6	Infectious disease		Released
1482	Caretta caretta	2018	2018-09-16	2018-10-19	28	3	Entanglement		Released
1483	Caretta caretta	2018	2018-09-25	2018-10-19	31.8	4.9	Entanglement		Released
1484	Caretta caretta	2018	2018-09-26				trauma		Admitted dead
1485	Caretta caretta	2018	2018-09-29	2019-10-19	25	2	Unknown/undetermined		Released
1486	Caretta caretta	2018	2018-10-01	2018-10-19	37.5	9.2	other causes	Malnutrition	Released
1487	Caretta caretta	2018	2018-10-10	2018-10-19	60		Hooks/monofilament lines		Released
1488	Caretta caretta	2018	2018-10-11	2018-10-19	41	10	Unknown/undetermined		Released
1489	Caretta caretta	2018	2018-10-14		70		Unknown/undetermined		Admitted dead
1490	Caretta caretta	2018	2018-10-16	2018-10-19	41	11	Entanglement		Released
1491	Caretta caretta	2018	2018-10-24	2018-12-05	23	2	Entanglement		Released
1492	Caretta caretta	2018	2018-11-02	2018-11-12	28	4.4	trauma		Euthanized
1493	Caretta caretta	2018	2018-11-09	2018-12-05	56	24.2	Hooks/monofilament lines		Released
1494	Caretta caretta	2018	2018-12-07	2019-03-15	46		trauma		Died during hospitalization
1495	Caretta caretta	2018	2018-12-09	2019-02-07	36.1	8	Entanglement		Released
1496	Caretta caretta	2018	2018-12-17	2019-02-07	35	6	Entanglement		Released
1497	Caretta caretta	2018	2018-12-28	2019-02-07	40	9	Entanglement		Released
1498	Caretta caretta	2019	2019-02-22	2019-02-22			Unknown/undetermined		Released
1499	Caretta caretta	2019	2019-02-22				Unknown/undetermined		Admitted dead
1500	Caretta caretta	2019	2019-02-25	2019-03-13	37	8	other causes	Malnutrition	Released
1501	Caretta caretta	2019	2019-02-26	2019-03-13	44	12	other causes	Miscellany	Released
1502	Caretta caretta	2019	2019-02-27		38		Unknown/undetermined		Admitted dead
1503	Caretta caretta	2019	2019-03-03	2019-03-29	54.5	23.4	Infectious disease		Released
1504	Caretta caretta	2019	2019-03-10	2019-03-21	56.2	28.5	Unknown/undetermined		Released
1505	Caretta caretta	2019	2019-03-17		41.5		Unknown/undetermined		Admitted dead
1506	Caretta caretta	2019	2019-03-23	2019-07-24	23	1.4	other causes	Malnutrition	Released

Record ID	Species	Year	Admission date	Departure date	SCL (cm)	Body weight (kg)	Cause of admission	Other-cause subcategory	Final disposition
1507	Caretta caretta	2019	2019-03-29		42		trauma		Admitted dead
1508	Caretta caretta	2019	2019-04-06		63.4		Unknown/undetermined		Admitted dead
1509	Caretta caretta	2019	2019-04-07	2020-01-28	45	13	trauma		Released
1510	Caretta caretta	2019	2019-04-07	2019-04-11	54	27.4	other causes	Malnutrition	Released
1511	Caretta caretta	2019	2019-04-09	2019-06-16	36	7.5	Infectious disease		Died during hospitalization
1512	Caretta caretta	2019	2019-04-11				Entanglement		Admitted dead
1513	Caretta caretta	2019	2019-04-13	2019-05-18	24.1	2.2	Entanglement		Released
1514	Caretta caretta	2019	2019-04-26	2019-06-08	29	3	Entanglement		Released
1515	Caretta caretta	2019	2019-04-26	2019-06-23	30	4	Entanglement		Died during hospitalization
1516	Caretta caretta	2019	2019-04-28	2019-05-03	19	1	Unknown/undetermined		Died during hospitalization
1517	Caretta caretta	2019	2019-04-30	2019-06-08	37	7	other causes	Malnutrition	Released
1518	Caretta caretta	2019	2019-04-30	2019-05-18	39	10	Unknown/undetermined		Released
1519	Caretta caretta	2019	2019-05-02		36	9	Unknown/undetermined		Admitted dead
1520	Caretta caretta	2019	2019-05-03		32	2	Unknown/undetermined		Admitted dead
1521	Caretta caretta	2019	2019-05-07		35	7.5	trauma		Admitted dead
1522	Caretta caretta	2019	2019-05-11		36		Unknown/undetermined		Admitted dead
1523	Caretta caretta	2019	2019-05-11	2019-07-25	27	3.3	Entanglement		Died during hospitalization
1524	Caretta caretta	2019	2019-05-13	2019-06-08	40.8	11.9	Entanglement		Released
1525	Caretta caretta	2019	2019-05-14	2019-06-08	46		Entanglement		Released
1526	Caretta caretta	2019	2019-05-17	2019-06-07			other causes	Malnutrition	Died during hospitalization
1527	Caretta caretta	2019	2019-05-17	2019-08-02			Infectious disease		Released
1528	Caretta caretta	2019	2019-05-17				Entanglement		Admitted dead
1529	Caretta caretta	2019	2019-05-17				Entanglement		Admitted dead
1530	Caretta caretta	2019	2019-05-18	2019-06-08	25	2	Entanglement		Released
1531	Caretta caretta	2019	2019-05-18	2019-08-12	33	5	Entanglement		Released
1532	Caretta caretta	2019	2019-05-18	2019-08-12	39	9	Entanglement		Released
1533	Caretta caretta	2019	2019-05-19		27	3.4	Entanglement		Admitted dead
1534	Caretta caretta	2019	2019-05-24		42		Unknown/undetermined		Admitted dead
1535	Caretta caretta	2019	2019-05-24	2019-10-05	21	1.6	Entanglement		Released
1536	Caretta caretta	2019	2019-05-25	2019-06-29	20.5	1.4	other causes	Malnutrition	Died during hospitalization
1537	Caretta caretta	2019	2019-05-25		31	5	Unknown/undetermined		Admitted dead
1538	Caretta caretta	2019	2019-05-26		24	3	Unknown/undetermined		Admitted dead
1539	Caretta caretta	2019	2019-05-26		34	5	Unknown/undetermined		Admitted dead
1540	Caretta caretta	2019	2019-05-27	2019-07-10	19		other causes	Malnutrition	Released
1541	Caretta caretta	2019	2019-06-06		23	3	Unknown/undetermined		Admitted dead
1542	Caretta caretta	2019	2019-06-07	2019-07-19	26	3	Infectious disease		Released
1543	Caretta caretta	2019	2019-06-09	2019-06-26	23	2	other causes	Malnutrition	Died during hospitalization
1544	Caretta caretta	2019	2019-06-09		23	2	Unknown/undetermined		Admitted dead
1545	Caretta caretta	2019	2019-06-15	2019-07-06	35.5	8.4	Infectious disease		Released
1546	Caretta caretta	2019	2019-06-16	2019-06-22	17.5	1.1	other causes	Miscellany	Released
1547	Caretta caretta	2019	2019-06-16	2019-06-22	17.5	1	other causes	Miscellany	Released
1548	Caretta caretta	2019	2019-06-16	2019-06-26	39	9.6	other causes	Miscellany	Released
1549	Caretta caretta	2019	2019-06-16				Entanglement		Admitted dead
1550	Caretta caretta	2019	2019-06-17		28	3	Unknown/undetermined		Admitted dead
1551	Caretta caretta	2019	2019-06-18	2019-07-06	47	16.7	Infectious disease		Released
1552	Caretta caretta	2019	2019-06-21		53		Unknown/undetermined		Admitted dead
1553	Caretta caretta	2019	2019-06-22				Unknown/undetermined		Admitted dead
1554	Caretta caretta	2019	2019-06-23	2019-08-02	19.5	1.3	other causes	Malnutrition	Released
1555	Caretta caretta	2019	2019-06-23	2019-07-06	27	3.5	Infectious disease		Released
1556	Caretta caretta	2019	2019-06-25	2019-08-12	24	1.9	other causes	Malnutrition	Died during hospitalization
1557	Caretta caretta	2019	2019-06-29		24	2	Unknown/undetermined		Admitted dead
1558	Caretta caretta	2019	2019-06-30				Unknown/undetermined		Admitted dead
1559	Caretta caretta	2019	2019-06-30	2019-07-26	34	5.7	Entanglement		Released
1560	Caretta caretta	2019	2019-06-30	2019-08-04	34.5	6	Entanglement		Released
1561	Caretta caretta	2019	2019-07-01				Unknown/undetermined		Admitted dead
1562	Caretta caretta	2019	2019-07-01	2019-08-04	28	3.7	Entanglement		Released
1563	Caretta caretta	2019	2019-07-03	2019-08-07	19	1	Entanglement		Released
1564	Caretta caretta	2019	2019-07-03	2019-07-24	21.5	1.5	Entanglement		Released
1565	Caretta caretta	2019	2019-07-04	2019-07-26			other causes	Malnutrition	Released
1566	Caretta caretta	2019	2019-07-04	2019-07-10	20.5	1.6	other causes	Miscellany	Released
1567	Caretta caretta	2019	2019-07-04	2019-07-11	25	2.7	Entanglement		Released
1568	Caretta caretta	2019	2019-07-05	2019-08-12	24.9	2.5	Entanglement		Released
1569	Caretta caretta	2019	2019-07-06	2019-08-21	21.2	1.6	trauma		Released
1570	Caretta caretta	2019	2019-07-06	2019-08-13	23	1.8	Entanglement		Released
1571	Caretta caretta	2019	2019-07-12	2019-07-28	38.8	10.2	Infectious disease		Released
1572	Caretta caretta	2019	2019-07-12	2019-08-07	44	10.5	Entanglement		Died during hospitalization
1573	Caretta caretta	2019	2019-07-12				Entanglement		Admitted dead
1574	Caretta caretta	2019	2019-07-13				Unknown/undetermined		Admitted dead
1575	Caretta caretta	2019	2019-07-14	2019-07-24	22	1.5	other causes	Miscellany	Released
1576	Caretta caretta	2019	2019-07-15	2019-08-14	31	5	Entanglement		Released
1577	Caretta caretta	2019	2019-07-16				Unknown/undetermined		Admitted dead
1578	Caretta caretta	2019	2019-07-19	2019-08-02	18	1	Unknown/undetermined		Released

Record ID	Species	Year	Admission date	Departure date	SCL (cm)	Body weight (kg)	Cause of admission	Other-cause subcategory	Final disposition
1579	Caretta caretta	2019	2019-07-19				Unknown/undetermined		Admitted dead
1580	Caretta caretta	2019	2019-07-23	2019-07-26			Unknown/undetermined		Released
1581	Caretta caretta	2019	2019-07-26	2019-08-13	22	1	Unknown/undetermined		Released
1582	Caretta caretta	2019	2019-07-26	2019-08-07	23	2	Entanglement		Released
1583	Caretta caretta	2019	2019-07-27	2019-08-14			Entanglement		Released
1584	Caretta caretta	2019	2019-07-28	2019-08-14			other causes	Buoyancy disorder	Released
1585	Caretta caretta	2019	2019-07-28	2019-08-12			Entanglement		Released
1586	Caretta caretta	2019	2019-07-28	2019-08-14			Entanglement		Released
1587	Caretta caretta	2019	2019-07-30	2019-08-07			Unknown/undetermined		Released
1588	Caretta caretta	2019	2019-07-30	2019-08-13			Unknown/undetermined		Released
1589	Caretta caretta	2019	2019-07-30	2019-08-21			Unknown/undetermined		Released
1590	Caretta caretta	2019	2019-07-30	2019-08-14			Entanglement		Released
1591	Caretta caretta	2019	2019-08-01	2019-08-14	18	1	other causes	Malnutrition	Released
1592	Caretta caretta	2019	2019-08-02	2019-08-14	18	1	Unknown/undetermined		Released
1593	Caretta caretta	2019	2019-08-02				Unknown/undetermined		Admitted dead
1594	Caretta caretta	2019	2019-08-03	2019-09-28	35.9	7.2	other causes	Malnutrition	Released
1595	Caretta caretta	2019	2019-08-04	2019-09-10	19.9	1.4	Entanglement		Died during hospitalization
1596	Caretta caretta	2019	2019-08-06		21.5	0.6	Unknown/undetermined		Admitted dead
1597	Caretta caretta	2019	2019-08-07	2019-09-12	37.4	8.6	Entanglement		Released
1598	Caretta caretta	2019	2019-08-07	2019-09-06	32	5.2	Entanglement		Died during hospitalization
1599	Caretta caretta	2019	2019-08-07		36.2		Entanglement		Admitted dead
1600	Caretta caretta	2019	2019-08-08	2019-08-28	45	13	Hooks/monofilament lines		Released
1601	Caretta caretta	2019	2019-08-09	2019-08-21	16	0.9	other causes	Malnutrition	Released
1602	Caretta caretta	2019	2019-08-10	2019-08-30	41	6	Unknown/undetermined		Released
1603	Caretta caretta	2019	2019-08-10				Unknown/undetermined		Admitted dead
1604	Caretta caretta	2019	2019-08-10	2019-08-21	20	1	Entanglement		Released
1605	Caretta caretta	2019	2019-08-11	2019-09-21	34	6	Entanglement		Released
1606	Caretta caretta	2019	2019-08-12	2019-08-21	18	1	other causes	Miscellany	Released
1607	Caretta caretta	2019	2019-08-12	2019-09-12	18	1	other causes	Miscellany	Released
1608	Caretta caretta	2019	2019-08-12	2019-08-21	20	1	other causes	Miscellany	Released
1609	Caretta caretta	2019	2019-08-12	2019-10-04	20	1	other causes	Miscellany	Released
1610	Caretta caretta	2019	2019-08-12	2019-08-30	39	11	Unknown/undetermined		Released
1611	Caretta caretta	2019	2019-08-12	2019-09-12	24	2	Entanglement		Released
1612	Caretta caretta	2019	2019-08-13	2019-09-12	36	8	Unknown/undetermined		Released
1613	Caretta caretta	2019	2019-08-13	2019-08-30	24	2	Entanglement		Released
1614	Caretta caretta	2019	2019-08-14	2019-09-12	28	3	Entanglement		Released
1615	Caretta caretta	2019	2019-08-17	2019-08-23	29	3	Unknown/undetermined		Died during hospitalization
1616	Caretta caretta	2019	2019-08-19	2019-09-12	16		other causes	Malnutrition	Released
1617	Caretta caretta	2019	2019-08-19				Unknown/undetermined		Admitted dead
1618	Caretta caretta	2019	2019-08-20	2019-08-30	41	8	Unknown/undetermined		Released
1619	Caretta caretta	2019	2019-08-20				Unknown/undetermined		Admitted dead
1620	Caretta caretta	2019	2019-08-21	2019-08-27	55	28	Unknown/undetermined		Released
1621	Caretta caretta	2019	2019-08-22	2019-10-08	32		trauma		Released
1622	Caretta caretta	2019	2019-08-22	2019-10-30		1	Entanglement		Released
1623	Caretta caretta	2019	2019-08-24	2019-10-08	40	8.3	Entanglement		Released
1624	Caretta caretta	2019	2019-08-29	2019-09-12	20	1	Unknown/undetermined		Released
1625	Caretta caretta	2019	2019-08-31		31		Unknown/undetermined		Admitted dead
1626	Caretta caretta	2019	2019-09-01	2019-09-12	33	6	Unknown/undetermined		Released
1627	Caretta caretta	2019	2019-09-03	2019-09-21			Unknown/undetermined		Released
1628	Caretta caretta	2019	2019-09-04	2019-10-30	25	2	Entanglement		Released
1629	Caretta caretta	2019	2019-09-05	2019-10-22		7	Entanglement		Died during hospitalization
1630	Caretta caretta	2019	2019-09-06	2019-09-21	17	0.7	Unknown/undetermined		Released
1631	Caretta caretta	2019	2019-09-08	2019-09-21	18	1	Unknown/undetermined		Released
1632	Caretta caretta	2019	2019-09-11	2019-09-21	18.1	1.1	Unknown/undetermined		Released
1633	Caretta caretta	2019	2019-09-13	2019-09-28	22	1	Unknown/undetermined		Released
1634	Caretta caretta	2019	2019-09-18	2019-10-13	26	2	other causes	Malnutrition	Released
1635	Caretta caretta	2019	2019-09-18	2019-11-20	35	5	other causes	Malnutrition	Died during hospitalization
1636	Caretta caretta	2019	2019-09-18		41	5	Unknown/undetermined		Admitted dead
1637	Caretta caretta	2019	2019-09-19	2019-09-28	40		other causes	Malnutrition	Released
1638	Caretta caretta	2019	2019-09-20	2019-09-28	35		other causes	Malnutrition	Released
1639	Caretta caretta	2019	2019-09-20		36		Unknown/undetermined		Admitted dead
1640	Caretta caretta	2019	2019-09-21		65		trauma		Admitted dead
1641	Caretta caretta	2019	2019-09-21	2019-11-06	25.5	2.6	Entanglement		Released
1642	Caretta caretta	2019	2019-09-24	2019-12-11	22.5	1.6	other causes	Malnutrition	Released
1643	Caretta caretta	2019	2019-09-24	2019-10-05	18	1	Entanglement		Released
1644	Caretta caretta	2019	2019-09-24	2019-09-28	23	2	Entanglement		Released
1645	Caretta caretta	2019	2019-09-24	2019-10-06	30	4.5	Entanglement		Released
1646	Caretta caretta	2019	2019-09-24	2019-10-04	38.5	9.2	Entanglement		Released
1647	Caretta caretta	2019	2019-09-25	2019-10-08	20	1.4	other causes	Malnutrition	Released
1648	Caretta caretta	2019	2019-09-25		21	1.3	Unknown/undetermined		Admitted dead
1649	Caretta caretta	2019	2019-09-25	2019-10-08	35	7.4	Entanglement		Released
1650	Caretta caretta	2019	2019-09-26	2019-12-13	20	1.4	other causes	Malnutrition	Released

Record ID	Species	Year	Admission date	Departure date	SCL (cm)	Body weight (kg)	Cause of admission	Other-cause subcategory	Final disposition
1651	Caretta caretta	2019	2019-09-26	2019-10-04	21	1.5	Entanglement		Released
1652	Caretta caretta	2019	2019-09-27	2019-10-13	23	1.7	other causes	Malnutrition	Released
1653	Caretta caretta	2019	2019-09-27	2019-10-06	25	2.3	Entanglement		Released
1654	Caretta caretta	2019	2019-09-29		34.5		Unknown/undetermined		Admitted dead
1655	Caretta caretta	2019	2019-09-30	2019-12-13	20.9	1.3	Entanglement		Released
1656	Caretta caretta	2019	2019-10-01	2019-10-04	24	2.8	Entanglement		Released
1657	Caretta caretta	2019	2019-10-02	2019-12-19	34	6.3	Entanglement		Released
1658	Caretta caretta	2019	2019-10-08	2019-10-19	48	18	Hooks/monofilament lines		Released
1659	Caretta caretta	2019	2019-10-18	2019-10-30	48	19.4	other causes	Miscellany	Released
1660	Caretta caretta	2019	2019-10-23	2019-11-13	25	2.6	Entanglement		Released
1661	Caretta caretta	2019	2019-10-23	2019-11-07	60	35.1	Entanglement		Released
1662	Caretta caretta	2019	2019-10-31	2019-12-11	32.4	6.1	other causes	Miscellany	Released
1663	Caretta caretta	2019	2019-11-06	2019-11-20	24	2.6	Entanglement		Died during hospitalization
1664	Caretta caretta	2019	2019-11-12	2019-12-11	36.5	8.1	Entanglement		Released
1665	Caretta caretta	2019	2019-11-27	2020-01-09	25.5	2.7	Entanglement		Released
1666	Caretta caretta	2019	2019-12-03	2019-12-13	32	6.5	other causes	Miscellany	Released
1667	Caretta caretta	2019	2019-12-13	2019-12-19	21	1	other causes	Plastic ingestion	Released
1668	Caretta caretta	2019	2019-12-15	2019-12-30	43	13	Entanglement		Released
1669	Caretta caretta	2019	2019-12-16			88	Unknown/undetermined		Admitted dead
1670	Caretta caretta	2019	2019-12-18	2020-01-09	32	7	Infectious disease		Released
1671	Caretta caretta	2019	2019-12-19	2019-12-30	29	4	Unknown/undetermined		Released
1672	Caretta caretta	2019	2019-12-19	2020-01-09	35	8	Unknown/undetermined		Released
1673	Caretta caretta	2019	2019-12-20	2019-12-30	39	9	other causes	Miscellany	Released
1674	Caretta caretta	2019	2019-12-23				Unknown/undetermined		Admitted dead
1675	Caretta caretta	2019	2019-12-25	2020-01-09	47	16	other causes	Plastic ingestion	Released
1676	Caretta caretta	2019	2019-12-26	2020-01-09	29	4	Entanglement		Released
1677	Caretta caretta	2020	2020-01-02		46	16.5	Entanglement		Admitted dead
1678	Caretta caretta	2020	2020-01-03	2020-01-12	58	34.8	Hooks/monofilament lines		Died during hospitalization
1679	Caretta caretta	2020	2020-01-06	2020-01-09			trauma		Released
1680	Caretta caretta	2020	2020-01-12	2020-01-22	23	2.4	other causes	Malnutrition	Released
1681	Caretta caretta	2020	2020-01-13				trauma		Admitted dead
1682	Caretta caretta	2020	2020-01-13	2020-02-10	20.4	1.5	Entanglement		Released
1683	Caretta caretta	2020	2020-01-17	2020-02-10	43	15.3	Infectious disease		Released
1684	Caretta caretta	2020	2020-01-22	2020-02-05	22.1	2	Entanglement		Released
1685	Caretta caretta	2020	2020-01-22	2020-02-05	23.9	2.4	Entanglement		Released
1686	Caretta caretta	2020	2020-01-25	2020-02-20	54	23.6	Unknown/undetermined		Released
1687	Caretta caretta	2020	2020-01-29	2020-02-05	22.5	1.9	Unknown/undetermined		Released
1688	Caretta caretta	2020	2020-02-01		18		Unknown/undetermined		Admitted dead
1689	Caretta caretta	2020	2020-02-02	2020-02-10	20.1	1.4	other causes	Miscellany	Released
1690	Caretta caretta	2020	2020-02-06	2020-02-11	53.8	25.8	Unknown/undetermined		Released
1691	Caretta caretta	2020	2020-02-08	2020-07-09	27	3.1	Entanglement		Released
1692	Caretta caretta	2020	2020-02-08	2020-03-11	28.5	3.6	Entanglement		Released
1693	Caretta caretta	2020	2020-02-09	2020-02-27	27	3.5	Unknown/undetermined		Released
1694	Caretta caretta	2020	2020-02-15		21	1.5	Unknown/undetermined		Admitted dead
1695	Caretta caretta	2020	2020-02-16	2020-02-27	26	3.2	other causes	Miscellany	Released
1696	Caretta caretta	2020	2020-02-20	2020-03-13	57	27	Hooks/monofilament lines		Released
1697	Caretta caretta	2020	2020-02-21				Unknown/undetermined		Admitted dead
1698	Caretta caretta	2020	2020-02-25	2020-03-11	58	28.3	Hooks/monofilament lines		Released
1699	Caretta caretta	2020	2020-02-26	2020-03-05	25.5	2.6	other causes	Malnutrition	Released
1700	Caretta caretta	2020	2020-02-26	2020-03-11	79	70	other causes	Malnutrition	Released
1701	Caretta caretta	2020	2020-02-27				other causes	Malnutrition	Admitted dead
1702	Caretta caretta	2020	2020-03-09	2020-03-31	44	14.7	other causes	Malnutrition	Released
1703	Caretta caretta	2020	2020-03-10	2020-03-31	22.5	2	other causes	Miscellany	Released
1704	Caretta caretta	2020	2020-03-10	2020-03-13	27.5	3.5	other causes	Miscellany	Released
1705	Caretta caretta	2020	2020-03-14				Unknown/undetermined		Admitted dead
1706	Caretta caretta	2020	2020-03-14		49	16.2	Unknown/undetermined		Admitted dead
1707	Caretta caretta	2020	2020-03-14		52	13.1	Unknown/undetermined		Admitted dead
1708	Caretta caretta	2020	2020-04-06				Unknown/undetermined		Admitted dead
1709	Caretta caretta	2020	2020-04-16		50	25	Entanglement		Admitted dead
1710	Caretta caretta	2020	2020-04-27	2020-06-04	44.5	13.8	Entanglement		Released
1711	Caretta caretta	2020	2020-05-02	2020-06-05	23	1.9	Entanglement		Released
1712	Caretta caretta	2020	2020-05-20	2020-06-04	19	1.3	other causes	Miscellany	Released
1713	Caretta caretta	2020	2020-05-21	2020-06-05		2.2	Entanglement		Released
1714	Caretta caretta	2020	2020-05-26	2020-08-05	37.5	8.7	Entanglement		Released
1715	Caretta caretta	2020	2020-05-27	2020-06-04	27	2.9	other causes	Miscellany	Released
1716	Caretta caretta	2020	2020-05-29				Unknown/undetermined		Admitted dead
1717	Caretta caretta	2020	2020-06-02	2020-06-16	25.5	3.2	other causes	Buoyancy disorder	Released
1718	Caretta caretta	2020	2020-06-04	2020-06-16	32.5	5.2	Entanglement		Released
1719	Caretta caretta	2020	2020-06-10				other causes	Buoyancy disorder	Admitted dead
1720	Caretta caretta	2020	2020-06-11	2020-07-24	20.5	1.3	Infectious disease		Released
1721	Caretta caretta	2020	2020-06-12	2020-07-09	34.5	6.8	Entanglement		Released
1722	Caretta caretta	2020	2020-06-13	2020-07-09	24	2.2	other causes	Malnutrition	Released

Record ID	Species	Year	Admission date	Departure date	SCL (cm)	Body weight (kg)	Cause of admission	Other-cause subcategory	Final disposition
1723	Caretta caretta	2020	2020-06-13	2020-07-08	40.5	9.5	Entanglement		Released
1724	Caretta caretta	2020	2020-06-16	2020-08-05	39.5	9.2	Entanglement		Released
1725	Caretta caretta	2020	2020-07-08	2020-07-24	20	1.9	other causes	Malnutrition	Released
1726	Caretta caretta	2020	2020-07-16	2020-09-26	21	1.3	Entanglement		Released
1727	Caretta caretta	2020	2020-07-23	2020-08-14	18.5	1.1	other causes	Malnutrition	Released
1728	Caretta caretta	2020	2020-07-25	2020-09-04	24	1	other causes	Malnutrition	Released
1729	Caretta caretta	2020	2020-07-25	2020-08-07	23	2	Entanglement		Released
1730	Caretta caretta	2020	2020-07-27	2020-08-06	22.5	1.9	Entanglement		Died during hospitalization
1731	Caretta caretta	2020	2020-07-28				Unknown/undetermined		Admitted dead
1732	Caretta caretta	2020	2020-07-29				Unknown/undetermined		Admitted dead
1733	Caretta caretta	2020	2020-08-03				Unknown/undetermined		Admitted dead
1734	Caretta caretta	2020	2020-08-08	2020-09-26	17.5	0.8	Entanglement		Released
1735	Caretta caretta	2020	2020-08-08	2020-10-22	37.3	9.1	Entanglement		Released
1736	Caretta caretta	2020	2020-08-13		73	51.5	Entanglement		Admitted dead
1737	Caretta caretta	2020	2020-08-15	2020-10-02	20.5	1.4	Entanglement		Released
1738	Caretta caretta	2020	2020-08-15	2020-10-15	38.5	10.5	Entanglement		Died during hospitalization
1739	Caretta caretta	2020	2020-08-19	2020-09-04	18	1.2	other causes	Malnutrition	Released
1740	Caretta caretta	2020	2020-08-25	2020-11-09	22	1.8	Entanglement		Released
1741	Caretta caretta	2020	2020-08-26	2020-09-04	26	2.9	Entanglement		Released
1742	Caretta caretta	2020	2020-08-29		18		Unknown/undetermined		Admitted dead
1743	Caretta caretta	2020	2020-08-30	2020-09-04	20	1.4	other causes	Malnutrition	Released
1744	Caretta caretta	2020	2020-08-31	2020-10-22	22.5	2.7	Entanglement		Released
1745	Caretta caretta	2020	2020-08-31	2020-09-24	46.5	14.4	Entanglement		Released
1746	Caretta caretta	2020	2020-09-01	2020-11-09	38	8.6	Entanglement		Released
1747	Caretta caretta	2020	2020-09-27				Unknown/undetermined		Admitted dead
1748	Caretta caretta	2020	2020-10-02				Unknown/undetermined		Admitted dead
1749	Caretta caretta	2020	2020-10-07	2020-11-05	69	48.8	Hooks/monofilament lines		Released
1750	Caretta caretta	2020	2020-10-10				Unknown/undetermined		Admitted dead
1751	Caretta caretta	2020	2020-10-12	2020-12-02	50		Unknown/undetermined		Released
1752	Caretta caretta	2020	2020-10-12		32	5.6	Unknown/undetermined		Admitted dead
1753	Caretta caretta	2020	2020-10-15		38	10	Unknown/undetermined		Admitted dead
1754	Caretta caretta	2020	2020-10-18	2020-11-05	56	29.4	Entanglement		Released
1755	Caretta caretta	2020	2020-10-26				Unknown/undetermined		Admitted dead
1756	Caretta caretta	2020	2020-10-27	2021-05-30	31.2	4.5	other causes	Malnutrition	Released
1757	Caretta caretta	2020	2020-11-05	2021-08-06	51	18.6	other causes	Malnutrition	Released
1758	Caretta caretta	2020	2020-12-24		55		Unknown/undetermined		Admitted dead
1759	Caretta caretta	2020	2020-12-29		35		Unknown/undetermined		Admitted dead
1760	Caretta caretta	2021	2021-01-14	2021-03-23	67	41	other causes	Malnutrition	Died during hospitalization
1761	Caretta caretta	2021	2021-01-23	2021-05-14	40.5	8.9	Infectious disease		Released
1762	Caretta caretta	2021	2021-01-30	2021-01-30	63	40	trauma		Died during hospitalization
1763	Caretta caretta	2021	2021-01-31		27	3	trauma		Admitted dead
1764	Caretta caretta	2021	2021-02-01	2021-03-13	24.5	2.7	Entanglement		Released
1765	Caretta caretta	2021	2021-02-02	2021-02-26	28	3.4	Entanglement		Released
1766	Caretta caretta	2021	2021-02-17				Unknown/undetermined		Admitted dead
1767	Caretta caretta	2021	2021-02-18	2021-08-26	41.5	10.9	Infectious disease		Released
1768	Caretta caretta	2021	2021-03-05	2021-05-12	37.9	9.2	Entanglement		Released
1769	Caretta caretta	2021	2021-03-18	2021-03-19	26	2.2	other causes	Malnutrition	Died during hospitalization
1770	Caretta caretta	2021	2021-03-20	2021-05-12	35	7.4	Infectious disease		Released
1771	Caretta caretta	2021	2021-03-22		70	65	trauma		Admitted dead
1772	Caretta caretta	2021	2021-03-27		80	75	trauma		Admitted dead
1773	Caretta caretta	2021	2021-03-27		22.5		Unknown/undetermined		Admitted dead
1774	Caretta caretta	2021	2021-03-27		28		Unknown/undetermined		Admitted dead
1775	Caretta caretta	2021	2021-04-01		75	40	Unknown/undetermined		Admitted dead
1776	Caretta caretta	2021	2021-04-03	2021-04-04	26.2	2.7	other causes	Malnutrition	Died during hospitalization
1777	Caretta caretta	2021	2021-04-05	2021-05-14	60	35	Hooks/monofilament lines		Released
1778	Caretta caretta	2021	2021-04-05		58	35	trauma		Admitted dead
1779	Caretta caretta	2021	2021-04-09		42	12	Unknown/undetermined		Admitted dead
1780	Caretta caretta	2021	2021-04-12		48.5	18	Entanglement		Admitted dead
1781	Caretta caretta	2021	2021-04-14	2021-05-01	49	21.9	Infectious disease		Released
1782	Caretta caretta	2021	2021-04-14	2021-05-26	23.5	2.2	Entanglement		Released
1783	Caretta caretta	2021	2021-04-16	2021-08-06	39.5	8.2	Infectious disease		Released
1784	Caretta caretta	2021	2021-04-17	2021-07-02	28	3.1	Infectious disease		Released
1785	Caretta caretta	2021	2021-04-21	2021-05-29	19	1.2	Infectious disease		Released
1786	Caretta caretta	2021	2021-05-01		72	37	Unknown/undetermined		Admitted dead
1787	Caretta caretta	2021	2021-05-13		38	5	trauma		Admitted dead
1788	Caretta caretta	2021	2021-05-25	2021-06-05	27	3.1	Entanglement		Released
1789	Caretta caretta	2021	2021-05-28	2021-06-18	31.8	6.2	Infectious disease		Released
1790	Caretta caretta	2021	2021-06-05	2021-07-13	30	4.1	Entanglement		Released
1791	Caretta caretta	2021	2021-06-13	2021-07-06			Entanglement		Released
1792	Caretta caretta	2021	2021-06-13	2021-07-28			Entanglement		Released
1793	Caretta caretta	2021	2021-06-14				Unknown/undetermined		Admitted dead
1794	Caretta caretta	2021	2021-06-16	2021-07-19			Unknown/undetermined		Released

Record ID	Species	Year	Admission date	Departure date	SCL (cm)	Body weight (kg)	Cause of admission	Other-cause subcategory	Final disposition
1795	Caretta caretta	2021	2021-06-21				Unknown/undetermined		Admitted dead
1796	Caretta caretta	2021	2021-06-23				Unknown/undetermined		Admitted dead
1797	Caretta caretta	2021	2021-06-23	2021-09-16	37	8	Infectious disease		Released
1798	Caretta caretta	2021	2021-06-25		36	8	trauma		Admitted dead
1799	Caretta caretta	2021	2021-06-26	2021-06-30	49	15	Hooks/monofilament lines		Died during hospitalization
1800	Caretta caretta	2021	2021-06-26		37	8	Unknown/undetermined		Admitted dead
1801	Caretta caretta	2021	2021-06-27	2021-06-28	25	2	Entanglement		Died during hospitalization
1802	Caretta caretta	2021	2021-06-28		25	2	other causes	Malnutrition	Admitted dead
1803	Caretta caretta	2021	2021-06-30				Unknown/undetermined		Admitted dead
1804	Caretta caretta	2021	2021-07-01				Unknown/undetermined		Admitted dead
1805	Caretta caretta	2021	2021-07-08	2021-08-26	20	1	other causes	Malnutrition	Released
1806	Caretta caretta	2021	2021-07-09		45		Unknown/undetermined		Admitted dead
1807	Caretta caretta	2021	2021-07-12	2021-07-19	50	18	Hooks/monofilament lines		Released
1808	Caretta caretta	2021	2021-07-12	2021-07-21	44	14	Unknown/undetermined		Released
1809	Caretta caretta	2021	2021-07-17		27	2	trauma		Admitted dead
1810	Caretta caretta	2021	2021-07-17	2021-08-06	22	1	other causes	Miscellany	Released
1811	Caretta caretta	2021	2021-07-17		23	1	Unknown/undetermined		Admitted dead
1812	Caretta caretta	2021	2021-07-20	2021-07-28	30	4	other causes	Miscellany	Released
1813	Caretta caretta	2021	2021-07-24	2021-08-10	19	1	other causes	Buoyancy disorder	Released
1814	Caretta caretta	2021	2021-07-24	2021-07-30	25	2	other causes	Buoyancy disorder	Released
1815	Caretta caretta	2021	2021-07-27	2021-07-30	32	6	other causes	Buoyancy disorder	Released
1816	Caretta caretta	2021	2021-07-31	2021-08-13	25	2	other causes	Miscellany	Released
1817	Caretta caretta	2021	2021-08-01	2021-08-12	25	2	other causes	Miscellany	Released
1818	Caretta caretta	2021	2021-08-04		36		Unknown/undetermined		Admitted dead
1819	Caretta caretta	2021	2021-08-05	2021-09-17	46	17	Infectious disease		Released
1820	Caretta caretta	2021	2021-08-06	2021-08-20	22	1	Infectious disease		Released
1821	Caretta caretta	2021	2021-08-09	2021-08-20	20	1	Infectious disease		Released
1822	Caretta caretta	2021	2021-08-15		42		trauma		Admitted dead
1823	Caretta caretta	2021	2021-08-15	2021-10-01	26	2	Entanglement		Released
1824	Caretta caretta	2021	2021-08-17	2021-11-05	27	3	Infectious disease		Released
1825	Caretta caretta	2021	2021-08-18	2021-10-07	30	4	Entanglement		Released
1826	Caretta caretta	2021	2021-08-20	2021-08-27	46	14	other causes	Buoyancy disorder	Released
1827	Caretta caretta	2021	2021-08-23		56	26	Unknown/undetermined		Admitted dead
1828	Caretta caretta	2021	2021-08-24	2021-09-01	20	1	other causes	Malnutrition	Died during hospitalization
1829	Caretta caretta	2021	2021-08-25	2021-09-07	22	1	other causes	Buoyancy disorder	Released
1830	Caretta caretta	2021	2021-09-01		54		Unknown/undetermined		Admitted dead
1831	Caretta caretta	2021	2021-09-02		20	0.8	Unknown/undetermined		Admitted dead
1832	Caretta caretta	2021	2021-09-04	2021-09-26	26	3	other causes	Miscellany	Released
1833	Caretta caretta	2021	2021-09-07	2021-10-14	33	4	Crude oil		Released
1834	Caretta caretta	2021	2021-09-13	2021-09-25	22	1	Infectious disease		Released
1835	Caretta caretta	2021	2021-09-15	2021-09-22	61	39	Hooks/monofilament lines		Died during hospitalization
1836	Caretta caretta	2021	2021-10-09			25	Unknown/undetermined		Admitted dead
1837	Caretta caretta	2021	2021-10-12	2021-11-04	35	2	Entanglement		Released
1838	Caretta caretta	2021	2021-10-12		75	24	Entanglement		Admitted dead
1839	Caretta caretta	2021	2021-10-15		51	7	Hooks/monofilament lines		Admitted dead
1840	Caretta caretta	2021	2021-10-15		23	2	Unknown/undetermined		Admitted dead
1841	Caretta caretta	2021	2021-10-20	2021-11-26			Entanglement		Released
1842	Caretta caretta	2021	2021-10-30		36	4.8	trauma		Admitted dead
1843	Caretta caretta	2021	2021-11-08	2021-11-12	40	10	Entanglement		Released
1844	Caretta caretta	2021	2021-11-10	2021-11-16	36	5	Entanglement		Died during hospitalization
1845	Caretta caretta	2021	2021-11-14	2022-03-08	35.8	6.4	Entanglement		Released
1846	Caretta caretta	2021	2021-11-27	2022-01-04	45	14.7	Infectious disease		Released
1847	Caretta caretta	2021	2021-12-20	2021-12-30	22.7	2	other causes	Malnutrition	Released
1848	Caretta caretta	2022	2022-01-07	2022-01-28	39	9.7	other causes	Buoyancy disorder	Released
1849	Caretta caretta	2022	2022-01-11	2022-03-25	35	6.5	trauma		Released
1850	Caretta caretta	2022	2022-01-12	2022-02-07	42.3	12.6	other causes	Buoyancy disorder	Released
1851	Caretta caretta	2022	2022-01-12	2022-02-08	51.5	19.6	Infectious disease		Released
1852	Caretta caretta	2022	2022-01-27	2022-05-09	24.8	2	Entanglement		Released
1853	Caretta caretta	2022	2022-01-31	2022-02-18	52.2	21.6	other causes	Miscellany	Released
1854	Caretta caretta	2022	2022-02-12	2022-04-27	36.5	7.3	Entanglement		Released
1855	Caretta caretta	2022	2022-02-17	2022-02-23	59.5	33	Infectious disease		Released
1856	Caretta caretta	2022	2022-02-23	2022-04-11	29.9	3.9	Entanglement		Released
1857	Caretta caretta	2022	2022-03-02	2022-03-08	59.8	26	Hooks/monofilament lines		Died during hospitalization
1858	Caretta caretta	2022	2022-03-08	2022-03-24	43.5	15.7	other causes	Miscellany	Released
1859	Caretta caretta	2022	2022-03-10	2022-03-18	38.1	9.7	other causes	Malnutrition	Released
1860	Caretta caretta	2022	2022-03-11	2022-03-16	37.5	7	Entanglement		Died during hospitalization
1861	Caretta caretta	2022	2022-04-07	2022-05-13	31	5.3	Entanglement		Released
1862	Caretta caretta	2022	2022-04-08		55		trauma		Admitted dead
1863	Caretta caretta	2022	2022-04-09	2022-04-09	27.3	3.6	Infectious disease		Died during hospitalization
1864	Caretta caretta	2022	2022-04-28	2022-07-02	32.4	5.9	Entanglement		Released
1865	Caretta caretta	2022	2022-04-29	2022-11-28	52		trauma		Released
1866	Caretta caretta	2022	2022-05-09	2022-05-13	43.5		other causes	Buoyancy disorder	Released

Record ID	Species	Year	Admission date	Departure date	SCL (cm)	Body weight (kg)	Cause of admission	Other-cause subcategory	Final disposition
1867	Caretta caretta	2022	2022-05-12	2022-05-20	45	13.4	other causes	Buoyancy disorder	Released
1868	Caretta caretta	2022	2022-05-13				trauma		Admitted dead
1869	Caretta caretta	2022	2022-05-15	2022-05-22	36	6.7	other causes	Buoyancy disorder	Released
1870	Caretta caretta	2022	2022-05-18	2022-05-26	41.9	11.4	other causes	Miscellany	Released
1871	Caretta caretta	2022	2022-05-20				Entanglement		Admitted dead
1872	Caretta caretta	2022	2022-05-22	2022-06-28	23.5	2.2	Entanglement		Released
1873	Caretta caretta	2022	2022-05-22	2022-06-28	27	3	Entanglement		Released
1874	Caretta caretta	2022	2022-05-22	2022-06-13	41.5	10.3	Entanglement		Released
1875	Caretta caretta	2022	2022-05-23	2022-05-30	28	3.5	Entanglement		Released
1876	Caretta caretta	2022	2022-05-23	2022-06-13	35	6	Entanglement		Released
1877	Caretta caretta	2022	2022-05-23	2022-07-14	42.3	9.5	Entanglement		Released
1878	Caretta caretta	2022	2022-05-23	2022-07-13	43.5	10.8	Entanglement		Released
1879	Caretta caretta	2022	2022-05-27	2022-05-30	24.2	2.4	Unknown/undetermined		Released
1880	Caretta caretta	2022	2022-05-27	2022-06-22	17	0.9	Infectious disease		Released
1881	Caretta caretta	2022	2022-05-30	2022-06-05	44	12.2	Unknown/undetermined		Released
1882	Caretta caretta	2022	2022-06-04				Unknown/undetermined		Admitted dead
1883	Caretta caretta	2022	2022-06-09	2022-06-22	19.8	1.1	other causes	Miscellany	Released
1884	Caretta caretta	2022	2022-06-10	2022-06-28	36.5	6.3	other causes	Miscellany	Released
1885	Caretta caretta	2022	2022-06-16				Unknown/undetermined		Admitted dead
1886	Caretta caretta	2022	2022-06-19	2022-06-28	31.5	4.5	Entanglement		Released
1887	Caretta caretta	2022	2022-06-23	2022-06-28	44.7	15.4	other causes	Miscellany	Released
1888	Caretta caretta	2022	2022-06-23	2022-07-01	37.6	6	Entanglement		Died during hospitalization
1889	Caretta caretta	2022	2022-07-02	2022-07-22	29.9	4.9	Entanglement		Released
1890	Caretta caretta	2022	2022-07-04	2022-07-13	34.5	6.5	Entanglement		Released
1891	Caretta caretta	2022	2022-07-08				Unknown/undetermined		Admitted dead
1892	Caretta caretta	2022	2022-07-09		51		Entanglement		Admitted dead
1893	Caretta caretta	2022	2022-07-13	2022-07-26	18.5	1.4	other causes	Miscellany	Released
1894	Caretta caretta	2022	2022-07-15	2022-07-26	16.5	1	other causes	Miscellany	Released
1895	Caretta caretta	2022	2022-07-15	2022-07-22	33	5.7	Entanglement		Released
1896	Caretta caretta	2022	2022-07-18	2022-07-26	18.5	1.2	other causes	Buoyancy disorder	Released
1897	Caretta caretta	2022	2022-07-18	2022-08-07	30.5	4.5	Entanglement		Released
1898	Caretta caretta	2022	2022-07-19	2022-07-29	45.5	14.1	other causes	Buoyancy disorder	Released
1899	Caretta caretta	2022	2022-07-19	2022-08-12	39	7.2	Entanglement		Released
1900	Caretta caretta	2022	2022-07-20	2022-08-16	17.5	0.9	other causes	Buoyancy disorder	Released
1901	Caretta caretta	2022	2022-07-24	2022-07-31	28	3.5	Unknown/undetermined		Released
1902	Caretta caretta	2022	2022-07-26	2022-08-07	18	0.9	Entanglement		Released
1903	Caretta caretta	2022	2022-07-29	2022-08-07			other causes	Buoyancy disorder	Released
1904	Caretta caretta	2022	2022-07-29	2022-08-07			other causes	Miscellany	Released
1905	Caretta caretta	2022	2022-07-29	2022-08-16	31	4.8	Entanglement		Released
1906	Caretta caretta	2022	2022-07-30	2022-07-31		9.8	Hooks/monofilament lines		Died during hospitalization
1907	Caretta caretta	2022	2022-07-30	2022-08-07	22	2.1	other causes	Buoyancy disorder	Released
1908	Caretta caretta	2022	2022-07-31	2022-09-06	22	1.9	Entanglement		Released
1909	Caretta caretta	2022	2022-07-31			6.2	Entanglement		Admitted dead
1910	Caretta caretta	2022	2022-08-06	2022-08-23	26.8	2.7	Entanglement		Released
1911	Caretta caretta	2022	2022-08-06	2022-08-19	36.5	7.4	Entanglement		Released
1912	Caretta caretta	2022	2022-08-09	2022-09-01	20.1	1.4	other causes	Buoyancy disorder	Released
1913	Caretta caretta	2022	2022-08-09	2022-08-23	22.5	2.2	other causes	Buoyancy disorder	Released
1914	Caretta caretta	2022	2022-08-13	2022-08-18	43.2	11.8	other causes	Buoyancy disorder	Released
1915	Caretta caretta	2022	2022-08-13	2022-08-23		2.4	Entanglement		Died during hospitalization
1916	Caretta caretta	2022	2022-08-15	2022-09-23	31.3	5.5	Entanglement		Released
1917	Caretta caretta	2022	2022-08-15				Entanglement		Admitted dead
1918	Caretta caretta	2022	2022-08-18				Unknown/undetermined		Admitted dead
1919	Caretta caretta	2022	2022-08-22	2022-10-18	44.5	10.8	Infectious disease		Released
1920	Caretta caretta	2022	2022-08-25	2022-09-06	27.8	3.4	Entanglement		Released
1921	Caretta caretta	2022	2022-08-27		27.8	3.4	Unknown/undetermined		Admitted dead
1922	Caretta caretta	2022	2022-08-28	2022-09-19	30	4.2	Entanglement		Released
1923	Caretta caretta	2022	2022-09-01	2022-09-19	28	3.3	Entanglement		Released
1924	Caretta caretta	2022	2022-09-05		44	12.2	Unknown/undetermined		Admitted dead
1925	Caretta caretta	2022	2022-09-08	2022-10-10	19.5	1.2	Infectious disease		Released
1926	Caretta caretta	2022	2022-09-08	2022-09-27	29.2	4.2	Infectious disease		Released
1927	Caretta caretta	2022	2022-09-08	2022-09-27	31	4.4	Entanglement		Released
1928	Caretta caretta	2022	2022-09-09				Hooks/monofilament lines		Admitted dead
1929	Caretta caretta	2022	2022-09-11				Unknown/undetermined		Admitted dead
1930	Caretta caretta	2022	2022-09-16				Unknown/undetermined		Admitted dead
1931	Caretta caretta	2022	2022-09-17	2022-10-18	58	26.5	Entanglement		Released
1932	Caretta caretta	2022	2022-09-19	2022-10-11			Entanglement		Released
1933	Caretta caretta	2022	2022-09-20	2022-09-29	16.5	0.5	other causes	Miscellany	Released
1934	Caretta caretta	2022	2022-09-20	2022-10-10	17	0.9	Unknown/undetermined		Released
1935	Caretta caretta	2022	2022-09-20				Entanglement		Admitted dead
1936	Caretta caretta	2022	2022-09-20		54.8	30	Entanglement		Admitted dead
1937	Caretta caretta	2022	2022-09-23		48	15.4	Unknown/undetermined		Admitted dead
1938	Caretta caretta	2022	2022-10-01	2022-10-21	31.5	5.4	Entanglement		Released

Record ID	Species	Year	Admission date	Departure date	SCL (cm)	Body weight (kg)	Cause of admission	Other-cause subcategory	Final disposition
1939	Caretta caretta	2022	2022-10-09	2022-10-11	37.2	7.5	Entanglement		Released
1940	Caretta caretta	2022	2022-10-21	2022-10-27	18.5	1.2	other causes	Miscellany	Released
1941	Caretta caretta	2022	2022-10-23	2022-11-18	25.1	2.4	Entanglement		Released
1942	Caretta caretta	2022	2022-11-02	2022-11-18			Entanglement		Released
1943	Caretta caretta	2022	2022-11-07	2022-12-01	22.1	1.7	Entanglement		Released
1944	Caretta caretta	2022	2022-11-13		51	24	other causes	Buoyancy disorder	Admitted dead
1945	Caretta caretta	2022	2022-11-16	2022-12-07	31	4.5	Entanglement		Released
1946	Caretta caretta	2022	2022-11-18	2022-12-07	23	1.9	Entanglement		Released
1947	Caretta caretta	2022	2022-11-22	2022-11-28	33	5.3	Entanglement		Released
1948	Caretta caretta	2022	2022-12-01		37.5	10	Entanglement		Admitted dead
1949	Caretta caretta	2022	2022-12-08	2022-12-14	61	33	Hooks/monofilament lines		Released
1950	Caretta caretta	2022	2022-12-08				Unknown/undetermined		Admitted dead
1951	Caretta caretta	2023	2023-01-08	2023-01-08	65	49	trauma		Died during hospitalization
1952	Caretta caretta	2023	2023-01-09				Unknown/undetermined		Admitted dead
1953	Caretta caretta	2023	2023-01-18	2023-02-03	26	2	Entanglement		Died during hospitalization
1954	Caretta caretta	2023	2023-01-20				Unknown/undetermined		Admitted dead
1955	Caretta caretta	2023	2023-01-20	2023-02-22	32.8	5.1	Entanglement		Died during hospitalization
1956	Caretta caretta	2023	2023-02-06		50		Entanglement		Admitted dead
1957	Caretta caretta	2023	2023-02-07				Unknown/undetermined		Admitted dead
1958	Caretta caretta	2023	2023-02-19	2023-03-24	38	8	Entanglement		Released
1959	Caretta caretta	2023	2023-02-22	2023-04-04	34	6	other causes	Miscellany	Released
1960	Caretta caretta	2023	2023-02-23	2023-03-09	34	6	Unknown/undetermined		Released
1961	Caretta caretta	2023	2023-03-07	2023-03-30	42	11	Infectious disease		Released
1962	Caretta caretta	2023	2023-03-11	2023-03-30	34	7	Entanglement		Released
1963	Caretta caretta	2023	2023-03-12	2023-03-20	20		other causes	Malnutrition	Released
1964	Caretta caretta	2023	2023-03-15				Unknown/undetermined		Admitted dead
1965	Caretta caretta	2023	2023-03-27		33	4.9	Unknown/undetermined		Admitted dead
1966	Caretta caretta	2023	2023-03-28	2023-04-04	21	1.7	other causes	Buoyancy disorder	Released
1967	Caretta caretta	2023	2023-03-29		29	3	Unknown/undetermined		Admitted dead
1968	Caretta caretta	2023	2023-04-06	2023-04-24	35	7	other causes	Buoyancy disorder	Released
1969	Caretta caretta	2023	2023-04-06	2023-04-21	37	8.3	Unknown/undetermined		Released
1970	Caretta caretta	2023	2023-04-09	2023-04-21	28.5	3	Entanglement		Released
1971	Caretta caretta	2023	2023-04-20	2023-05-13	23.5	2.1	Entanglement		Died during hospitalization
1972	Caretta caretta	2023	2023-04-26	2023-05-19	24	2.3	other causes	Buoyancy disorder	Released
1973	Caretta caretta	2023	2023-04-27	2023-05-12	34	6.8	Unknown/undetermined		Released
1974	Caretta caretta	2023	2023-05-03	2023-06-04	31	4.2	other causes	Malnutrition	Released
1975	Caretta caretta	2023	2023-05-04	2023-08-19	26.5	2	other causes	Malnutrition	Released
1976	Caretta caretta	2023	2023-05-06	2023-06-09	30	5.4	Entanglement		Released
1977	Caretta caretta	2023	2023-05-08	2023-05-19	27	2.5	Unknown/undetermined		Released
1978	Caretta caretta	2023	2023-05-08				Unknown/undetermined		Admitted dead
1979	Caretta caretta	2023	2023-05-09	2023-05-27	29.5	3	Entanglement		Released
1980	Caretta caretta	2023	2023-05-09	2023-05-27	34	6	Entanglement		Released
1981	Caretta caretta	2023	2023-05-11	2023-06-02	40	10	Unknown/undetermined		Released
1982	Caretta caretta	2023	2023-05-13	2023-07-21	38.5	9	Entanglement		Released
1983	Caretta caretta	2023	2023-05-20				Unknown/undetermined		Admitted dead
1984	Caretta caretta	2023	2023-05-24	2023-06-30	50.5	18.5	Entanglement		Released
1985	Caretta caretta	2023	2023-05-26		23	2.5	Unknown/undetermined		Admitted dead
1986	Caretta caretta	2023	2023-05-26	2023-06-02	33	5	Entanglement		Released
1987	Caretta caretta	2023	2023-05-28		72		Unknown/undetermined		Admitted dead
1988	Caretta caretta	2023	2023-05-29	2023-12-22	41	14.7	trauma		Released
1989	Caretta caretta	2023	2023-06-03	2023-06-13	34.5	6.8	Entanglement		Released
1990	Caretta caretta	2023	2023-06-03	2023-06-13	36	7.3	Entanglement		Released
1991	Caretta caretta	2023	2023-06-15	2023-07-04	23	1.6	Entanglement		Released
1992	Caretta caretta	2023	2023-06-20		65	60	Unknown/undetermined		Admitted dead
1993	Caretta caretta	2023	2023-06-21	2023-11-06	30.5	3.9	other causes	Malnutrition	Released
1994	Caretta caretta	2023	2023-06-24	2023-06-30	24	2.5	Entanglement		Released
1995	Caretta caretta	2023	2023-06-28		43	4	Unknown/undetermined		Admitted dead
1996	Caretta caretta	2023	2023-07-01				Unknown/undetermined		Admitted dead
1997	Caretta caretta	2023	2023-07-03	2023-07-17	23.5	2.2	Unknown/undetermined		Released
1998	Caretta caretta	2023	2023-07-06	2023-07-26	22.5	1.9	other causes	Buoyancy disorder	Released
1999	Caretta caretta	2023	2023-07-06	2023-07-26	27	2.2	Entanglement		Released
2000	Caretta caretta	2023	2023-07-06	2023-07-21	37	6.9	Entanglement		Released
2001	Caretta caretta	2023	2023-07-08	2023-07-25	20	0.9	other causes	Malnutrition	Died during hospitalization
2002	Caretta caretta	2023	2023-07-22	2023-07-26	40	8.8	Unknown/undetermined		Released
2003	Caretta caretta	2023	2023-07-23		64	46	Unknown/undetermined		Admitted dead
2004	Caretta caretta	2023	2023-07-23	2023-08-31	24	2.1	Entanglement		Released
2005	Caretta caretta	2023	2023-07-26				Unknown/undetermined		Admitted dead
2006	Caretta caretta	2023	2023-07-27	2023-08-31	26	2.7	Entanglement		Released
2007	Caretta caretta	2023	2023-08-01	2023-08-19	41.5	12.8	other causes	Malnutrition	Released
2008	Caretta caretta	2023	2023-08-02	2023-08-31	25.9	2.6	Entanglement		Released
2009	Caretta caretta	2023	2023-08-21	2023-09-09	30	3.8	Entanglement		Released
2010	Caretta caretta	2023	2023-08-24	2023-09-23	19.5	1.2	Entanglement		Released

Record ID	Species	Year	Admission date	Departure date	SCL (cm)	Body weight (kg)	Cause of admission	Other-cause subcategory	Final disposition
2011	Caretta caretta	2023	2023-08-29	2023-10-14	17	0.7	Entanglement		Released
2012	Caretta caretta	2023	2023-08-30	2023-09-09	40	12.7	other causes	Buoyancy disorder	Released
2013	Caretta caretta	2023	2023-08-31	2023-10-10	45.5	16.6	other causes	Buoyancy disorder	Released
2014	Caretta caretta	2023	2023-09-02				Unknown/undetermined		Admitted dead
2015	Caretta caretta	2023	2023-09-03	2023-09-04	25.5	1.9	Entanglement		Died during hospitalization
2016	Caretta caretta	2023	2023-09-04	2023-09-17	22.5	1.8	Unknown/undetermined		Released
2017	Caretta caretta	2023	2023-09-05	2023-10-10			other causes	Buoyancy disorder	Released
2018	Caretta caretta	2023	2023-09-07	2023-11-25	29	2.9	Entanglement		Released
2019	Caretta caretta	2023	2023-09-11	2023-10-17	38	8.2	Entanglement		Released
2020	Caretta caretta	2023	2023-09-12	2023-09-27	19.5	1.1	other causes	Malnutrition	Released
2021	Caretta caretta	2023	2023-09-12	2023-11-07	35	6.2	other causes	Buoyancy disorder	Released
2022	Caretta caretta	2023	2023-09-13		34	5.2	Unknown/undetermined		Admitted dead
2023	Caretta caretta	2023	2023-09-13		60	28	Unknown/undetermined		Admitted dead
2024	Caretta caretta	2023	2023-09-16		27	2.6	Unknown/undetermined		Admitted dead
2025	Caretta caretta	2023	2023-09-19				Unknown/undetermined		Admitted dead
2026	Caretta caretta	2023	2023-09-21				Unknown/undetermined		Admitted dead
2027	Caretta caretta	2023	2023-09-23	2023-10-30	30	3.6	Crude oil		Released
2028	Caretta caretta	2023	2023-09-24				Unknown/undetermined		Admitted dead
2029	Caretta caretta	2023	2023-09-24				Unknown/undetermined		Admitted dead
2030	Caretta caretta	2023	2023-09-26				Unknown/undetermined		Admitted dead
2031	Caretta caretta	2023	2023-09-28	2024-06-10	37.5	7.3	Unknown/undetermined		Released
2032	Caretta caretta	2023	2023-09-29	2024-03-14	26.5	2.3	Unknown/undetermined		Released
2033	Caretta caretta	2023	2023-09-29	2023-09-30	34	3.6	Unknown/undetermined		Died during hospitalization
2034	Caretta caretta	2023	2023-10-02			6	Unknown/undetermined		Admitted dead
2035	Caretta caretta	2023	2023-10-05	2024-01-15	33.5	4.8	Infectious disease		Released
2036	Caretta caretta	2023	2023-10-08				Unknown/undetermined		Admitted dead
2037	Caretta caretta	2023	2023-10-11	2023-10-20	27	2.7	Unknown/undetermined		Released
2038	Caretta caretta	2023	2023-10-14				Unknown/undetermined		Admitted dead
2039	Caretta caretta	2023	2023-10-14		57	34.7	Unknown/undetermined		Admitted dead
2040	Caretta caretta	2023	2023-10-14		62	42	Unknown/undetermined		Admitted dead
2041	Caretta caretta	2023	2023-10-19	2023-10-30	24	1.9	Entanglement		Released
2042	Caretta caretta	2023	2023-10-20	2023-12-12	74	40	Entanglement		Released
2043	Caretta caretta	2023	2023-10-21	2024-04-18	26.5	2.5	Infectious disease		Released
2044	Caretta caretta	2023	2023-10-29	2023-12-12	47.5	14.7	other causes	Shark attack	Released
2045	Caretta caretta	2023	2023-11-05		56	25.1	Unknown/undetermined		Admitted dead
2046	Caretta caretta	2023	2023-11-12	2024-03-14			Unknown/undetermined		Released
2047	Caretta caretta	2023	2023-11-18	2023-11-27	24	1.7	Entanglement		Died during hospitalization
2048	Caretta caretta	2023	2023-11-20	2024-04-18	30.5	3.8	other causes	Malnutrition	Released
2049	Caretta caretta	2023	2023-11-29	2024-01-29	28	3.2	Entanglement		Released
2050	Caretta caretta	2023	2023-12-01			15	Entanglement		Admitted dead
2051	Caretta caretta	2023	2023-12-07	2023-12-13	20.5	1.2	Entanglement		Released
2052	Caretta caretta	2023	2023-12-18		50	20	Entanglement		Admitted dead
2053	Caretta caretta	2024	2024-02-07		51.5	18	Unknown/undetermined		Admitted dead
2054	Caretta caretta	2024	2024-02-14	2024-02-20	49.5	18.7	other causes	Miscellany	Released
2055	Caretta caretta	2024	2024-02-22	2024-04-04	22	2	Entanglement		Released
2056	Caretta caretta	2024	2024-02-23	2024-03-13	40	9.7	other causes	Miscellany	Released
2057	Caretta caretta	2024	2024-03-06	2024-04-10	35	6.4	Entanglement		Died during hospitalization
2058	Caretta caretta	2024	2024-03-08	2024-04-18	35	7.8	Entanglement		Released
2059	Caretta caretta	2024	2024-03-22	2024-03-23	22.5	1.6	Entanglement		Died during hospitalization
2060	Caretta caretta	2024	2024-03-23	2024-05-02	39	8.2	Entanglement		Released
2061	Caretta caretta	2024	2024-03-27				Unknown/undetermined		Admitted dead
2062	Caretta caretta	2024	2024-04-01	2024-04-30	22.5	2.5	Entanglement		Released
2063	Caretta caretta	2024	2024-04-04		33.5	4.1	Unknown/undetermined		Admitted dead
2064	Caretta caretta	2024	2024-04-05	2024-04-30	28	3.6	Entanglement		Released
2065	Caretta caretta	2024	2024-04-06	2024-04-12	54	18.8	Hooks/monofilament lines		Died during hospitalization
2066	Caretta caretta	2024	2024-04-09	2024-05-28	20.5	1.2	Entanglement		Released
2067	Caretta caretta	2024	2024-04-15	2024-05-14	28.5		other causes	Malnutrition	Released
2068	Caretta caretta	2024	2024-04-16	2024-06-30	32.5	4.7	Infectious disease		Released
2069	Caretta caretta	2024	2024-04-17	2024-07-25	27.5	2.6	Unknown/undetermined		Released
2070	Caretta caretta	2024	2024-04-17		29	2.8	Unknown/undetermined		Admitted dead
2071	Caretta caretta	2024	2024-04-23	2024-05-14	36	7	Entanglement		Released
2072	Caretta caretta	2024	2024-05-06	2024-05-17	40.5	11	other causes	Buoyancy disorder	Released
2073	Caretta caretta	2024	2024-05-08	2024-05-28	26.5	3.1	Entanglement		Released
2074	Caretta caretta	2024	2024-05-13	2024-06-10	19	1.1	other causes	Malnutrition	Released
2075	Caretta caretta	2024	2024-05-20	2024-06-08	41.5	12.4	Infectious disease		Released
2076	Caretta caretta	2024	2024-05-23	2024-07-23	31.5	3.2	Entanglement		Released
2077	Caretta caretta	2024	2024-05-28	2024-06-04	22	1.4	other causes	Malnutrition	Died during hospitalization
2078	Caretta caretta	2024	2024-05-29	2024-05-29	46	13.7	other causes	Malnutrition	Died during hospitalization
2079	Caretta caretta	2024	2024-05-29	2024-06-30	41.5	13.8	Unknown/undetermined		Released
2080	Caretta caretta	2024	2024-05-30	2024-06-02	24	1.6	other causes	Buoyancy disorder	Died during hospitalization
2081	Caretta caretta	2024	2024-05-31				trauma		Admitted dead
2082	Caretta caretta	2024	2024-06-01		17	0.8	Unknown/undetermined		Admitted dead

Record ID	Species	Year	Admission date	Departure date	SCL (cm)	Body weight (kg)	Cause of admission	Other-cause subcategory	Final disposition
2083	Caretta caretta	2024	2024-06-19		34.2	3.5	Unknown/undetermined		Admitted dead
2084	Caretta caretta	2024	2024-07-03				trauma		Admitted dead
2085	Caretta caretta	2024	2024-07-03				Entanglement		Admitted dead
2086	Caretta caretta	2024	2024-07-09	2024-12-11	25.5	2.5	other causes	Malnutrition	Released
2087	Caretta caretta	2024	2024-07-10				Unknown/undetermined		Admitted dead
2088	Caretta caretta	2024	2024-07-16	2024-07-23	46.5	17.5	other causes	Buoyancy disorder	Released
2089	Caretta caretta	2024	2024-07-19	2024-08-09	35	5.8	Entanglement		Released
2090	Caretta caretta	2024	2024-07-19	2024-07-31	41	9.3	Entanglement		Released
2091	Caretta caretta	2024	2024-07-20	2024-10-24	48.5	20.2	Hooks/monofilament lines		Released
2092	Caretta caretta	2024	2024-07-21	2024-08-02	31	3.6	Unknown/undetermined		Died during hospitalization
2093	Caretta caretta	2024	2024-07-22	2024-08-21	31	5	Unknown/undetermined		Released
2094	Caretta caretta	2024	2024-07-22	2024-07-31	38	9.2	Unknown/undetermined		Released
2095	Caretta caretta	2024	2024-07-22	2024-08-09			Entanglement		Released
2096	Caretta caretta	2024	2024-07-23		60	30	Unknown/undetermined		Admitted dead
2097	Caretta caretta	2024	2024-07-24	2024-08-14	25.5	2.6	other causes	Buoyancy disorder	Released
2098	Caretta caretta	2024	2024-07-25				Unknown/undetermined		Admitted dead
2099	Caretta caretta	2024	2024-07-25		100	60	Unknown/undetermined		Admitted dead
2100	Caretta caretta	2024	2024-07-28	2024-08-06	34.5	7	Unknown/undetermined		Released
2101	Caretta caretta	2024	2024-07-28	2024-08-06	39	8.8	Entanglement		Released
2102	Caretta caretta	2024	2024-07-29				Entanglement		Admitted dead
2103	Caretta caretta	2024	2024-07-30		90	65	Hooks/monofilament lines		Admitted dead
2104	Caretta caretta	2024	2024-07-31	2024-08-21		1.4	other causes	Buoyancy disorder	Released
2105	Caretta caretta	2024	2024-08-03	2024-10-19	37	7	Entanglement		Released
2106	Caretta caretta	2024	2024-08-07	2024-09-27	20	1.2	Entanglement		Released
2107	Caretta caretta	2024	2024-08-07	2024-09-06	36.5	8	Entanglement		Released
2108	Caretta caretta	2024	2024-08-07	2024-08-21	37	8.1	Entanglement		Released
2109	Caretta caretta	2024	2024-08-10	2025-01-14	25	2.4	Entanglement		Released
2110	Caretta caretta	2024	2024-08-12	2024-08-29	18	0.9	Unknown/undetermined		Released
2111	Caretta caretta	2024	2024-08-13		36	1.8	Unknown/undetermined		Admitted dead
2112	Caretta caretta	2024	2024-08-18		51	19.6	Unknown/undetermined		Admitted dead
2113	Caretta caretta	2024	2024-08-18	2024-12-11	25	2.5	Entanglement		Released
2114	Caretta caretta	2024	2024-08-21	2024-12-11	32.5	5.1	Entanglement		Released
2115	Caretta caretta	2024	2024-08-23	2024-09-23	35	8.6	Unknown/undetermined		Released
2116	Caretta caretta	2024	2024-08-23		45	8.7	Unknown/undetermined		Admitted dead
2117	Caretta caretta	2024	2024-08-23	2024-09-04	22	1.6	Entanglement		Released
2118	Caretta caretta	2024	2024-08-26	2024-10-05	15	0.8	other causes	Buoyancy disorder	Released
2119	Caretta caretta	2024	2024-08-26	2024-08-31	31	4.5	other causes	Buoyancy disorder	Released
2120	Caretta caretta	2024	2024-08-26			40	Unknown/undetermined		Admitted dead
2121	Caretta caretta	2024	2024-08-26		80	40	Unknown/undetermined		Admitted dead
2122	Caretta caretta	2024	2024-08-27	2024-09-10	36	6.4	Entanglement		Released
2123	Caretta caretta	2024	2024-08-27	2024-09-04	42	10.2	Entanglement		Released
2124	Caretta caretta	2024	2024-08-27			30	Entanglement		Admitted dead
2125	Caretta caretta	2024	2024-09-01	2024-09-06	20	1.3	other causes	Buoyancy disorder	Released
2126	Caretta caretta	2024	2024-09-02	2024-09-27	34	6.3	Hooks/monofilament lines		Released
2127	Caretta caretta	2024	2024-09-02	2024-09-06	39	8.2	Entanglement		Released
2128	Caretta caretta	2024	2024-09-03	2024-09-25	35	6.5	Unknown/undetermined		Released
2129	Caretta caretta	2024	2024-09-04	2024-09-10	20	1.4	other causes	Buoyancy disorder	Released
2130	Caretta caretta	2024	2024-09-04	2024-09-10	40	10.6	Entanglement		Released
2131	Caretta caretta	2024	2024-09-05	2024-09-20	21.5	1.8	Unknown/undetermined		Released
2132	Caretta caretta	2024	2024-09-19	2024-10-24	55	23.9	other causes	Malnutrition	Released
2133	Caretta caretta	2024	2024-09-21		21	1.6	Unknown/undetermined		Admitted dead
2134	Caretta caretta	2024	2024-09-25				Unknown/undetermined		Admitted dead
2135	Caretta caretta	2024	2024-09-27	2024-10-18	32.5	5	other causes	Malnutrition	Released
2136	Caretta caretta	2024	2024-09-27	2024-10-27	16.5	0.8	Entanglement		Died during hospitalization
2137	Caretta caretta	2024	2024-09-30		40	7.7	Unknown/undetermined		Admitted dead
2138	Caretta caretta	2024	2024-10-03		55	25	Unknown/undetermined		Admitted dead
2139	Caretta caretta	2024	2024-10-04		31	3	Unknown/undetermined		Admitted dead
2140	Caretta caretta	2024	2024-10-18	2024-10-24	40	11.2	Entanglement		Released
2141	Caretta caretta	2024	2024-10-26	2025-01-30	24.5	2.8	other causes	Malnutrition	Died during hospitalization
2142	Caretta caretta	2024	2024-12-02	2025-01-14	42	12.7	Entanglement		Released
2143	Caretta caretta	2024	2024-12-07	2024-12-18	47	17.8	trauma		Died during hospitalization
2144	Caretta caretta	2024	2024-12-09		67	30	Unknown/undetermined		Admitted dead
2145	Caretta caretta	2024	2024-12-21	2024-12-26	58	28.4	other causes	Malnutrition	Died during hospitalization
2146	Caretta caretta	2025	2025-01-14	2025-02-05			Unknown/undetermined		Released
2147	Caretta caretta	2025	2025-01-25	2025-02-02	42	8.4	Hooks/monofilament lines		Died during hospitalization
2148	Caretta caretta	2025	2025-02-07	2025-04-23	67	45.2	trauma		Died during hospitalization
2149	Caretta caretta	2025	2025-02-12		32.5		Entanglement		Admitted dead
2150	Caretta caretta	2025	2025-02-13	2025-03-17	37.8	9.1	Infectious disease		Released
2151	Caretta caretta	2025	2025-02-14	2025-02-17	40	10.8	other causes	Miscellany	Released
2152	Caretta caretta	2025	2025-02-20				Unknown/undetermined		Admitted dead
2153	Caretta caretta	2025	2025-02-22	2025-12-19			trauma		Released
2154	Caretta caretta	2025	2025-02-25				Unknown/undetermined		Admitted dead

Record ID	Species	Year	Admission date	Departure date	SCL (cm)	Body weight (kg)	Cause of admission	Other-cause subcategory	Final disposition
2155	Caretta caretta	2025	2025-02-27		47	15	Unknown/undetermined		Admitted dead
2156	Caretta caretta	2025	2025-03-06	2025-05-16	62	44.8	trauma		Released
2157	Caretta caretta	2025	2025-03-14	2025-06-08	32	5.2	Entanglement		Released
2158	Caretta caretta	2025	2025-03-23	2025-05-16	33	5.7	Entanglement		Released
2159	Caretta caretta	2025	2025-03-28	2025-04-25	28	4.3	other causes	Malnutrition	Released
2160	Caretta caretta	2025	2025-04-15	2025-11-24	63	31.6	other causes	Malnutrition	Euthanized
2161	Caretta caretta	2025	2025-04-18	2025-04-25	72	49.2	other causes	Malnutrition	Released
2162	Caretta caretta	2025	2025-05-02		30	2.1	Unknown/undetermined		Admitted dead
2163	Caretta caretta	2025	2025-05-03	2025-05-16	55	26.1	Entanglement		Released
2164	Caretta caretta	2025	2025-05-05	2025-06-08	36.5	7.2	Hooks/monofilament lines		Released
2165	Caretta caretta	2025	2025-05-06		24	2.6	Unknown/undetermined		Admitted dead
2166	Caretta caretta	2025	2025-05-25			35	Unknown/undetermined		Admitted dead
2167	Caretta caretta	2025	2025-05-27	2025-06-25	38	9.6	other causes	Malnutrition	Released
2168	Caretta caretta	2025	2025-05-27		45.5	15.5	Unknown/undetermined		Admitted dead
2169	Caretta caretta	2025	2025-06-01	2025-06-25	39.5	11.9	Entanglement		Released
2170	Caretta caretta	2025	2025-06-02		27	3.3	Unknown/undetermined		Admitted dead
2171	Caretta caretta	2025	2025-06-04				Unknown/undetermined		Admitted dead
2172	Caretta caretta	2025	2025-06-10	2025-06-25	53	19.6	other causes	Buoyancy disorder	Released
2173	Caretta caretta	2025	2025-06-12				Unknown/undetermined		Admitted dead
2174	Caretta caretta	2025	2025-06-17	2025-07-25	37	7.7	other causes	Malnutrition	Released
2175	Caretta caretta	2025	2025-06-20	2025-07-23	35	7.7	Entanglement		Released
2176	Caretta caretta	2025	2025-06-30		23	3.9	Entanglement		Admitted dead
2177	Caretta caretta	2025	2025-07-07	2025-09-20	39	10	Entanglement		Released
2178	Caretta caretta	2025	2025-07-08		85	31.3	Hooks/monofilament lines		Admitted dead
2179	Caretta caretta	2025	2025-07-09	2025-07-23	40	11.1	Unknown/undetermined		Released
2180	Caretta caretta	2025	2025-07-09		52	20.4	Unknown/undetermined		Admitted dead
2181	Caretta caretta	2025	2025-07-10				Unknown/undetermined		Admitted dead
2182	Caretta caretta	2025	2025-07-13	2025-08-09	20.5	1.2	Entanglement		Released
2183	Caretta caretta	2025	2025-07-16		60	38.2	Unknown/undetermined		Admitted dead
2184	Caretta caretta	2025	2025-07-16	2025-08-13	30	4.5	Entanglement		Released
2185	Caretta caretta	2025	2025-07-23	2025-09-10	19	1.2	other causes	Malnutrition	Released
2186	Caretta caretta	2025	2025-07-23	2025-09-23	33.5	5.4	other causes	Malnutrition	Released
2187	Caretta caretta	2025	2025-07-23	2025-08-08	38	8.1	Unknown/undetermined		Released
2188	Caretta caretta	2025	2025-07-27	2025-09-21	19	1.1	other causes	Malnutrition	Released
2189	Caretta caretta	2025	2025-07-29	2025-10-17	35	6.2	Entanglement		Released
2190	Caretta caretta	2025	2025-07-31	2025-08-13	30	4.6	other causes	Buoyancy disorder	Released
2191	Caretta caretta	2025	2025-08-05	2025-08-20	29	3.6	Entanglement		Released
2192	Caretta caretta	2025	2025-08-06	2025-08-31		6.6	Unknown/undetermined		Released
2193	Caretta caretta	2025	2025-08-06		39	7.8	Unknown/undetermined		Admitted dead
2194	Caretta caretta	2025	2025-08-10			1.1	Unknown/undetermined		Admitted dead
2195	Caretta caretta	2025	2025-08-11				other causes	Miscellany	Admitted dead
2196	Caretta caretta	2025	2025-08-14	2025-08-14	41	9.9	Entanglement		Released
2197	Caretta caretta	2025	2025-08-14				Entanglement		Admitted dead
2198	Caretta caretta	2025	2025-08-15	2025-09-10	40.5	10.4	Entanglement		Released
2199	Caretta caretta	2025	2025-08-20	2025-10-15	19	1.2	Entanglement		Released
2200	Caretta caretta	2025	2025-08-29	2025-09-10	31	4.8	Entanglement		Released
2201	Caretta caretta	2025	2025-09-06		55	30	Unknown/undetermined		Admitted dead
2202	Caretta caretta	2025	2025-09-06	2025-11-24	35	7.1	Entanglement		Euthanized
2203	Caretta caretta	2025	2025-09-10	2025-10-10	38	7	Entanglement		Released
2204	Caretta caretta	2025	2025-09-11	2025-10-10	35	6.1	Entanglement		Released
2205	Caretta caretta	2025	2025-09-21	2025-09-27	37	8	Entanglement		Released
2206	Caretta caretta	2025	2025-09-29	2025-11-20	19	1.4	Entanglement		Released
2207	Caretta caretta	2025	2025-09-29	2025-11-10	22	1.6	Entanglement		Released
2208	Caretta caretta	2025	2025-10-01	2025-10-29	37	6.8	Entanglement		Released
2209	Caretta caretta	2025	2025-10-02	2025-10-29	35	6.3	Entanglement		Released
2210	Caretta caretta	2025	2025-10-07	2025-10-29	30	4.5	Entanglement		Released
2211	Caretta caretta	2025	2025-10-09	2025-10-15	21.5	1.8	Entanglement		Released
2212	Caretta caretta	2025	2025-10-24	2026-03-30			Entanglement		Released
2213	Caretta caretta	2025	2025-11-05	2025-12-19			Entanglement		Released
2214	Caretta caretta	2025	2025-11-08	2026-03-17			Entanglement		Released
2215	Caretta caretta	2025	2025-12-04				Unknown/undetermined		Admitted dead
2216	Caretta caretta	2025	2025-12-30	2026-03-17			Entanglement		Released

Chelonia mydas

Record ID	Species	Year	Admission date	Departure date	SCL (cm)	Body weight (kg)	Cause of admission	Other-cause subcategory	Final disposition
2217	Chelonia mydas	2000	2000-09-07	2000-10-09			Hooks/monofilament lines		Released
2218	Chelonia mydas	2002	2002-05-26				trauma		Admitted dead
2219	Chelonia mydas	2004	2004-09-30	2004-10-21			Unknown/undetermined		Released
2220	Chelonia mydas	2005	2005-05-20	2005-06-01			Hooks/monofilament lines		Released
2221	Chelonia mydas	2006	2006-10-09	2006-10-12			Hooks/monofilament lines		Released
2222	Chelonia mydas	2007	2007-05-09	2007-05-17			other causes	Miscellany	Released

Record ID	Species	Year	Admission date	Departure date	SCL (cm)	Body weight (kg)	Cause of admission	Other-cause subcategory	Final disposition
2223	Chelonia mydas	2007	2007-08-21	2007-08-25			Hooks/monofilament lines		Released
2224	Chelonia mydas	2008	2008-07-11				trauma		Admitted dead
2225	Chelonia mydas	2008	2008-09-05	2008-10-15			Hooks/monofilament lines		Released
2226	Chelonia mydas	2008	2008-09-29	2008-11-03			Hooks/monofilament lines		Released
2227	Chelonia mydas	2008	2008-12-11	2008-12-16			trauma		Released
2228	Chelonia mydas	2009	2009-05-25	2009-06-15			Hooks/monofilament lines		Released
2229	Chelonia mydas	2010	2010-03-28	2010-03-29			trauma		Died during hospitalization
2230	Chelonia mydas	2010	2010-04-21	2010-06-01			Hooks/monofilament lines		Released
2231	Chelonia mydas	2010	2010-09-30				Hooks/monofilament lines		Admitted dead
2232	Chelonia mydas	2010	2010-11-17	2010-12-02			Hooks/monofilament lines		Released
2233	Chelonia mydas	2010	2010-11-28	2010-12-15			Entanglement		Released
2234	Chelonia mydas	2011	2011-07-23	2011-08-19			trauma		Released
2235	Chelonia mydas	2011	2011-12-07				Unknown/undetermined		Admitted dead
2236	Chelonia mydas	2012	2012-03-31	2012-04-22	66	51	Infectious disease		Died during hospitalization
2237	Chelonia mydas	2012	2012-04-05	2012-05-04	54.5		trauma		Released
2238	Chelonia mydas	2012	2012-10-01	2012-10-17	32	4.7	Unknown/undetermined		Released
2239	Chelonia mydas	2013	2013-07-26	2013-08-01	33.5	6.2	Infectious disease		Released
2240	Chelonia mydas	2013	2013-10-20	2013-11-13	40.2	9.1	Hooks/monofilament lines		Released
2241	Chelonia mydas	2013	2013-10-27		39	10.8	Unknown/undetermined		Admitted dead
2242	Chelonia mydas	2014	2014-01-21	2014-02-07	57.4	28.8	Hooks/monofilament lines		Released
2243	Chelonia mydas	2014	2014-01-28	2014-02-07	41.9		Hooks/monofilament lines		Released
2244	Chelonia mydas	2014	2014-04-29	2014-05-01	53	20.6	trauma		Died during hospitalization
2245	Chelonia mydas	2014	2014-07-31				trauma		Admitted dead
2246	Chelonia mydas	2014	2014-08-02	2014-08-31	47.3		Hooks/monofilament lines		Released
2247	Chelonia mydas	2014	2014-11-27	2014-12-11	40	8.8	trauma		Released
2248	Chelonia mydas	2015	2015-08-19				trauma		Admitted dead
2249	Chelonia mydas	2016	2016-01-17		59		Unknown/undetermined		Admitted dead
2250	Chelonia mydas	2016	2016-08-26				Entanglement		Admitted dead
2251	Chelonia mydas	2016	2016-09-07		36.5	6.1	trauma		Admitted dead
2252	Chelonia mydas	2017	2017-04-19				trauma		Admitted dead
2253	Chelonia mydas	2017	2017-06-11	2017-08-07	35.5	5.3	Hooks/monofilament lines		Released
2254	Chelonia mydas	2018	2018-03-13	2018-06-27	60	30.9	trauma		Released
2255	Chelonia mydas	2018	2018-07-31		69		Unknown/undetermined		Admitted dead
2256	Chelonia mydas	2018	2018-09-14	2018-10-19	76	56.7	Infectious disease		Released
2257	Chelonia mydas	2019	2019-01-07	2019-01-30	60.8	30.8	Hooks/monofilament lines		Released
2258	Chelonia mydas	2019	2019-05-03				Entanglement		Admitted dead
2259	Chelonia mydas	2019	2019-08-29		30		Unknown/undetermined		Admitted dead
2260	Chelonia mydas	2019	2019-10-19		35	3.7	Unknown/undetermined		Admitted dead
2261	Chelonia mydas	2020	2020-02-02		38.5	8.2	trauma		Admitted dead
2262	Chelonia mydas	2020	2020-06-20				Unknown/undetermined		Admitted dead
2263	Chelonia mydas	2021	2021-08-09				trauma		Admitted dead
2264	Chelonia mydas	2022	2022-01-22	2022-02-08	47.6	11.1	Hooks/monofilament lines		Released
2265	Chelonia mydas	2022	2022-04-06		42.8	8.8	Unknown/undetermined		Admitted dead
2266	Chelonia mydas	2022	2022-05-13	2022-06-01	34	4.6	Entanglement		Released
2267	Chelonia mydas	2023	2023-10-11	2023-10-16	36	6.2	Hooks/monofilament lines		Released
2268	Chelonia mydas	2023	2023-12-28	2024-01-03	31.5	3.6	other causes	Malnutrition	Died during hospitalization
2269	Chelonia mydas	2024	2024-12-04		48	12.1	Unknown/undetermined		Admitted dead
2270	Chelonia mydas	2025	2025-10-01	2025-10-06	42	10.7	Hooks/monofilament lines		Released

Section 2. Readmission events

This section includes the 22 readmission events recorded for 16 of the 2,270 individual turtles listed in Section 1. The Record ID is the same unique individual identifier used in Section 1. Each row represents a readmission event, not a new individual. For second and subsequent readmissions, 'Previous cause' refers to the immediately preceding admission/readmission event.

Record ID	Species	Readmission no.	Previous release date	Readmission date	Interval (days)	Previous cause	Readmission cause	Cause vs previous	Status at readmission	Final disposition	Departure date	SCL (cm)	Body weight (kg)
374	Caretta caretta	1	2003-08-11	2003-08-14	3	Trauma (collisions)	Trauma (collisions)	Same	Alive	Released	2003-08-15		
549	Caretta caretta	1	2006-06-20	2006-06-22	2	Infectious disease	Buoyancy disorder	Different	Alive	Released	2006-07-10		
549	Caretta caretta	2	2006-07-10	2006-07-20	10	Buoyancy disorder	Buoyancy disorder	Same	Alive	Released	2006-07-21		
549	Caretta caretta	3	2006-07-21	2006-07-22	1	Buoyancy disorder	Buoyancy disorder	Same	Alive	Released	2006-07-28		
549	Caretta caretta	4	2006-07-28	2006-07-30	2	Buoyancy disorder	Buoyancy disorder	Same	Alive	Released	2006-08-04		
588	Caretta caretta	1	2006-09-19	2006-09-27	8	Trauma (collisions)	Trauma (collisions)	Same	Alive	Released	2006-09-27		
1165	Caretta caretta	1	2014-09-11	2014-10-08	27	Unknown/undetermined	Trauma (collisions)	Different	Dead	Dead on admission		41	11.5
1262	Caretta caretta	1	2015-11-10	2016-02-25	107	Hooks/monofilament lines	Unknown/undetermined	Different	Alive	Released	2016-03-08	53.5	28
1360	Caretta caretta	1	2017-01-18	2017-01-21	3	Hooks/monofilament lines	Buoyancy disorder	Different	Alive	Released	2017-03-23	59	39
1414	Caretta caretta	1	2018-05-23	2018-06-02	10	Miscellany	Malnutrition	Different	Alive	Released	2018-07-01	38	7.5
1503	Caretta caretta	1	2019-03-29	2019-09-10	165	Infectious disease	Infectious disease	Same	Alive	Released	2019-09-14	54.5	23
1503	Caretta caretta	2	2019-09-14	2020-11-11	424	Infectious disease	Malnutrition	Different	Alive	Released	2020-12-02	54.5	23
1503	Caretta caretta	3	2020-12-02	2021-04-24	143	Malnutrition	Infectious disease	Different	Alive	Released	2021-07-13		
1510	Caretta caretta	1	2019-04-11	2019-04-17	6	Malnutrition	Infectious disease	Different	Alive	Released	2019-04-28	54	
1600	Caretta caretta	1	2019-08-28	2021-08-18	721	Hooks/monofilament lines	Unknown/undetermined	Different	Dead	Dead on admission			
1807	Caretta caretta	1	2021-07-19	2021-08-10	22	Hooks/monofilament lines	Infectious disease	Different	Alive	Released	2021-09-09		
1981	Caretta caretta	1	2023-06-02	2023-06-02	0	Unknown/undetermined	Infectious disease	Different	Alive	Released	2023-06-09	40	10
2088	Caretta caretta	1	2024-07-23	2024-11-22	122	Buoyancy disorder	Buoyancy disorder	Same	Alive	Released	2024-12-11		
2237	Chelonia mydas	1	2012-05-04	2016-03-29	1425	Trauma (collisions)	Infectious disease	Different	Alive	Released	2016-04-22	65	54.2
2238	Chelonia mydas	1	2012-10-17	2016-01-25	1195	Unknown/undetermined	Trauma (collisions)	Different	Alive	Released	2016-03-16	54.5	22
2238	Chelonia mydas	2	2016-03-16	2018-07-26	862	Trauma (collisions)	Hooks/monofilament lines	Different	Alive	Released	2018-10-10		
2242	Chelonia mydas	1	2014-02-07	2016-07-25	899	Hooks/monofilament lines	Hooks/monofilament lines	Same	Alive	Released	2016-07-29	57	24
