# Supplementary material for: Benchmark indicators for evaluating sea turtle rehabilitation outcomes at a wildlife rehabilitation center on Tenerife island, Spain (1982–2025)
Source: Front Vet Sci. 2026 Oct 19;13:1948954. doi: 10.3389/fvets.2026.1948954 (PMC13726524; doi:10.3389/fvets.2026.1948954)
Supplement: Supplementary file 2 [file Table_1.pdf]

Supplementary Table S1. Summary of selected studies reporting rehabilitation outcomes and post-release findings in sea turtles.

Location and study period	Species	Sample size	Main causes/conditions	Rehabilitation outcome	Rehabilitation duration	Post-release findings	Reference
Florida, USA; 1986–2004	*C. mydas*<br>*C. caretta*<br>*L. kempii*	1,700 admitted	NR	36.8% released<br>61.5% dead<br>1.6% permanent captivity	Rehabilitation often required several months; some cases > 3 years	NR	Baker *et al.* (2015) (8)
Gran Canaria Island, Spain; 1998–2014	*C. caretta*	1,634 admitted	Entanglement (56.2%)<br>Unknown (13.5%)<br>Hooks/monofilament lines (12.7%)<br>Infectious disease (6.2%)<br>Trauma (4.6%)	86.3% released<br>10.3% dead<br>3.4% euthanized	Median time for released ranged from 12 days (unknown) to 70 days (trauma)	NR	Orós *et al.* (2016) (6)
Queensland, Australia; 1996–2013	Multiple species, predominantly *C. mydas*	2,970 admitted	Unknown (54%)<br>Disease (18%)<br>Buoyancy disorder (13%)<br>Fracture (6%)	39% released	Average time in care before release decreased from 392 days (1999) to 84 days (2013)	101 released turtles subsequently re-encountered	Flint *et al.* (2017) (11)
USA; releases through 2016 (including pre-1997 releases)	Multiple species	11,417 released	NR	NA	NR	314 first re-encounters documented; post-release information unavailable for most released turtles	Innis *et al.* (2019) (13)
New York, USA; 1998–2019	*L. kempii*<br>*C. mydas*<br>*C. caretta*	222 admitted	All cold-stunned turtles	59% released	NR	5 turtles restranded again due to cold-stunning	Montello *et al.* (2022) (14)
Maldives; 2010–2022	Multiple species, predominantly *L. olivacea*	319 admitted	Entanglement in ghost nets (61%)	65.1% released	Average time to release: 70 days	NR	Himpson *et al.* (2023) (15)

NA: not applicable; NR: not reported
