# Supplementary material for: Benchmark indicators for evaluating sea turtle rehabilitation outcomes at a wildlife rehabilitation center on Tenerife island, Spain (1982–2025)
Source: Front Vet Sci. 2026 Oct 19;13:1948954. doi: 10.3389/fvets.2026.1948954 (PMC13726524; doi:10.3389/fvets.2026.1948954)
Supplement: Supplementary file 3 [file Table_2.pdf]

Supplementary Table S2. Number of cases and frequency distribution of causes at first admission by straight carapace length (SCL) category in loggerhead and green turtles during the study period (1982–2025).

Cause of admission	Loggerheads					Green turtles					Total	
	SCL < 42 cm	42 cm ≤ SCL < 70 cm	SCL ≥ 70 cm	Unknown SCL	Total n (%)	SCL < 65 cm	65 cm ≤ SCL < 90 cm	SCL ≥ 90 cm	Unknown SCL	Total n (%)	n	%
**Entanglement**	369	56	3	539	967 (43.6)	1	0	0	3	4 (7.4)	971	42.8
**Hooks/monofilament lines**	7	34	3	80	124 (5.6)	9	0	0	10	19 (35.2)	143	6.3
**Trauma (collisions)**	16	18	3	46	83 (3.7)	6	0	0	9	15 (27.8)	98	4.3
**Infectious disease**	80	30	0	106	216 (9.7)	1	2	0	0	3 (5.6)	219	9.6
**Crude oil**	4	2	0	51	57 (2.6)	0	0	0	0	0 (0)	57	2.5
**Other causes**	184	38	2	69	293 (13.2)	1	0	0	1	2 (3.7)	295	13
Plastic ingestion	1	1	0	1	3 (0.1)	0	0	0	0	0 (0)	3	0.1
Buoyancy disorder	34	12	0	9	55 (2.5)	0	0	0	0	0 (0)	55	2.4
Shark attack	3	1	0	4	8 (0.4)	0	0	0	0	0 (0)	8	0.4
Malnutrition	100	16	2	33	151 (6.8)	1	0	0	0	1 (1.9)	152	6.7
Miscellany	46	8	0	22	76 (3.4)	0	0	0	1	1 (1.9)	77	3.4
**Unknown/undetermined**	155	59	8	254	476 (21.5)	7	1	0	3	11 (20.4)	487	21.5
**TOTAL**	**815**	**237**	**19**	**1,145**	**2,216**	**25**	**3**	**0**	**26**	**54**	**2,270**	**100**
