# Supplementary material for: Benchmark indicators for evaluating sea turtle rehabilitation outcomes at a wildlife rehabilitation center on Tenerife island, Spain (1982–2025)
Source: Front Vet Sci. 2026 Oct 19;13:1948954. doi: 10.3389/fvets.2026.1948954 (PMC13726524; doi:10.3389/fvets.2026.1948954)
Supplement: Supplementary file 4 [file Table_3.pdf]

Supplementary Table S3. Post hoc comparisons of causes at first admission and causes of mortality in turtles dead on first admission between loggerhead and green sea turtles.

	**Loggerhead turtles n (%)**	**Green turtles n (%)**	**OR, green vs. loggerhead (95% CI)**	**Adjusted *P* value**
**Causes of admission**				
**Entanglement**	967 (43.6)	4 (7.4)	0.10 (0.04–0.29)	<0.001
**Hooks/monofilament lines**	124 (5.6)	19 (35.2)	9.16 (5.09–16.48)	<0.001
**Trauma (collisions)**	83 (3.7)	15 (27.8)	9.88 (5.24–18.64)	<0.001
**Infectious disease**	216 (9.7)	3 (5.6)	0.54 (0.17–1.76)	1.000
**Crude oil**	57 (2.6)	0 (0.0)	NE	1.000
**Other causes**	293 (13.2)	2 (3.7)	0.25 (0.06–1.04)	0.155
**Unknown/undetermined**	476 (21.5)	11 (20.4)	0.94 (0.48–1.83)	1.000
**Causes of mortality in turtles dead on admission**				
**Entanglement**	83 (19)	2 (10.0)	0.47 (0.11–2.08)	1.000
**Hooks/monofilament lines**	14 (3.2)	1 (5.0)	1.59 (0.20–12.73)	1.000
**Trauma (collisions)**	33 (7.6)	8 (40.0)	8.16 (3.12–21.37)	<0.001
**Infectious disease**	1 (0.2)	0 (0.0)	NE	1.000
**Crude oil**	15 (3.4)	0 (0.0)	NE	1.000
**Other causes**	8 (1.8)	0 (0.0)	NE	1.000
**Unknown/undetermined**	283 (64.8)	9 (45.0)	0.45 (0.18–1.10)	0.566

Odds ratios (ORs) were calculated for green turtles relative to loggerhead turtles; therefore, OR > 1 indicates greater odds in green turtles, whereas OR < 1 indicates greater odds in loggerhead turtles. For each post hoc comparison, the corresponding category was compared with all remaining categories combined using Fisher's exact test. *P* values were adjusted for multiple comparisons within each analysis using the Holm method. NE: not estimable because of zero cell counts; CI: confidence interval.
