# Supplementary material for: Benchmark indicators for evaluating sea turtle rehabilitation outcomes at a wildlife rehabilitation center on Tenerife island, Spain (1982–2025)
Source: Front Vet Sci. 2026 Oct 19;13:1948954. doi: 10.3389/fvets.2026.1948954 (PMC13726524; doi:10.3389/fvets.2026.1948954)
Supplement: Supplementary file 5 [file Table_4.pdf]

Supplementary Table S4. Period-adjusted associations between cause at first admission and rehabilitation outcomes in sea turtles alive at first admission.

Cause of admission	Unadjusted OR (95% CI)	Period-adjusted OR (95% CI)	Adjusted *P* value
**Unassisted mortality during hospitalization**			
Entanglement	0.46 (0.34–0.62)	0.45 (0.34–0.60)	<0.001
Hooks/monofilament lines	2.80 (1.84–4.26)	2.71 (1.78–4.14)	<0.001
Trauma (collisions)	2.86 (1.58–5.18)	2.81 (1.55–5.10)	0.003
Infectious disease	2.62 (1.85–3.71)	2.60 (1.83–3.68)	<0.001
Crude oil	NE	NE	NE
Other causes	0.86 (0.58–1.27)	0.93 (0.62–1.40)	1.000
Unknown/undetermined	0.69 (0.41–1.14)	0.69(0.42–1.15)	0.464
**Release**			
Entanglement	2 (1.52–2.64)	2.09 (1.58–2.77)	<0.001
Hooks/monofilament lines	0.36 (0.24–0.54)	0.38 (0.25–0.58)	<0.001
Trauma (collisions)	0.26 (0.15–0.45)	0.27 (0.15–0.47)	<0.001
Infectious disease	0.43 (0.31–0.61)	0.44 (0.31–0.62)	<0.001
Crude oil	NE	NE	NE
Other causes	1.16 (0.79–1.69)	1 (0.67–1.48)	1.000
Unknown/undetermined	1.67 (1.01–2.77)	1.63 (0.98–2.71)	0.172

OR: odds ratio; CI: confidence interval; NE: not estimable because of complete separation caused by zero cell counts. For each cause-specific analysis, the corresponding admission category was compared with all remaining admission categories combined. Period-adjusted ORs were estimated using binary logistic regression with admission period (1982–2009, 2010–2019, and 2020–2025) included as a categorical covariate. *P* values for the cause-specific adjusted comparisons were corrected for multiple comparisons within each outcome using the Holm method. Unadjusted ORs are shown for comparison with the primary analyses.
