# Supplementary material for: Benchmark indicators for evaluating sea turtle rehabilitation outcomes at a wildlife rehabilitation center on Tenerife island, Spain (1982–2025)
Source: Front Vet Sci. 2026 Oct 19;13:1948954. doi: 10.3389/fvets.2026.1948954 (PMC13726524; doi:10.3389/fvets.2026.1948954)
Supplement: Supplementary file 6 [file Table_5.pdf]

Supplementary Table S5. Descriptive statistics of time from admission to final disposition in loggerhead and green turtle hospitalization episodes by cause of admission during the study period (1982–2025).

	Cause of admission	Time (days) from admission to final disposition														
		Euthanasia					Unassisted mortality					Release				
		P_{10}	P_{25}	P_{50}	P_{75}	P_{90}	P_{10}	P_{25}	P_{50}	P_{75}	P_{90}	P_{10}	P_{25}	P_{50}	P_{75}	P_{90}
LOGGERHEAD TURTLES	**Entanglement**	0	1	2	30.5	88.6	1	4	11	30.7	59.5	6	12	23	43	69
	**Hooks/monofilament lines**	0	0	1	-	-	1	1	3	9.2	28.5	5	12.5	22	41.5	89.6
	**Trauma (collisions)**	0	0.5	5	79	-	0	0.7	3.5	25.2	86.5	3.4	6.5	41	107.5	262.8
	**Infectious disease**	N/A	N/A	N/A	N/A	N/A	1	1.2	6.5	22.5	39	7	12	22	47	88.4
	**Crude oil**											2.3	5	10	21.5	54.3
	**Other causes**	0	0.2	1.5	167.7	-	0.2	3	8	31	67	4	8	16	31	53.3
	Plastic ingestion						N/A	N/A	N/A	N/A	N/A	6	6	10.5	-	-
	Buoyancy disorder						3	3	20	-	-	5	7.2	14	23	44.6
	Shark attack	0	0	0.5	-	-	N/A	N/A	N/A	N/A	N/A	25	25	40	47	-
	Malnutrition	N/A	N/A	N/A	N/A	N/A	0.8	3	8	31	68	5	13.8	24	40.2	78
	Miscellany	N/A	N/A	N/A	N/A	N/A						3	5	9	15.5	40.6
	**Unknown/undetermined**						0	1	5.5	19.5	53.3	1	5	14	26	63.9

Supplementary Table S5 (Continued). Descriptive statistics of time from admission to final disposition in loggerhead and green turtle hospitalization episodes by cause of admission during the study period (1982–2025).

	Cause of admission	Time (days) from admission to final disposition														
		Euthanasia					Unassisted mortality					Release				
		P_{10}	P_{25}	P_{50}	P_{75}	P_{90}	P_{10}	P_{25}	P_{50}	P_{75}	P_{90}	P_{10}	P_{25}	P_{50}	P_{75}	P_{90}
GREEN TURTLES	**Entanglement**											17	17	18	-	-
	**Hooks/monofilament lines**											4	6.2	19	34.2	55.4
	**Trauma (collisions)**						1	1	1.5	-	-	5	11.8	28	64.8	-
	**Infectious disease**						N/A	N/A	N/A	N/A	N/A	6	6	24	-	-
	**Crude oil**															
	**Other causes**						N/A	N/A	N/A	N/A	N/A	N/A	N/A	N/A	N/A	N/A
	Plastic ingestion															
	Buoyancy disorder															
	Shark attack															
	Malnutrition						N/A	N/A	N/A	N/A	N/A					
	Miscellany											N/A	N/A	N/A	N/A	N/A
	**Unknown/undetermined**											16	16	18.5	-	-

P_{10}, P_{25}, P_{50}, P_{75}, P_{90}: 10th, 25th, 50th (median), 75th and 90th percentiles; N/A: not applicable because only one observation was available; -: percentile could not be estimated because of the limited number of observations; Blank cells indicate that no observations were available for the corresponding outcome. Hospitalization episodes include first admissions and subsequent readmissions of turtles admitted alive.
